# EBF1 primes B-lymphoid enhancers and limits the myeloid bias in murine multipotent progenitors

**DOI:** 10.1084/jem.20212437

**Published:** 2022-09-01

**Authors:** Aurelie Lenaerts, Iwo Kucinski, Ward Deboutte, Marta Derecka, Pierre Cauchy, Thomas Manke, Berthold Göttgens, Rudolf Grosschedl

**Affiliations:** 1 Max Planck Institute of Immunobiology and Epigenetics, Freiburg, Germany; 2 International Max Planck Research School for Molecular and Cellular Biology, Max Planck Institute of Immunobiology and Epigenetics, Freiburg, Germany; 3 Faculty of Biology, University of Freiburg, Freiburg, Germany; 4 Wellcome-MRC Cambridge Stem Cell Institute, Department of Haematology, Jeffrey Cheah Biomedical Centre, University of Cambridge, Cambridge, UK

## Abstract

Hematopoietic stem cells (HSCs) and multipotent progenitors (MPPs) generate all cells of the blood system. Despite their multipotency, MPPs display poorly understood lineage bias. Here, we examine whether lineage-specifying transcription factors, such as the B-lineage determinant EBF1, regulate lineage preference in early progenitors. We detect low-level EBF1 expression in myeloid-biased MPP3 and lymphoid-biased MPP4 cells, coinciding with expression of the myeloid determinant C/EBPα. Hematopoietic deletion of *Ebf1* results in enhanced myelopoiesis and reduced HSC repopulation capacity. *Ebf1*-deficient MPP3 and MPP4 cells exhibit an augmented myeloid differentiation potential and a transcriptome with an enriched C/EBPα signature. Correspondingly, EBF1 binds the *Cebpa* enhancer, and the deficiency and overexpression of *Ebf1* in MPP3 and MPP4 cells lead to an up- and downregulation of *Cebpa* expression, respectively. In addition, EBF1 primes the chromatin of B-lymphoid enhancers specifically in MPP3 cells. Thus, our study implicates EBF1 in regulating myeloid/lymphoid fate bias in MPPs by constraining C/EBPα-driven myelopoiesis and priming the B-lymphoid fate.

## Teaser

B lineage transcription factor EBF1 is expressed at low levels in MPP3 and MPP4 cells. EBF1 primes the chromatin of B-lymphoid enhancers in MPP3 cells, and hematopoietic *Ebf1* deletion results in augmented C/EBPα-target gene signature and enhanced myeloid differentiation.

## Graphical Abstract

**Figure:**
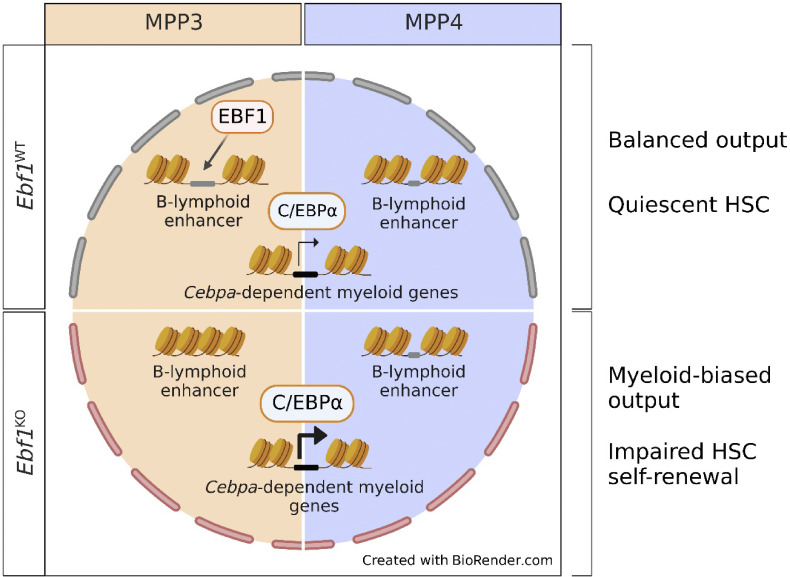

## Introduction

Hematopoietic stem and progenitor cell (HSPC) differentiation is regulated by an interplay of cell-extrinsic and -intrinsic cues. Hematopoietic stem cells (HSCs) are located at the apex of this process and are characterized by their quiescence and capacity for multi-lineage reconstitution of the entire hematopoietic system (Domen and Weissman, 1999; Orkin and Zon, 2008). HSCs can give rise to multipotent progenitors (MPPs) that are further defined by their lineage potentials. MPP2 differentiates preferentially toward erythroid and megakaryocytic lineages, MPP3 toward myeloid lineages, and MPP4 toward lymphoid lineages (Cabezas-Wallscheid et al., 2014; Pietras et al., 2015; Rodriguez-Fraticelli et al., 2018; Weinreb et al., 2020; Sommerkamp et al., 2021). Myeloid-restricted pre-granulocyte-macrophage (pre-GM) progenitors and, further, restricted granulocyte/monocyte progenitors (GMPs) represent the continuum of myeloid differentiation (Pronk et al., 2007; Drissen et al., 2016). Common lymphoid progenitors (CLPs), which include all-lymphoid progenitors (ALPs) and B-lymphoid progenitors (BLPs), represent the continuum of early lymphoid differentiation (Inlay et al., 2009; Jensen et al., 2018). In recent years, the hierarchical and stepwise differentiation model of hematopoiesis has been challenged by a model of gradual restriction of lineage potential. Other than the central role of lineage-specifying transcription factors (LS-TFs), the restriction of lineage potential is accompanied by gradual changes in the chromatin landscape and repression of lineage genes (Hu et al., 1997; Miyamoto et al., 2002; Nimmo et al., 2015; Paul et al., 2015; Palii et al., 2019; Ranzoni et al., 2021). Moreover, we have come to appreciate the heterogeneity of the HSPC compartment and the plasticity of lineage-biased progenitors that exist along a spectrum of lineage specification rather than as discrete bi-potent populations.

Enhancers associated with lineage-specific genes can be primed in HSPCs before their gene expression in committed cells (Heinz et al., 2010; Mercer et al., 2011; Lara-Astiaso et al., 2014). Furthermore, LS-TFs can prime the chromatin landscape and/or initiate lineage-specific gene regulatory networks, thus directing the path of differentiation. This function of LS-TFs is shown by their ability to impose specific cell fates upon forced expression. For example, EBF1 overexpression in LSKs restricts lymphopoiesis to a B-lymphoid output (Zhang et al., 2003), whereas C/EBPα overexpression in fibroblastic cells or B cell progenitors results in transdifferentiation to granulocytic–monocytic cell types (Xie et al., 2004; Fukuchi et al., 2006; Feng et al., 2008; Bussmann et al., 2009; Di Tullio et al., 2011). In MPPs, the relative expression levels of LS-TFs can determine the resulting cell fate. High levels of PU.1 and low C/EBPα expression are inversely instructive of monocyte versus granulocyte cell fate, whereas low levels of PU.1, achieved through transcriptional repression by Ikaros and Gfi1, are required for B cell specification (DeKoter and Singh, 2000; Dahl et al., 2007; Spooner et al., 2009; Zarnegar and Rothenberg, 2012). In addition to their role in the differentiation of intermediate progenitors, PU.1 and C/EBPα are also required to maintain HSC quiescence and self-renewal capacity (Iwasaki et al., 2005; Ye et al., 2013; Hasemann et al., 2014; Chavez et al., 2021).

EBF1 is at the core of the B cell specification and commitment program, as it is sufficient to rescue the developmental block of PU.1, Ikaros, or E2A deficiency in cultured progenitors (Pongubala et al., 2008; Reynaud et al., 2008). In addition to its role in activating the B cell–specific gene program and suppression of alternative lineage programs in collaboration with Pax5 (Lin and Grosschedl, 1995; Mikkola et al., 2002; Medina et al., 2004; Cobaleda et al., 2007; Treiber et al., 2010; Györy et al., 2012; Nechanitzky et al., 2013; Boller et al., 2016), recent studies have shown that EBF1 can regulate the chromatin landscape by binding to closed chromatin regions and opening these regions via the recruitment of remodeling complexes (Boller et al., 2016; Li et al., 2018; Wang et al., 2020). Furthermore, a knockdown of *EBF1* in human cord blood cells was found to result in an increased frequency of phenotypic HSCs, raising the possibility of additional functions of EBF1 in early hematopoiesis (van Galen et al., 2014).

Here, we find that hematopoietic *Ebf1* deletion results in a myeloid-biased HSPC output and decreased HSC quiescence and repopulation capacity. Furthermore, we show that EBF1 confers accessibility upon B-lymphoid enhancers at naive chromatin sites, specifically in myeloid-biased MPP3 progenitors. Additionally, we show that *Ebf1* deficiency in MPP3 and MPP4 cells is associated with impaired EBF1-dependent repression of myeloid-determinant C/EBPα, leading to an enhanced myeloid differentiation potential of *Ebf1*-deficient MPPs.

## Results

### EBF1 is expressed at low levels in MPP3 and MPP4 cells

**Figure 1.**
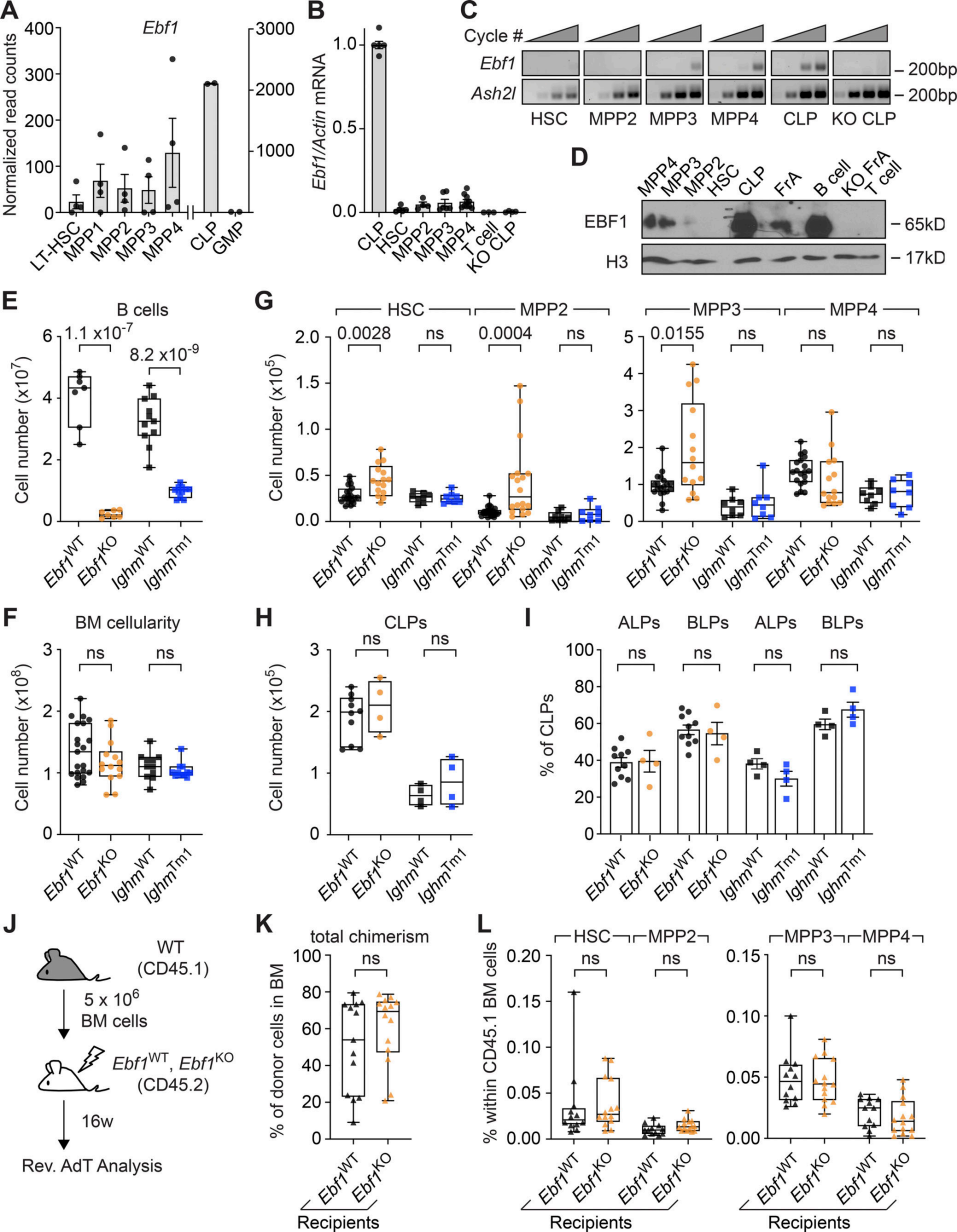
**EBF1 expression in MPP3 and MPP4 populations are required for normal HSPC composition. (A)** Normalized read counts of *Ebf1* in different hematopoietic cell populations from public RNA-seq datasets (original data from Sommerkamp et al. (2020) [left] and GEO dataset GSE162662 [right]). Data from GEO dataset GSE162662 representing CLP and GMP populations are represented on a different axis scale than data from Sommerkamp et al. (2020) representing LT-HSC, MPP1, MPP2, MPP3, and MPP4 populations. **(B)** qRT-PCR of *Ebf1* mRNA expression relative to *Actb*, normalized to WT *Ebf1* CLP (Lin^−^Sca1^int^cKit^int^IL7R^+^Flt3^+^) expression. Data are represented as mean ± SEM. *n* = 3–10. **(C)** Relative expression of *Ebf1* and *Ash2l* by RT-PCR. The first outer PCR was performed with 10 cycles and the second PCR was performed with increasing cycle numbers (25, 30, 35, and 40 cycles). The product of the second PCR was visualized on an agarose gel. Image is representative of three experiments. **(B and C)** cDNA was generated from 10,000 FACS sorted cells of the indicated populations. **(D)** Immunoblot analysis of EBF1 protein levels in total cell extracts of 150,000 FACS sorted cells of HSC (LSK CD150^+^CD48^−^), MPP2 (LSK CD150^+^CD48^+^), MPP3 (LSK CD150^−^CD48^+^), MPP4 (LSK CD150^−^CD48^+^Flt3^+^), CLPs (Lin^−^cKit^int^Sca1^int^IL7R^+^Flt3^+^), splenic B (CD19^+^) cells, and Fr. A (pre-pro-B) cells from WT *Ebf1* mice. T (CD4^+^CD8^+^) cells and *Ebf1*^KO^ Fr. A cells were used as a negative control. Histone H3 was used as a loading control. Immunoblot is representative of three experiments. **(E)** Absolute number of CD19^+^ B cells in the BM. *Ebf1*^WT^ and *Ebf1*^KO^ *n* = 7, *Ighm*^WT^ and *Ighm*^Tm1^ *n* = 11. **(F)** Total BM cellularity. *Ebf1*^WT^ *n* = 24, *Ebf1*^KO^ *n* = 14, *Ighm*^WT^ and *Ighm*^Tm1^ *n* = 11. **(G)** Boxplots showing the absolute number of HSC, erythroid-platelet biased MPP2, myeloid-biased MPP3, and lymphoid-biased MPP4 populations in the BM. *Ebf1*^WT^ *n* = 19–23, *Ebf1*^KO^ *n* = 14–18, *Ighm*^WT^ and *Ighm*^Tm1^ *n* = 8. **(H)** Absolute number of CLP cells in the BM. **(I)** Proportion of ALP and BLP cells in the CLP population. **(H and I)** *Ebf1*^WT^ *n* = 10, *Ebf1*^KO^ *n* = 4, *Ighm*^WT^ and *Ighm*^Tm1^ *n* = 4. **(J)** Schematic of reverse AdT assays with *Ebf1*^WT^ and *Ebf1*^KO^ mice. 5 × 10^6^ total BM cells of WT mice (CD45.1) were injected into lethally irradiated *Ebf1*^WT^ and *Ebf1*^KO^ recipients (CD45.2). **(K)** Boxplots showing the frequency of CD45.1 donor cells in the BM of *Ebf1*^WT^ and *Ebf1*^KO^ recipients, 16 wk after AdT. **(L)** Boxplots showing the frequencies of HSCs, MPP2, MPP3, and MPP4 within CD45.1 donor cells in the BM of the *Ebf1*^WT^ and *Ebf1*^KO^ recipients 16 wk after AdT. **(K and L)** *Ebf1*^WT^ *n* = 13, *Ebf1*^KO^ *n* = 14. **(E–L)** Statistical significance was determined by Mann–Whitney *U* test. **(B–L)** Data are from >2 independent experiments. Source data are available for this figure: SourceData F1.

**Figure S1.**
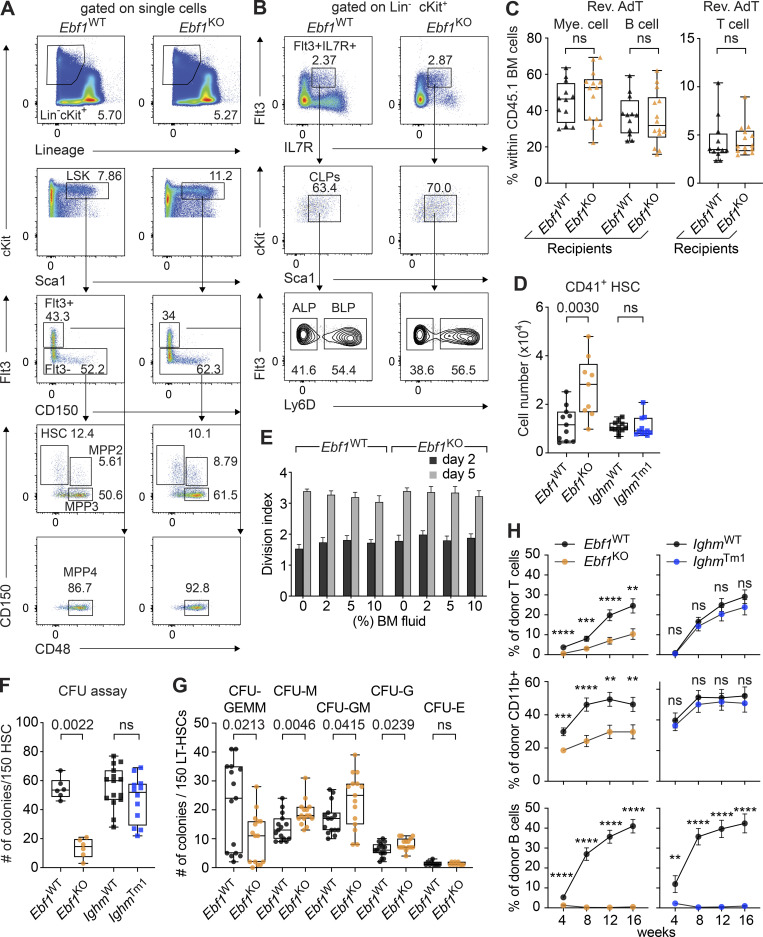
**Extended characterization of HSC functionality upon *Ebf1* deficiency.** Related to Figs. 1 and 2. **(A)** Gating strategies for HSC, MPP2, MPP3, and MPP4 cells. **(B)** Gating strategies for CLP, ALP, and BLP cells. Representative plots are shown for *Ebf1*^WT^ and *Ebf1*^KO^ mice. **(C)** Frequencies of myeloid cells (CD11b^+^), B cells (CD19^+^), and T cells (CD4^+^ CD8^+^) within CD45.1 donor cells in the BM of the *Ebf1*^WT^ and *Ebf1*^KO^ recipients 16 wk after AdT. *Ebf1*^WT^ *n* = 13, *Ebf1*^KO^ *n* = 14. **(D)** Absolute number of CD41^+^ HSCs in the BM. *Ebf1*^WT^ *n* = 11, *Ebf1*^KO^ *n* = 9, *Ighm*^WT^ *n* = 11, and *Ighm*^Tm1^ *n* = 14. **(E)** 300 WT CTY-labeled HSCs were cultured in TPO and SCF, with increased concentrations of BM fluid collected from *Ebf1*^WT^ and *Ebf1*^KO^ mice. Division index (CTY mean fluorescence intensity [MFI] from day n/CTY MFI from day 0) at day 2 and day 5, of CTY-labeled HSCs cultured with different BM fluid concentrations from *Ebf1*^WT^ and *Ebf1*^KO^ mice, *n* = 6–8. **(F)** CFU assay performed with 150 sorted HSCs plated in triplicate in methocult. Total number of colonies formed after 10–12 d of plating. Biological replicates *Ebf1*^WT^ and *Ebf1*^KO^ *n* = 2, *Ighm*^WT^ and *Ighm*^Tm1^ *n* = 3. **(G)** CFU assay performed with 150 sorted LT-HSCs (LSK CD34^−^Flt3^−^CD150^+^CD48^−^) plated in triplicate in methocult. Colony identification after 10–12 d of plating. Biological replicates *Ebf1*^WT^ and *Ebf1*^KO^ *n* = 5. **(H)** Frequency of CD45.2 donor-derived cells in the peripheral blood within T cells (CD4^+^ CD8^+^; ****, P < 0.0001; ***, P = 0.0004; **, P = 0.0013), myeloid cells (CD11b^+^; ***, P = 0.0002; ****, P < 0.0001; **, P = 0.0010; **, P = 0.0024) and B cells (CD19^+^; **, P = 0.0060; ****, P < 0.0001), during the AdT. Data are represented as mean ± SEM. *Ebf1*^WT^ *n* = 33 and *Ebf1*^KO^ *n* = 40, *Ighm*^WT^ *n* = 17, and *Ighm*^Tm1^ *n* = 19. **(C–H)** Statistical significance was determined by Mann-Whitney *U* test. Data are from >2 independent experiments.

To examine whether EBF1 could play a role in HSPCs, we first assessed *Ebf1* expression in publicly available bulk RNA-sequencing (RNA-seq) datasets of the following cell populations: LT-HSC (Lin^−^Sca1^+^cKit^+^ [LSK] CD34^−^Flt3^−^CD150^+^CD48^−^), MPP1 (LSK CD34^+^Flt3^−^CD150^+^CD48^−^), MPP2 (LSK CD34^+^Flt3^−^CD150^+^CD48^+^), MPP3 (LSK CD34^+^Flt3^−^CD150^−^CD48^+^), MPP4 (LSK CD34^+^Flt3^+^CD150^−^CD48^+^), CLP (Lin^−^Sca1^int^cKit^int^IL7R^+^Flt3^+^), and GMP (Lin^−^Sca1^−^cKit^+^CD41^−^CD16/32^+^; Sommerkamp et al., 2020, GEO accession no. GSE162607). As expected, *Ebf1* mRNA was detected at high level in the CLPs and was absent in the GMPs (Fig. 1 A). Interestingly, *Ebf1* transcripts were detected in multiple HSPC populations (defined by the LSK immunophenotype), albeit at much lower levels than in CLPs. To confirm the expression of *Ebf1* in HSPCs, we sorted different cell populations by flow cytometry (Fig. S1, A and B) and analyzed their RNA for the presence of *Ebf1* transcripts by quantitative reverse transcriptase (qRT)-PCR and by nested RT-PCR. Consistent with bulk RNA-seq results, qRT-PCR analysis of *Ebf1* expression, normalized to expression in CLPs, showed low but consistent *Ebf1* levels in MPP2, MPP3, and MPP4 cells and no expression in T cells and *Ebf1*^fl/fl^ *Tie2*^Cre^ (KO) CLPs (Fig. 1 B). Further, nested RT-PCR analysis showed that *Ebf1* expression was detectable only in MPP3 and MPP4 cells, albeit at lower levels than in CLPs (Fig. 1 C). In contrast to the qRT-PCR and bulk RNA-seq analysis, *Ebf1* transcripts were not detected in MPP2 cells (Fig. 1 C). We also assessed EBF1 protein expression in total cell lysates of 150,000 FACS-sorted HSPCs by immunoblot (Fig. 1 D). EBF1 expression was detected in MPP3 and MPP4 cells, albeit at much lower levels than in CLP and splenic CD19^+^ B cells. No EBF1 expression was detected in *Ebf1*^fl/fl^ *Tie2*^Cre^ (KO) pre-pro-B (Fr. A) cells, T cells, and HSCs (LSK Flt3^−^CD150^+^CD48^−^), and was hardly detectable in MPP2 cells (Fig. 1 D). Together, these data suggest that EBF1 is expressed at low but detectable levels in MPP3 and MPP4 cells.

### *Ebf1* deficiency alters the composition of the HSPC compartment

To elucidate the role of EBF1 in HSPCs, we generated *Ebf1*^wt/wt^ *Tie2*^Cre^ *and Ebf1*^fl/fl^ *Tie2*^Cre^ mice, hereafter called *Ebf1*^WT^ and *Ebf1*^KO^ mice. *Tie2*^*+/Cre*^ drives Cre expression in the endothelium and adult HSCs (Jaffredo et al., 1998; Kisanuki et al., 2001; Zovein et al., 2008). To disentangle the effects of *Ebf1* deletion in HSPCs from the effects of B cell deficiency, we analyzed B cell–deficient *Ighm*^Tm1^ mice, also known as muMT^−^ mice, which lack the expression of membrane-bound IgM (Kitamura et al., 1991). The efficiency of *Ebf1* and *Ighm* mutations was confirmed by the absence of CD19^+^ B cells in the bone marrow (BM; Fig. 1 E). To assess the BM composition, we compared the absolute number of cells within the HSPC compartment. Importantly, no significant differences were observed in the total BM cellularity of *Ebf1*^KO^ and *Ighm*^Tm1^ mice compared to *Ebf1*^WT^ and *Ighm*^WT^ mice (Fig. 1 F). Flow cytometric analysis showed a significant increase in absolute numbers of HSCs, MPP2, and MPP3 cells in the BM of *Ebf1*^KO^ mice relative to *Ebf1*^WT^ mice, while no significant differences were observed in *Ighm*^Tm1^ mice (Fig. 1 G). The absolute number of lymphoid-biased MPP4 cells and CLPs showed no significant changes in the BM of *Ebf1*^KO^ and *Ighm*^Tm1^ mice compared to *Ebf1*^WT^ mice and *Ighm*^WT^ mice, respectively (Fig. 1, G and H). CLPs contain ALPs (Lin^−^Sca1^int^cKit^int^IL7R^+^Flt3^+^Ly6D^−^) that give rise to B, T, and natural killer cells, as well as BLPs (Lin^−^Sca1^int^cKit^int^IL7R^+^Flt3^+^Ly6D^+^) that generate B cells (Inlay et al., 2009; Jensen et al., 2018). Therefore, we also examined their relative frequencies in *Ebf1*^KO^ and *Ighm*^Tm1^ mice and found no significant changes relative to the *Ebf1*^WT^ and *Ighm*^WT^ counterparts, indicating that B cell deficiency does not lead to an accumulation of BLPs (Fig. 1 I).

Mesenchymal stromal cell–specific *Ebf1* deletion elicits long-term changes in HSC function (Derecka et al., 2020), and EBF1 is expressed in endothelial cells. To examine whether or not a *Tie2*^Cre^-mediated deletion of *Ebf1* in endothelial cells may account for the observed changes in HSPC composition, we performed reverse adoptive transfer (AdT) assays in which lethally irradiated CD45.2 *Ebf1*^WT^ and *Ebf1*^KO^ recipients were reconstituted with 5 × 10^6^ BM cells from CD45.1 WT mice (Fig. 1 J). We observed a similar overall frequency of WT donor cells in the BM of *Ebf1*^WT^ and *Ebf1*^KO^ recipients (Fig. 1 K). Moreover, the frequencies of WT HSCs, MPP2, MPP3, and MPP4 cells, as well as mature B, T, and myeloid cells were similar in *Ebf1*^WT^ and *Ebf1*^KO^ recipients (Fig. 1 L and Fig. S1 C). Together, these data show that the *Tie2*^Cre^-mediated deletion of *Ebf1* in the hematopoietic compartment leads to an increase in the numbers of HSCs, MPP2, and myeloid-biased MPP3 cells, while leaving the lymphoid-biased MPP4 cells and CLPs numbers unaffected. Importantly, these effects were neither due to a deletion of *Ebf1* in the endothelium of the BM niche nor caused by the lack of B cells, confirming an HSPC-specific role of EBF1.

### *Ebf1* deficiency leads to reduced HSC quiescence and self-renewal capacity

**Figure 2.**
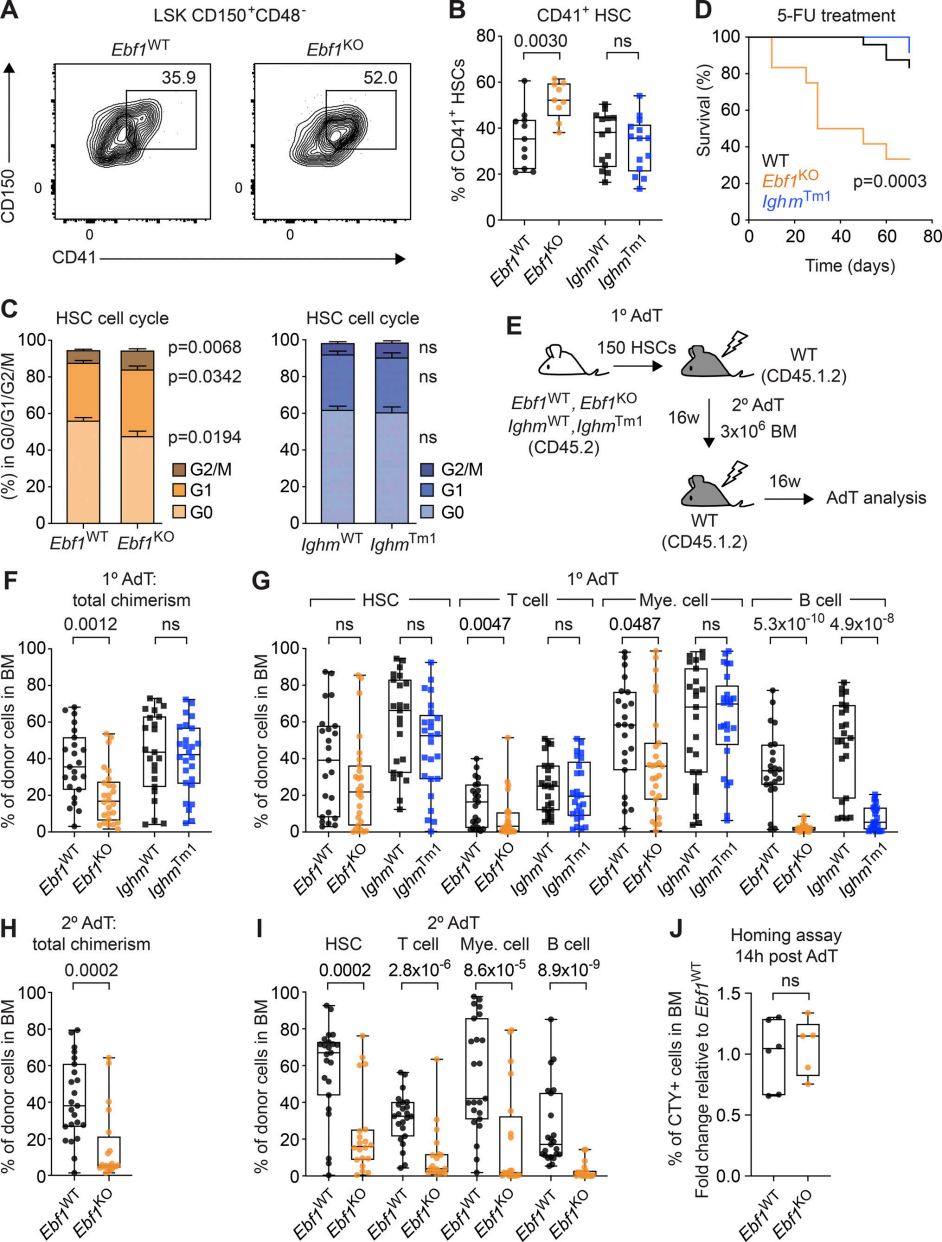
**Reduced HSC quiescence and self-renewal capacity upon *Ebf1* deletion. (A)** Representative contour plots showing CD41^+^ expression on HSCs in *Ebf1*^WT^ and *Ebf1*^KO^ mice. **(B)** Boxplots showing the percentage of CD41^+^ expressing cells in the HSC population. *Ebf1*^WT^ *n* = 11, *Ebf1*^KO^ *n* = 9, *Ighm*^WT^ and *Ighm*^Tm1^ *n* = 11. **(C)** Percentage of HSCs within each cell cycle phase (G0, G1, G2/S/M). *Ebf1*^WT^ *n* = 16, *Ebf1*^KO^ *n* = 16, *Ighm*^WT^ and *Ighm*^Tm1^ *n* = 11. Data are represented as mean ± SEM. **(D)** Kaplan–Meier survival curve of WT, *Ighm*^Tm1^, and *Ebf1*^KO^ mice following 5-FU injections every 10 d. *Ebf1*^WT^ and *Ighm*^WT^ mice are represented together as WT mice. *Ebf1*^WT^ *n* = 14, *Ebf1*^KO^ *n* = 12, *Ighm*^WT^ *n* = 10 and *Ighm*^Tm1^ *n* = 12. Statistical analysis was performed using the log-rank (Mantel-Cox) test. **(E)** Schematic showing primary AdT assays with *Ebf1*^WT^, *Ebf1*^KO^, *Ighm*^WT^, and *Ighm*^Tm1^ mice and secondary AdT assays with *Ebf1*^WT^ and *Ebf1*^KO^ mice. Lethally irradiated primary WT recipients (CD45.1.2) were injected with 150 HSCs from the indicated donors (CD45.2), together with 5 × 10^5^ supportive (CD45.1) BM cells. Lethally irradiated secondary WT recipients (CD45.1.2) were injected with 3 × 10^6^ total BM cells from the indicated primary recipient donors. **(F)** Boxplots showing frequency of CD45.2 donor cells in the BM of primary recipients, 16 wk after AdT. **(G)** Boxplots showing the frequencies of donor-derived cells within HSCs, T cells (CD4^+^CD8^+^), B cells (CD19^+^), and myeloid cells (CD11b^+^) in the BM of the primary recipients 16 wk after AdT. **(F and G)** *Ebf1*^WT^ *n* = 23, *Ebf1*^KO^ *n* = 25, *Ighm*^WT^ *n* = 23 and *Ighm*^Tm1^ *n* = 26. **(H)** Boxplots showing frequency of CD45.2 donor cells in the BM of secondary recipients, 16 wk after AdT. **(I)** Boxplots showing the frequencies of donor-derived cells within HSCs, T cells, B cells, and myeloid cells in the BM of the secondary recipients 16 wk after AdT. **(H and I)** *Ebf1*^WT^ *n* = 23, *Ebf1*^KO^ *n* = 20. **(J)** 50,000 CTY-labeled LSK cells from *Ebf1*^WT^ and *Ebf1*^KO^ mice (CD45.2) were injected into lethally irradiated WT recipients (CD45.1). Frequency of CTY positive cells in the BM 14 h after AdT, fold change relative to *Ebf1*^WT^. *Ebf1*^WT^ *n* = 6, *Ebf1*^KO^ *n* = 5. **(B, C, and F–J)** Statistical significance was determined by Mann–Whitney *U* test. **(A–J)** Data are from >2 independent experiments. HSCs defined as LSK CD150^+^CD48^−^.

Given that HSCs are at the apex of hematopoiesis, we first explored the role of EBF1 for HSCs by extending our flow cytometric analysis. *Ebf1*^KO^ HSCs showed increased expression of CD41 and increased absolute numbers of CD41^+^ HSCs in *Ebf1*^KO^ mice relative to *Ebf1*^WT^ mice (Fig. 2, A and B; and Fig. S1 D). CD41 expression on HSCs is indicative of a myeloid-biased output (Gekas and Graf, 2013; Yamamoto et al., 2013; Miyawaki et al., 2015) and active HSCs (Wilson et al., 2015). Therefore, we assessed the HSC cell cycle status and detected a significantly lower percentage of *Ebf1*^KO^ HSCs in G0 phase and a significantly higher percentage of *Ebf1*^KO^ HSCs in G1 and S/G2/M phase as compared to *Ebf1*^WT^ HSCs (Fig. 2 C). In contrast, *Ighm*^Tm1^ HSCs showed no increase in CD41 expression, and no difference in the cell cycle status relative to *Ighm*^WT^ HSCs (Fig. 2, A–C; and Fig. S1 D). We also examined the activation status of HSCs under proliferative stress through chronic 5-fluorouracil (5-FU) treatment. In line with the increased cycling of *Ebf1*^KO^ HSCs, the median survival of 5-FU–treated *Ebf1*^KO^ mice was lower than that of 5-FU–treated *Ighm*^Tm1^ and WT (*Ebf1*^WT^ and *Ighm*^WT^) mice (Fig. 2 D). Together, these results indicate that *Ebf1*^KO^ mice have HSCs that are less quiescent and exhaust faster.

MPP3 cells can secrete HSC-activating cytokines (Kang et al., 2021) and, therefore, we examined whether the increase in MPP3 numbers in *Ebf1*^KO^ BM could contribute to an HSC-activating cytokine environment. To this end, we cultured Cell Trace Yellow (CTY)–labeled WT HSCs in media containing increasing concentrations of *Ebf1*^WT^ or *Ebf1*^KO^ BM fluid and evaluated CTY dye dilution as an index of HSC proliferation. The proliferation index of HSCs was unchanged regardless of the BM fluid conditions, suggesting that the increased proliferation of *Ebf1*^KO^ HSCs is not simply attributable to changes in the soluble cytokine environment of *Ebf1*^KO^ BM (Fig. S1 E).

Given that quiescence correlates with repopulation potential (Passegué et al., 2005; Foudi et al., 2009; Lauridsen et al., 2018), we also tested whether *Ebf1*^*KO*^ HSCs were functionally impaired. In CFU assays, *Ebf1*^KO^, but not *Ighm*^Tm1^, HSCs produced significantly fewer colonies relative to WT HSCs (Fig. S1 F). When assessing the type of colonies generated by *Ebf1*^KO^ HSCs, we find a significant increase in uni- and bi-lineage granulocyte and monocyte colonies, and a significant decrease in multipotent CFU-GEMM colonies relative to *Ebf1*^WT^ HSCs (Fig. S1 G). These data suggest an impaired self-renewal capacity relative to *Ebf1*^WT^ HSCs. To assess their self-renewal capacity in vivo, we transferred 150 FACS-sorted HSCs from *Ebf1*^WT^, *Ebf1*^KO^, *Ighm*^WT^, and *Ighm*^Tm1^ mice into primary WT recipients and analyzed their BM chimerism after 16 wk (Fig. 2 E). The total donor contribution in the BM of primary recipients was significantly lower for transplanted *Ebf1*^KO^ HSCs than for *Ebf1*^WT^ HSCs, whereas the total donor contribution was similar for *Ighm*^WT^ and *Ighm*^Tm1^ HSCs (Fig. 2 F). The donor contribution of *Ebf1*^KO^ HSCs, in the periphery throughout the AdT and in the BM, to T cells and myeloid cells was also reduced, whereas the contribution to HSCs was not significantly changed (Fig. 2 G and Fig. S1 H). As expected, B cell reconstitution was absent for recipients of *Ebf1*^KO^ and *Ighm*^Tm1^ HSCs (Fig. 2 G and Fig. S1 H). Secondary AdT assays, performed with 3 × 10^6^ total BM cells from *Ebf1*^WT^ and *Ebf1*^KO^ primary recipients, showed a further reduction in BM donor chimerism and an enhanced multilineage reconstitution defect (Fig. 2, H and I). To address whether *Ebf1*^KO^ HSCs have a decreased repopulating capacity due to a homing defect, we FACS-sorted 5 × 10^4^ CTY-labeled LSKs from *Ebf1*^WT^ and *Ebf1*^KO^ mice as an estimate of HSC homing capacity, injected them into WT recipients, and evaluated the frequency CTY-positive cells in the BM of recipients 14 h after transfer. This analysis revealed that *Ebf1*^WT^ and *Ebf1*^KO^ hematopoietic progenitors home to the BM with similar efficiency (Fig. 2 J). Taken together, these data indicate that *Ebf1* deletion in HSPCs leads to an impaired HSC function. Given the lack of EBF1 expression in HSCs and MPP2 cells, the HSC phenotypes are likely indirect and secondary to the EBF1-driven changes in MPP3 and MPP4 cells.

### EBF1 confers de novo accessibility in MPP3 cells

**Figure S2.**
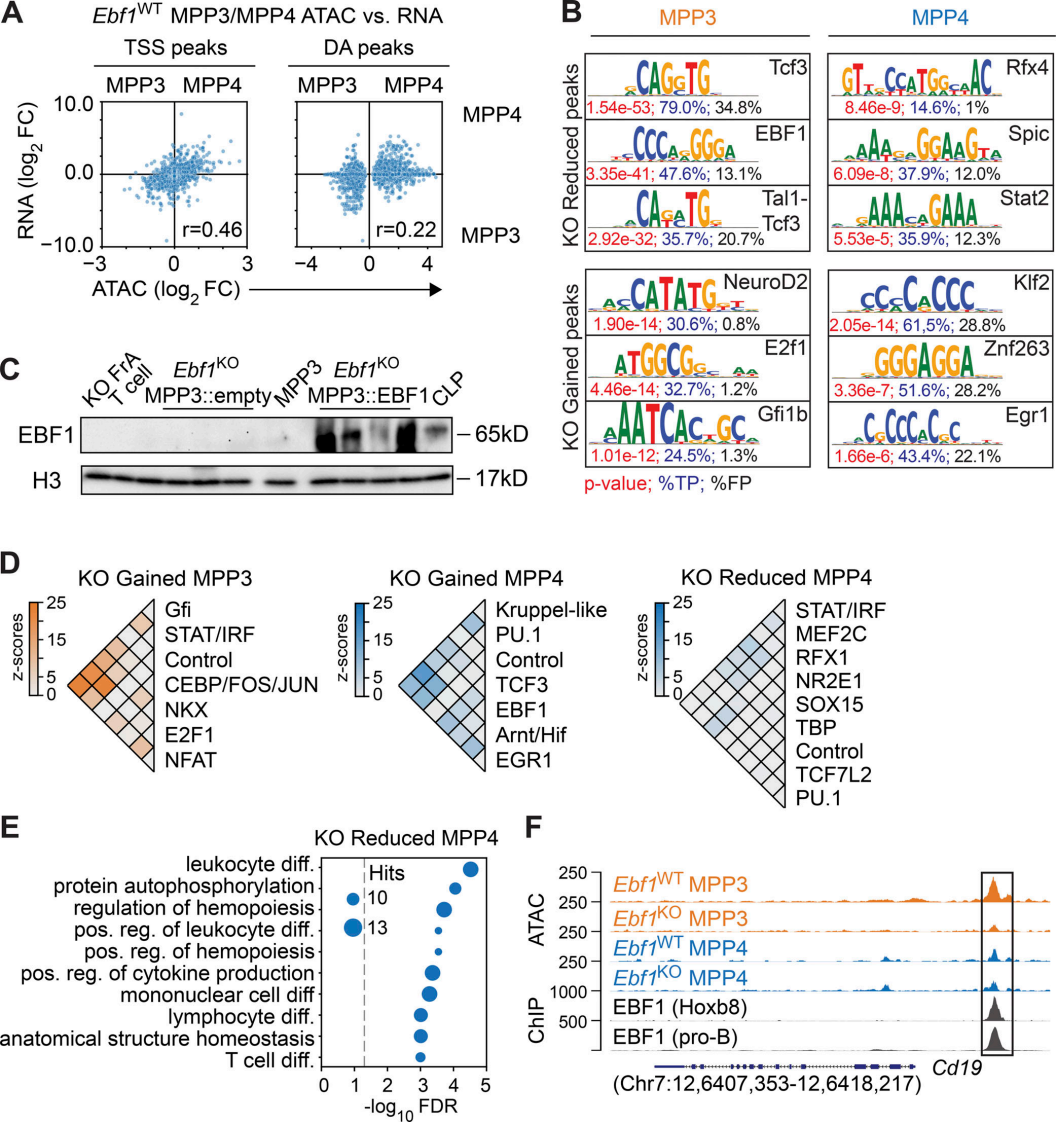
**Chromatin accessibility changes in Ebf1-deficient MPP3 and MPP4 cells.** Related to Fig. 3. **(A)** Comparison of chromatin accessibility changes to gene expression changes in MPP3 versus MPP4 cells, in *Ebf1*^WT^ conditions. The y axis represents log_2_ fold changes of gene expression, and the x axis represents log_2_ fold changes of peak read counts in MPP3 versus MPP4 cells in *Ebf1*^WT^ conditions. Left: Dots represent ATAC peaks that overlap their TSS (TSS peaks). Right: Dots represent DA peaks between MPP3 versus MPP4 cells in *Ebf1*^WT^ conditions. Biological replicates for RNA-seq *n* = 4. Biological replicates for ATAC-seq *n* = 2. **(B)** Sequence logos of top ranked enriched motifs underlying the DA peaks in MPP3 and MPP4 cells from *Ebf1*^WT^ and *Ebf1*^KO^ mice. P values are depicted in red, percentages in blue reflect the fraction of hits found in the peak set, percentages in black reflect the fraction of hits found in the background set. **(C)** Immunoblot analysis of EBF1 protein levels in total cell extracts of 150,000 FACS-sorted cells of lineage-negative GFP^+^ cells transduced with empty vector or EBF1 vector, biological replicates for ATAC-seq used in Fig. 3 D (*n* = 4). *Ebf1*^WT^ MPP3 cells and CLPs (Lin^−^cKit^int^Sca1^int^IL7R^+^Flt3^+^) were used a positive control. T (CD4^+^CD8^+^) cells and *Ebf1*^KO^ Fr. A cells were used as a negative control. Histone H3 was used as a loading control. **(D)** Heatmaps of co-occurrence counts in KO Gained MPP3 peaks and KO Reduced and KO Gained MPP4 peaks of enriched motifs in these DA peak sets versus co-occurrence counts in all MPP3 and MPP4 peaks (1,000 replicates), respectively. Co-occurrence counts with *z*-scores >5 are displayed. **(E)** Enrichment analysis of genes associated with KO Reduced MPP4 peaks. **(F)** Genome tracks showing ATAC signal and EBF1 ChIP signal at B-lymphoid related KO Reduced MPP3 peaks annotated to the *Cd19* gene. Original data from Li et al. (2018); Kucinski et al. (2020). Source data are available for this figure: SourceData FS2.

**Figure 3.**
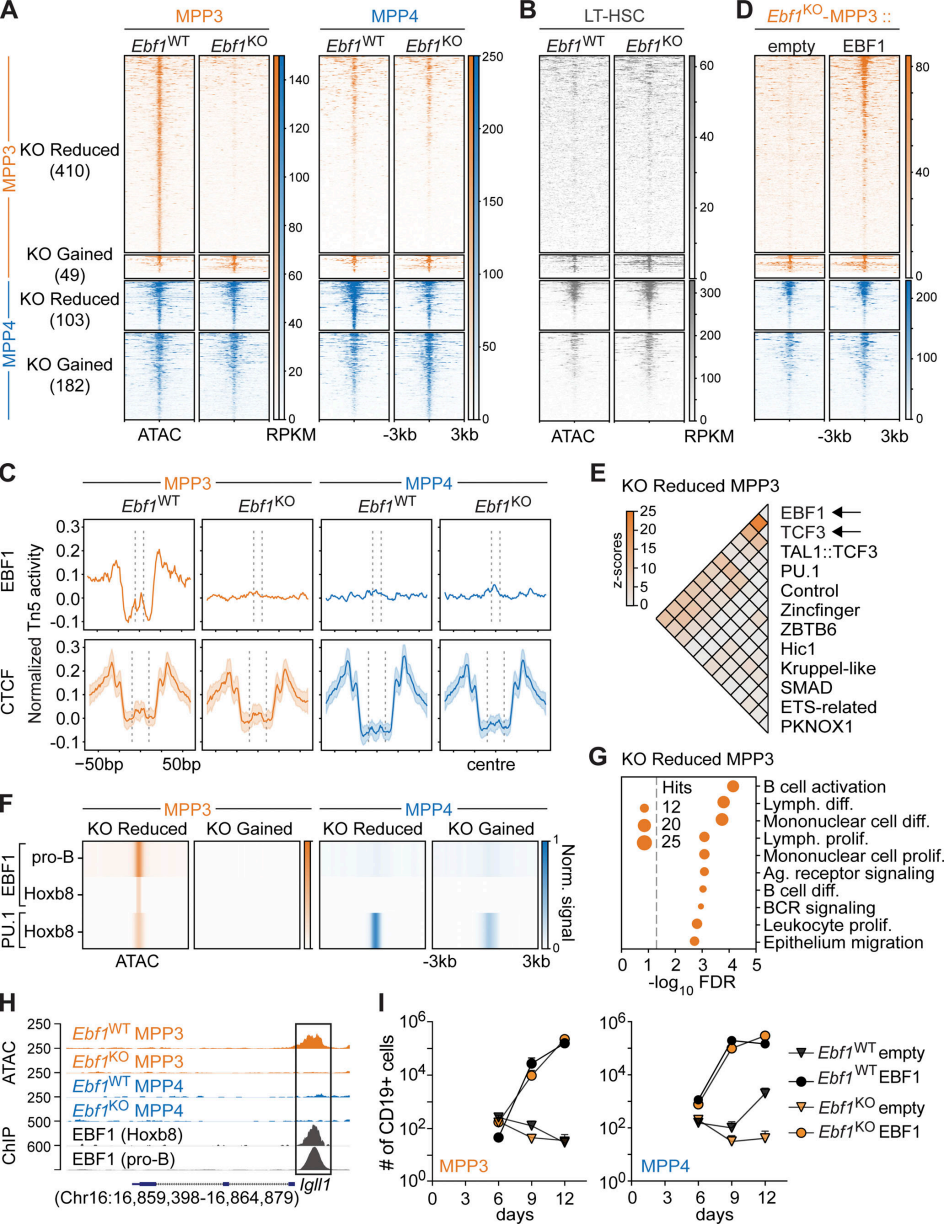
**EBF1 primes the B-lymphoid fate in myeloid-biased MPP3 progenitors. (A and B)** Heatmap displaying chromatin accessibility at DA peaks in MPP3 and MPP4 cells (A) and in LT-HSCs (LSK CD34^−^Flt3^−^CD150^+^CD48^−^; B) from *Ebf1*^WT^ and *Ebf1*^KO^ mice. Biological replicates *n* = 2. **(A, B, and D)** Peaks are organized into KO Reduced and KO Gained peaks, in MPP3 cells and MPP4 cells. Regions ± 3 kb around the center of the peak are shown. Heatmap scale represents RPKM. **(C)** Aggregation plots showing Tn5 activity in MPP3 and MPP4 cells from *Ebf1*^WT^ and *Ebf1*^KO^ conditions, at KO Reduced MPP3 peaks centered around the EBF1 motif (top row). Median aggregation plot for 100 random sets of CCCTC-binding factor (CTCF) motifs found in the ATAC peak set (bottom row). The shaded area depicts the SD. **(D)** Heatmap displaying chromatin accessibility at DA peaks in MPP3 cells from *Ebf1*^KO^ mice upon EBF1 re-expression and empty vector expression. **(E)** Heatmap displaying z-scores of co-occurrence counts in KO Reduced MPP3 peaks versus co-occurrence counts in all MPP3 peaks (1,000 replicates) of enriched motifs in KO Reduced MPP3 peaks. **(F)** Heatmap displaying average ChIP signal for EBF1 and PU.1 ChIPs at DA peaks. The RPKM signal is scaled over all four DA peak sets. **(G)** Enrichment analysis of genes associated with KO Reduced MPP3 peaks. **(H)** Genome tracks showing ATAC signal and EBF1 ChIP signal at B-lymphoid related KO Reduced MPP3 peaks annotated to the *Igll1* gene. **(I)** Absolute number of CD19^+^ B cells at indicated time points upon EBF1 re-expression and empty vector expression, in *Ebf1*^WT^ and *Ebf1*^KO^ MPP3 and MPP4 cells. Data are from >2 independent experiments. **(F and H)** Original data from Kucinski et al. (2020); Li et al. (2018).

During B-lymphoid differentiation, EBF1 activates lineage-specific transcriptional networks and induces chromatin landscape changes (Hagman et al., 2011; Boller et al., 2018; Li et al., 2018; Wang et al., 2020). Within the HSPC compartment, EBF1 expression was strongest in MPP3 and MPP4 cells (Fig. 1 D). To understand the molecular effects of *Ebf1* deletion in HSPCs, we thus analyzed the chromatin and transcriptome of MPP3 and MPP4 cells from *Ebf1*^*WT*^ and *Ebf1*^*KO*^ mice by bulk assay for transposase-accessible chromatin using sequencing (ATAC-seq) and RNA-seq, respectively. We observed a positive correlation between MPP3/MPP4 differentially accessible (DA) ATAC peaks overlapping with transcription start sites (TSS peaks) and the expression of MPP3/MPP4 differentially expressed (DE) genes (Fig. S2 A). This correlation shows that MPP3 and MPP4 chromatin accessibility profiles reflect the myeloid- and lymphoid-gene expression patterns of MPP3 and MPP4 cells, respectively (Cabezas-Wallscheid et al., 2014; Pietras et al., 2015; Rodriguez-Fraticelli et al., 2018; Weinreb et al., 2020; Sommerkamp et al., 2021). Next, we performed differential peak calling for *Ebf1*^WT^ versus *Ebf1*^KO^ MPP3 cells and *Ebf1*^WT^ versus *Ebf1*^KO^ MPP4 cells. The most pronounced effect of the *Ebf1* deletion was observed in MPP3 cells, in which 49 sites had gained and 410 sites had reduced accessibility in *Ebf1*^KO^ MPP3 cells relative to *Ebf1*^WT^ cells (Fig. 3 A and Table S2). Surprisingly, a minority of KO-reduced MPP3 peaks were detected in *Ebf1*^WT^ MPP4 cells (91 out of 410 peaks), but these peaks were not DA in *Ebf1*^WT^ versus *Ebf1*^KO^ MPP4 cells. A different set of sites showed modest changes in accessibility between *Ebf1*^WT^ and *Ebf1*^KO^ MPP4 cells (Fig. 3 A and Table S2). To assess whether the DA sites observed in MPP3 and MPP4 cells were accessible in upstream progenitors, we analyzed the chromatin landscape of *Ebf1*^WT^ and *Ebf1*^KO^ LT-HSCs. Notably, the sites that show reduced accessibility in *Ebf1*^KO^ MPP3 cells were not accessible in *Ebf1*^WT^ and *Ebf1*^KO^ LT-HSCs, whereas the other DA sites were detected in *Ebf1*^WT^ and *Ebf1*^KO^ LT-HSCs (Fig. 3 B). This result suggests that the sites of decreased accessibility in *Ebf1*^KO^ MPP3 cells are not accessible in stem cells.

Motif enrichment analysis of ATAC peaks that were reduced in *Ebf1*^KO^ MPP3 cells identified the EBF1- and TCF3 (E2A)-binding motifs as top-ranked hits, whereas other motifs, such as Rfx4, Spic, and Stat2, predominated the peaks reduced in *Ebf1*^KO^ MPP4 cells (Fig. S2 B). More than half of the peaks reduced in *Ebf1*^KO^ MPP3 cells (230 out of 410 peaks) contained the canonical EBF1 motif and digital footprinting, which determined the cumulative aggregation of Tn5 signals, revealed clear footprints of EBF1 occupancy in *Ebf1*^WT^ but not in *Ebf1*^KO^ MPP3 cells, and no footprints of EBF1 occupancy were detected in *Ebf1*^WT^ MPP4 cells (Fig. 3 C). To assess whether the limited number of sites provided sufficient depth for digital footprinting, we analyzed the Tn5 signal at predicted CTCF sites in random peaks with equal size, which showed similar CTCF occupancy in *Ebf1*^WT^ and *Ebf1*^KO^ MPP3 and MPP4 cells (Fig. 3 C). These data suggest a requirement for EBF1 occupancy at peaks reduced in *Ebf1*^KO^ MPP3 cells.

To assess whether forced EBF1 expression can restore accessibility at these sites, we re-expressed EBF1 in *Ebf1*^KO^ MPP3 cells by retroviral transduction (Fig. S2 C). We find that accessibility is gained specifically at peaks reduced in *Ebf1*^KO^ MPP3 cells in the EBF1 re-expressed cells, whereas the other DA sites are unchanged by EBF1 re-expression (Fig. 3 D). Together, these data show that EBF1 is necessary and sufficient for de novo accessibility to sites that show reduced accessibility in *Ebf1*^KO^ MPP3 cells.

### EBF1 primes B-lymphoid accessibility in MPP3 cells

To assess a possible co-regulation by transcription factors, we performed a co-occurrence analysis of enriched motifs in different DA peaks. Although PU.1 was an enriched motif in DA peaks when using an alternative enrichment analysis (see Materials and methods), we included PU.1 in the co-occurrence analysis given its crucial role in lymphoid and myeloid differentiation of MPPs (DeKoter and Singh, 2000; Pang et al., 2018). We detected co-occurrence of PU.1 motifs with enriched motifs in the different DA peaks, in line with its importance in hematopoietic progenitors (Fig. 3 E and Fig. S2 D). We observed the strongest co-occurrence between EBF1 and TCF3 (E2A) motifs at EBF1-dependent sites detected in MPP3 cells (Fig. 3 E), but not in the other DA peak sets (Fig. S2 D). This co-occurrence may represent functional cis-regulatory modules because EBF1 and E2A synergistically cooperate to establish B-lymphoid gene expression and they often bind chromatin in close proximity to each other (O’Riordan and Grosschedl, 1999; Lin et al., 2010).

To inspect EBF1 binding at DA peaks between *Ebf1*^WT^ and *Ebf1*^KO^ and MPP3 and MPP4 cells, we analyzed publicly available EBF1 chromatin immunoprecipitation (ChIP)-seq datasets in pro-B cells (Li et al., 2018) and in Hoxb8-FL cells (Kucinski et al., 2020). Hoxb8-FL cells are conditionally immortalized HSPCs that resemble MPPs by maintaining both lymphoid and myeloid potential (Redecke et al., 2013). We find that the EBF1-dependent peaks detected in MPP3 cells are bound by EBF1 in pro-B cells and in Hoxb8-FL cells (Fig. 3 F), suggesting that these EBF1-dependent sites are related to pro-B cells. Consistent with the digital footprinting analysis, no EBF1 binding was detected in *Ebf1*^WT^/*Ebf1*^KO^ DA peaks of MPP4 cells. Additionally, we analyzed the PU.1 ChIP-seq profile in Hoxb8-FL cells (Kucinski et al., 2020) and observed PU.1 binding at peaks reduced in *Ebf1*^KO^ MPP3 and MPP4 cells and at peaks gained in *Ebf1*^KO^ MPP4 cells (Fig. 3 F). This observation is in line with the importance of PU.1 in multipotent progenitors.

Gene ontology (GO) analysis of genes annotated to the peaks reduced in *Ebf1*^KO^ MPP4 cells exhibited enrichment for lymphoid-related molecular signatures (Fig. S2 E). Notably, GO analysis of the genes annotated to peaks reduced in *Ebf1*^KO^ MPP3 cells showed a specific enrichment for B-lymphoid molecular signatures, such as B cell receptor signaling and B cell differentiation (Fig. 3 G). These genes include B-lymphoid related genes, such as *Cd19* and *Igll1*, which show EBF1 occupancy in pro-B cells and Hoxb8-FL progenitors at sites accessible in *Ebf1*^WT^ MPP3 cells (Fig. 3 H and Fig. S2 F). Finally, we assessed whether EBF1 re-expression in *Ebf1*^KO^ MPP3 and MPP4 cells can rescue their B cell differentiation defect. We observed that re-expression of EBF1 in *Ebf1*^KO^ MPP3 and MPP4 cells allows their differentiation into CD19^+^ B cells (Fig. 3 I). Taken together, these data reveal a subset of B-lymphoid–associated regions that display an enhancer signature and EBF1-dependent accessibility in MPP3 cells.

### EBF1-dependent enhancers in MPP3 cells are associated with expression in CLPs

**Figure S3.**
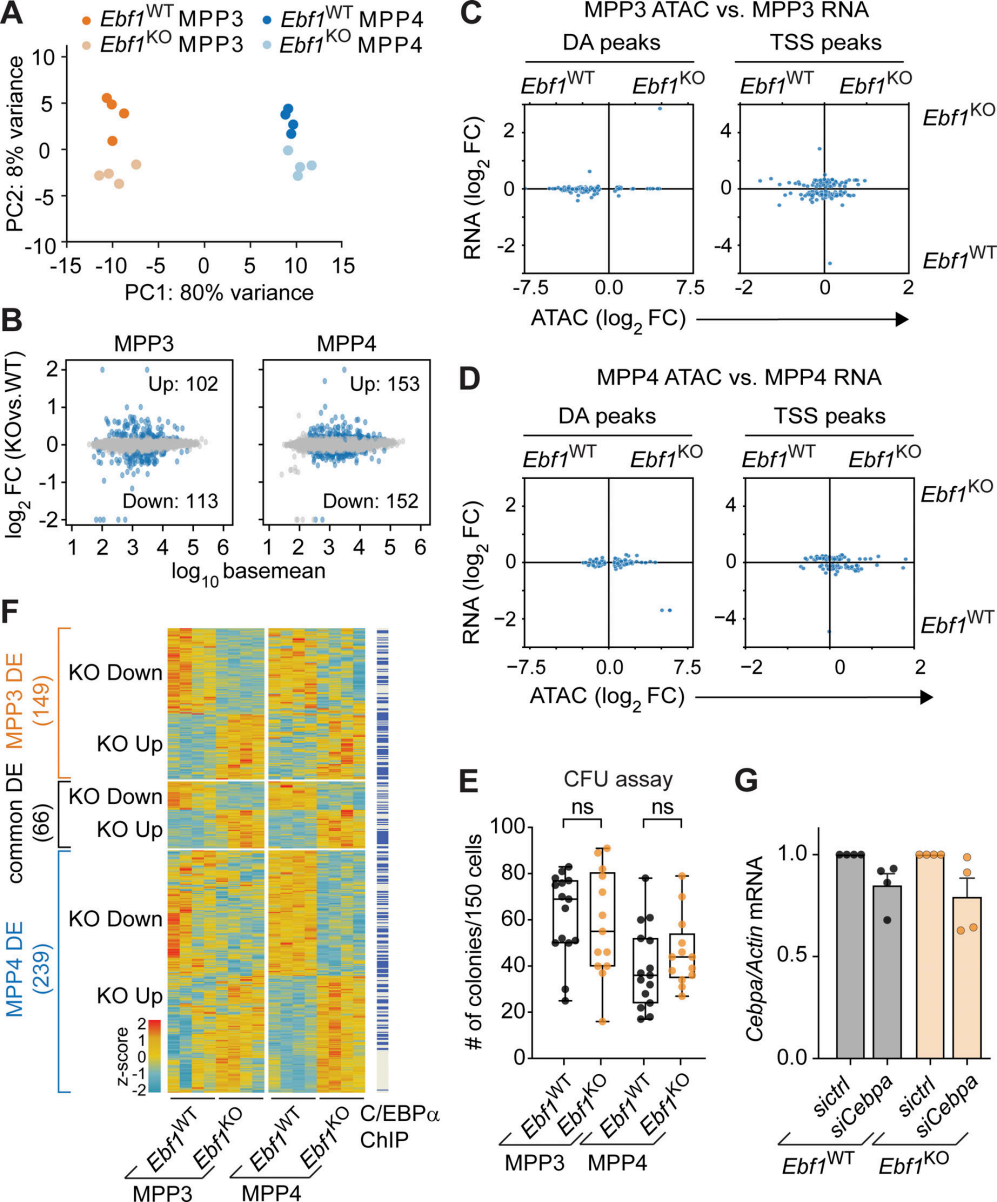
**Role of *Ebf1* deficiency in myeloid-biased transcriptome.** Related to Figs. 4 and 5. **(A)** PCA of bulk RNA-seq data from MPP3 and MPP4 cells of *Ebf1*^WT^ and *Ebf1*^KO^ conditions; based on DE genes. *n* = 4 biological replicates. **(B)** MA plots of MPP3 and MPP4 cells comparing *Ebf1*^WT^ and *Ebf1*^KO^ transcriptomes. Dots in blue represent genes with an FDR <0.1. The y axis represents shrunken log_2_ fold change (FC), capped at |log_2_ FC| 2, and the x axis represents log_10_ basemean. **(C and D)** Comparison of chromatin accessibility changes to gene expression changes in MPP3 (C) and MPP4 (D) cells upon *Ebf1* deletion. The y axis represents shrunken log_2_ fold changes of gene expression and the x axis represents log_2_ fold changes of peak read counts in *Ebf1*^WT^ versus *Ebf1*^KO^ MPP4 cells. Left: Dots represent DA peaks in MPP3 (C) and MPP4 (D) cells. Right: Dots represent DE genes and ATAC peaks that overlap their TSS (TSS peaks). **(E)** CFU assay performed with 150 sorted MPP3 and MPP4 cells plated in triplicate in methocult. Total number of colonies formed after 10–12 d of plating. Biological replicates *Ebf1*^WT^ and *Ebf1*^KO^ *n* = 6. Statistical significance was determined by unpaired *t* test. **(F)** Heatmap showing gene expression of DE genes in MPP3 and MPP4 cells, between *Ebf1*^WT^ and *Ebf1*^KO^ conditions. Genes are organised into MPP3-specific, common, and MPP4-specific DE genes. Genes with an annotated C/EBPα ChIP peak (original data from Hasemann et al. [2014]; Pundhir et al. [2018]) are labeled in blue. Heatmap scale represents *z*-scores calculated separately for MPP3 and MPP4 cells. **(G)** qPCR analysis of *Cebpa* expression in *Ebf1*^WT^ and *Ebf1*^KO^ MPP3 cells transfected with a control or a *Cebpa* siRNA pool. *Cebpa* mRNA expression relative to *Actb* was normalized to the control transfected samples. Data are represented as mean ± SEM. *Ebf1*^WT^ and *Ebf1*^KO^ *n* = 4. **(E and G)** Data are from >2 independent experiments.

**Figure 4.**
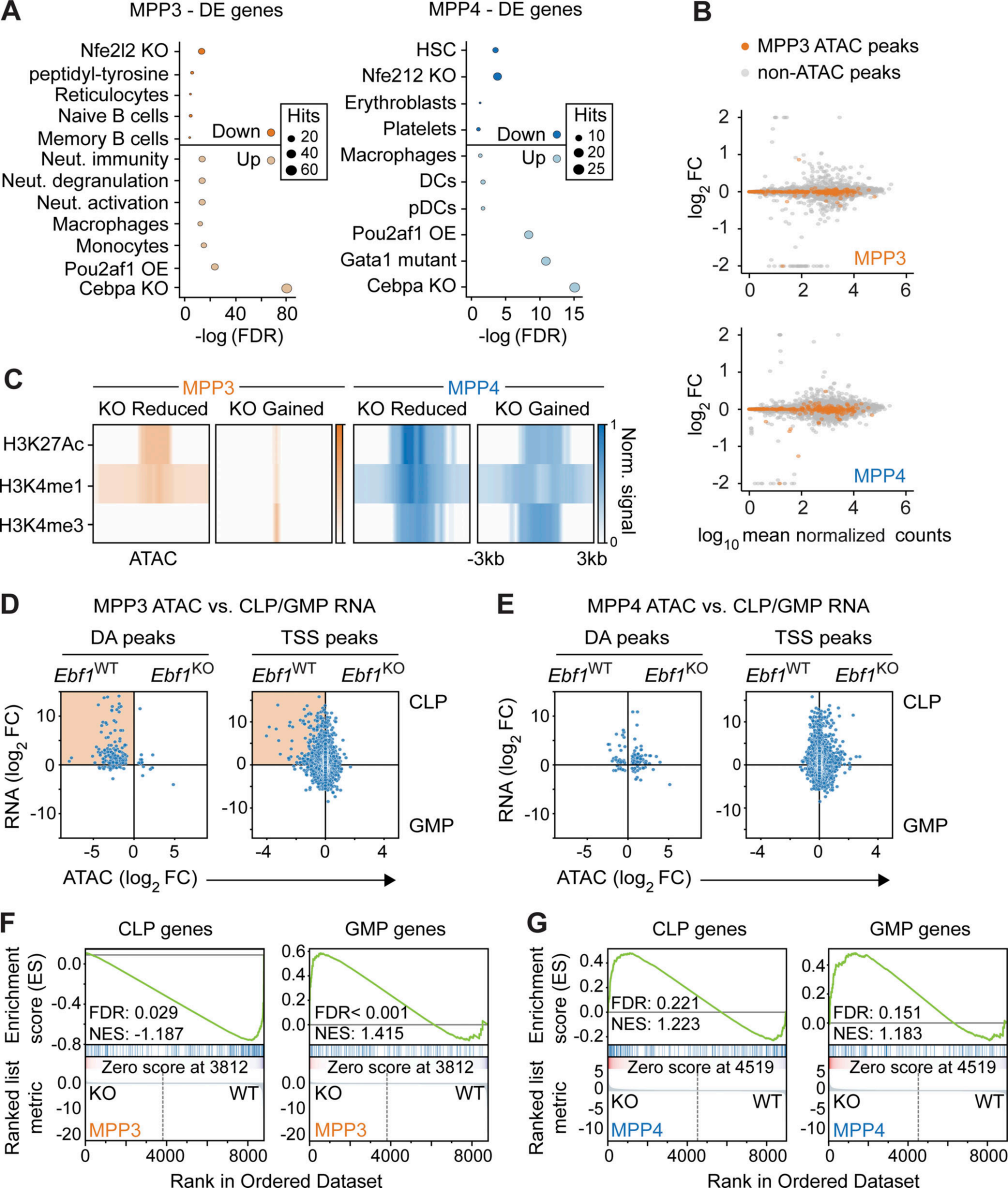
**EBF1-dependent chromatin sites in MPP3 cells are associated with expression in CLPs. (A)** Selected terms are represented from enrichment analysis of DE genes (FDR < 0.1) between *Ebf1*^WT^ and *Ebf1*^KO^ MPP3 (left) and MPP4 (right) cells. Enrichr datasets (GO_Biological_Process_2021, PanglaoDB_Augmented_2021, Gene_Perturbations_from_GEO_down) were used for enrichment analysis. Biological replicates *n* = 4. **(B)** Expression analysis of genes annotated to KO Reduced MPP3 peaks identified in ATAC analysis, in *Ebf1*^WT^ and *Ebf1*^KO^ MPP3 (upper panel), and MPP4 (lower panel) cells. The x axis represents log_10_ mean normalized counts of all genes. The y axis represents shrunken |log_2_ fold change| values of all genes in *Ebf1*^WT^ versus *Ebf1*^KO^ MPP3 (upper panel), and MPP4 (lower panel) cells, and is capped at 2. Genes in orange indicate the genes annotated to EBF1-dependent accessibility sites in MPP3 cells. **(C)** Heatmap displaying average ChIP signal for chromatin marks at DA peaks. Original data from Lara-Astiaso et al. (2014). **(D and E)** Comparison of chromatin accessibility changes in MPP3 cells (D) and MPP4 (E) cells to gene expression changes in GMP versus CLP cells. The y axis represents shrunken log_2_ fold changes of gene expression in GMP versus CLP cells. The x axis represents log_2_ fold changes of peak read counts in *Ebf1*^KO^ versus *Ebf1*^WT^ MPP3 (D) and *Ebf1*^KO^ versus *Ebf1*^WT^ MPP4 (E) cells. Highlighted box shows positive correlation of KO Reduced MPP3 peaks and CLP gene expression. **(D and E)** Left: Dots represent DA peaks in MPP3 and MPP4 cells. Right: Dots represent DE genes and ATAC peaks that overlap their TSS (TSS peaks). **(F and G)** GSEA results of all MPP3 (F) and MPP4 (G) ATAC peaks predicted to be enhancers (CRUP probability >0.8), ranked by log_2_ fold change, against a CLP-specific gene set (left) and a GMP-specific gene set (right). Positive enrichment scores reflect enrichment of the gene set in *Ebf1*^KO^ cells, negative enrichment scores reflect enrichment of the gene set in *Ebf1*^WT^ cells. CLP and GMP RNA-seq data analyzed in D–G was retrieved from GEO dataset GSE162662.

We turned to bulk RNA-seq of MPP3 and MPP4 progenitors to understand the molecular consequences of *Ebf1* deletion in HSPCs (Table S3). Principal component analysis (PCA) of the transcriptomes reveals moderate *Ebf1*-related changes (aligned with PC2: 8% of variance) compared to the differences between MPP3 and MPP4 populations (PC1: 80% of variance; Fig. S3 A). In *Ebf1*^KO^ MPP3 cells, we found 113 downregulated and 102 upregulated genes relative to *Ebf1*^WT^ MPP3 cells, and in *Ebf1*^KO^ MPP4 cells, we identified 152 downregulated and 153 upregulated genes as compared to *Ebf1*^WT^ cells (false discovery rate [FDR] <0.1; Fig. S3 B). By an enrichment analysis against various signatures, we find that the *Ebf1*^KO^-downregulated genes in MPP3 cells are enriched for B cell–identity signatures, which is in line with the chromatin accessibility analysis, although the number of genes is limited (Fig. 4 A). Interrogation of genes showing reduced ATAC peaks in *Ebf1*^KO^ MPP3 cells with the transcriptome analysis indicated that roughly half of these genes are weakly expressed in MPP3 cells (Fig. 4 B). Moreover, we found that impaired chromatin accessibility is not associated with DE genes in *Ebf1*^KO^ versus *Ebf1*^WT^ MPP3 cells (Fig. 4 B). This poor correlation between altered chromatin accessibility and gene expression was applied to both TSS-associated and DA ATAC peaks (Fig. S3 C). These observations were comparable for MPP4 cells (Fig. 4 B and Fig. S3 D). Thus, the genes associated with EBF1-dependent peaks detected in MPP3 cells are weak and not DE in MPP3 or MPP4 cells.

Using datasets of chromatin modifications in HSPC populations (Lara-Astiaso et al., 2014), we found that ATAC peaks reduced or gained in *Ebf1*^KO^ MPP4 cells were marked by H3K27ac, H3K4me1, and H3K4me3 modifications, suggesting that the peaks coincide with promoter regions (Fig. 4 C). Interestingly, peaks reduced in *Ebf1*^KO^ MPP3 cells were marked by H3K27ac and H3K4me1 but not by H3K4me3 modifications, suggesting that these chromatin regions may coincide with active enhancers (Fig. 4 C). This observation raised the question of whether EBF1-dependent accessible sites in MPP3 cells are associated with genes that are expressed in further lineage-restricted differentiation intermediates. Therefore, we interrogated the ATAC peak signals in *Ebf1*^WT^ and *Ebf1*^KO^ MPP3 cells with changes in gene expression that occur between CLP and GMP progenitors. This analysis indicated a strong association of EBF1-dependent ATAC peaks in MPP3 cells (DA peaks) with a gain of gene expression in CLPs relative to GMPs (Fig. 4 D). A corresponding analysis of ATAC peaks at TSS in MPP3 cells revealed a less pronounced association with the CLP-specific gene expression, consistent with the preferential EBF1-mediated priming of enhancers in MPP3 cells (Fig. 4 D). An association between CLP gene expression and ATAC peak signals in *Ebf1*^WT^ and *Ebf1*^KO^ MPP4 cells was not observed (Fig. 4 E). To further assess the association of enhancers in MPP3 and MPP4 cells with downstream gene expression, we generated a myeloid and lymphoid signature using genes that are DE between GMP and CLP progenitors. Then, we selected ATAC peaks that are annotated as enhancers in MPP3 and MPP4 cells. With these means, gene set enrichment analysis (GSEA) analysis showed that enhancer ATAC peaks in *Ebf1*^WT^ MPP3 cells were specifically enriched for the CLP signature (Fig. 4 F), which was not observed for *Ebf1*^WT^ MPP4 cells (Fig. 4 G). Taken together, these results indicate that EBF1-dependent chromatin accessibility in MPP3 cells is associated with enhancers that confer gene expression in the lymphoid lineage-restricted CLP compartment.

### *Ebf1* deficiency enhances the C/EBPα-driven myeloid bias of MPP3 and MPP4 cells

**Figure 5.**
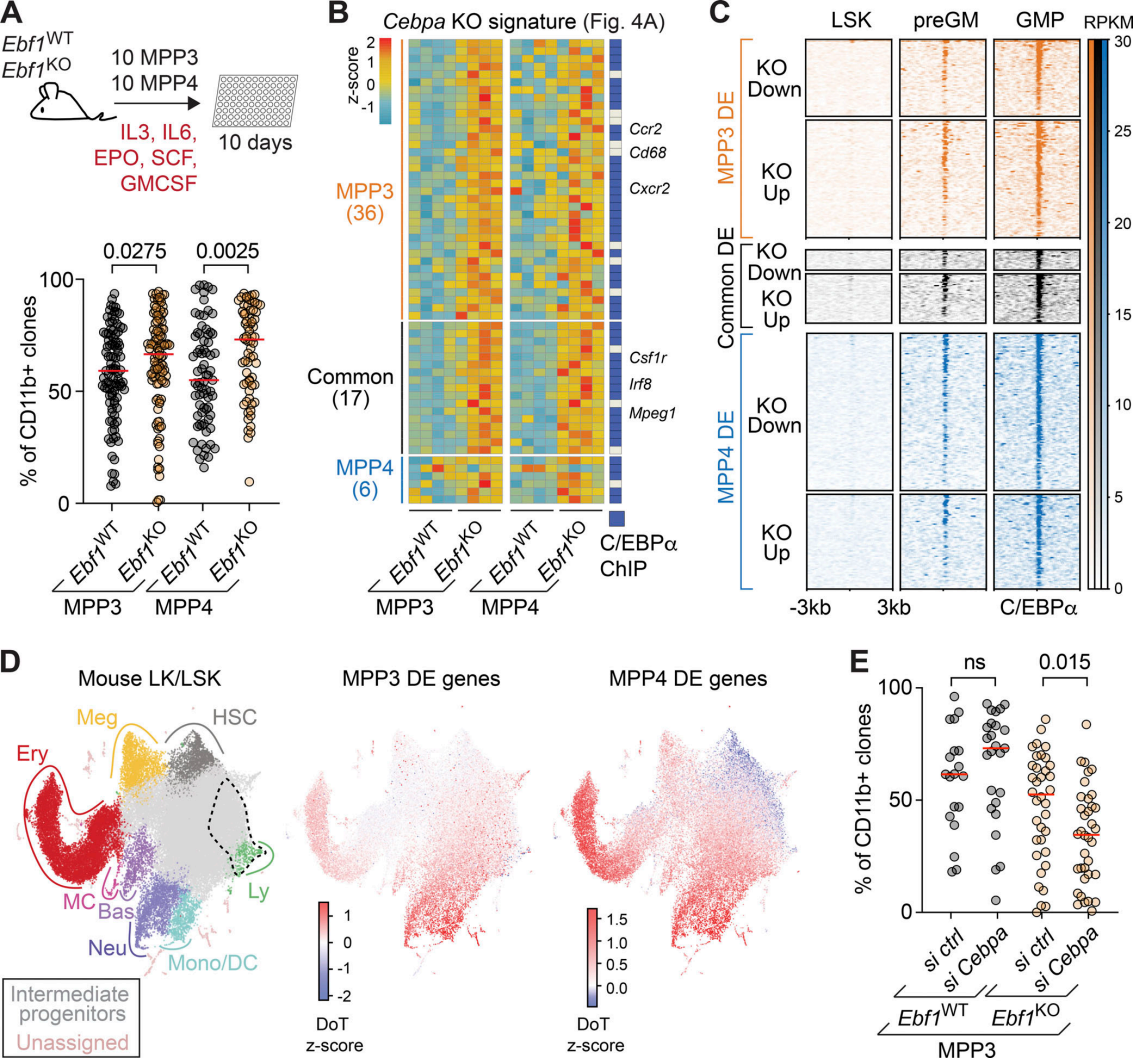
**Increased myeloid bias in *Ebf1***^**KO**^ **MPP3 and MPP4 cells is enhanced by C/EBPα. (A)** Workflow of myeloid differentiation assay with cytokines (IL3, IL6, EPO, GMCSF, SCF) of MPP3 and MPP4 (upper). Frequency of CD11b^+^ cells after 10 d of differentiation (lower). Red lines indicate the medians. Data obtained from four mice with following colony numbers: *Ebf1*^WT^ MPP3 *n* = 99, *Ebf1*^KO^ MPP3 *n* = 108, *Ebf1*^WT^ MPP4 *n* = 73, *Ebf1*^KO^ MPP4 *n* = 69. **(B)** Heatmap showing gene expression of DE genes associated with *Cebpa* KO signature identified through Enrichr enrichment analysis in Fig. 4 A. Genes are organized into MPP3-specific, common, and MPP4-specific DE genes. Genes with an annotated C/EBPα ChIP peak are labeled in blue. Heatmap scale represents *z*-scores calculated separately for MPP3 and MPP4 cells. **(C)** ChIP-seq analysis of C/EBPα occupancy in LSKs, preGM, and GMP. C/EBPα peaks are organized into MPP3-specific, common, and MPP4-specific DE genes. Regions ± 3 kb around C/EBPα summits are shown. Heatmap scale represents read coverage. Original data from Hasemann et al. (2014); Pundhir et al. (2018). **(D)** Left: Annotated UMAP projection of a scRNA-seq landscape: mouse LK + LSK populations (Dahlin et al., 2018) used as a reference for DoT score analysis; dashed area indicates the point of origin. DoT *z*-score values for DE genes between MPP3 *Ebf1*^WT^ versus *Ebf1*^KO^ (middle) and MPP4 *Ebf1*^WT^ versus *Ebf1*^KO^ (right). Red indicates a shift toward that cell fate and blue indicates a shift away from that cell fate. **(E)** Frequency of CD11b^+^ cells after 10 d of differentiation following transfection with a control or a *Cebpa* siRNA pool. Red lines indicate the medians. Data obtained from four mice with following colony numbers: *sictrl Ebf1*^WT^ MPP3 *n* = 19, *siCebpa Ebf1*^WT^ MPP3 *n* = 25, *sictrl Ebf1*^KO^ MPP3 *n* = 36, *siCebpa Ebf1*^KO^ MPP3 *n* = 36. **(A and E)** Statistical significance was determined by Mann-Whitney *U* test. Data are from >2 independent experiments. Meg, megakaryocyte; Ery, erythrocyte; MC, mast cell; Bas, basophil; Neu, neutrophil; Mono/DC, monocyte/dendritic cell; Ly, lymphocyte.

The impaired lymphoid chromatin landscape of *Ebf1*^KO^ MPP3 cells and the enrichment of myeloid molecular signatures (neutrophil activation) and macrophage cell-identity signatures in the enrichment analysis of the *Ebf1*^KO^ MPP3 and MPP4 transcriptomes raised the question of whether or not these cells have an enhanced myeloid bias. In addition, GSEA analysis found that enhancer ATAC peaks in *Ebf1*^KO^ MPP3 cells were specifically enriched for the GMP signature (Fig. 4 F), although this was not significant for enhancer ATAC peaks in *Ebf1*^KO^ MPP4 cells (Fig. 4 G). To assess whether MPP3 and MPP4 cells have an enhanced myeloid bias upon *Ebf1* deletion, we evaluated their myeloid differentiation potential in vitro by culturing them in myeloid differentiating conditions and by evaluating CD11b surface expression after 10 d (Fig. 5 A). In line with their RNA-seq profiles, *Ebf1*^KO^ MPP3 and MPP4 cells both showed higher frequencies of CD11b^+^ cells, indicating an increased myeloid potential of these EBF1-deficient progenitors (Fig. 5 A). This increase in myeloid potential was dependent on GM-CSF (data not shown). However, in a CFU assay, the total number of colonies generated by MPP3 and MPP4 cells showed no difference between *Ebf1*^WT^ and *Ebf1*^KO^ cells (Fig. S3 E).

The similar increase in the myeloid bias of *Ebf1*^KO^ MPP3 and MPP4 cells contrasted the MPP3-specific EBF1 dependence of B-lymphoid priming. To explore the common enhanced myeloid bias of MPP3 and MPP4 cells, we found an overlap of 66 DE genes (Fig. S3 F), and we noted that the most significantly enriched term associated with the upregulated genes was a signature previously connected with the downregulation of genes upon *Cebpa* deletion (Fig. 4 A). Notably, many of the genes that were inversely deregulated in *Ebf1*^KO^ progenitors and *Cebpa*-deficient cells, including *Irf8* and *Csf1r*, are linked to myeloid differentiation (Fig. 5 B). We also found that more genes belonging to the *Cebpa* signature were significantly upregulated in *Ebf1*^KO^ MPP3 than in *Ebf1*^KO^ MPP4 cells, although a similar trend was observed in *Ebf1*^KO^ MPP4 cells. (Fig. 5 B). To assess whether the upregulated genes in *Ebf1*^KO^ cells are C/EBPα-bound targets, we overlapped these genes with public C/EBPα ChIP-seq datasets of HSPC progenitors (LSKs) and myeloid progenitors (pre-GM and GMP; Hasemann et al., 2014; Pundhir et al., 2018). This analysis showed that almost all of the genes associated with the *Cebpa* signature in *Ebf1*^KO^ MPP3 and MPP4 cells are bound by C/EBPα (Fig. 5 B). We also overlapped the *Ebf1*^KO^/*Ebf1*^WT^ DE genes of MPP3 and MPP4 cells with the C/EBPα occupancy in LSK, pre-GMs, and GMPs and found that the DE genes were mainly associated with C/EBPα occupancy in GMPs. Moreover, C/EBPα occupancy was mainly associated with upregulated genes in *Ebf1*^KO^ MPP3 cells and associated with downregulated genes in *Ebf1*^KO^ MPP4 cells (Fig. 5 C and Fig. S3 F), suggesting the enhanced myeloid bias directed by C/EBPα is stronger in *Ebf1*^KO ^MPP3 cells.

C/EBPα is essential for monocyte–neutrophil specification, and *Cebpa* KO mice display a block in pre-GM progenitors (Zhang et al., 1997; Paul et al., 2015; Pundhir et al., 2018). Analogous to the function of EBF1 in antagonizing alternative lineages in early B cells, C/EBPα represses non-myeloid lineages, whereby its expression increases from pre-GM to GMP progenitors and reaches the highest levels in neutrophils (Paul et al., 2015). To estimate the direction of perturbation upon *Ebf1* deletion, we calculated a Direction of Transition (DoT) score, anchored at the lymphoid portion of the landscape marked by the dotted line, for *Ebf1*^KO^/*Ebf1*^WT^ DE genes of MPP3 and MPP4 cells (Fig. 5 D; Kucinski et al., 2020). Analogous to term-enrichment analysis of DE genes, the DoT score analysis uses a single-cell RNA-seq (scRNA-seq) landscape from a publicly available LK/LSK dataset (Dahlin et al., 2018) as a reference to visualize similarities between cell states. Positive values in red indicate a transition of *Ebf1*^KO^ conditions toward those cell fates, and negative values in blue indicate a transition of *Ebf1*^KO^ conditions away from those cell fates. In line with the enrichment analysis, the DoT score analysis showed that *Ebf1*^KO^ MPP3 and MPP4 cells shift toward a neutrophil, monocyte/dendritic progenitor cell transcriptional state (Fig. 5 D). These data suggest that *Ebf1*-deficient MPP3 and MPP4 cells upregulate genes that are targets of C/EBPα in GMPs, which may account for the increased myeloid potential of *Ebf1*^KO^ progenitors. To functionally examine whether C/EBPα is responsible for the increased myeloid bias observed in *Ebf1*^KO^ MPP3 cells, we performed knockdown of *Cebpa* in *Ebf1*^WT^ and *Ebf1*^KO^ MPP3 cells, followed by in vitro myeloid differentiation assay. Upon *Cebpa* knockdown in *Ebf1*^KO^ MPP3 cells, we observed a decrease in the frequency of CD11b^+^ cells relative to control knockdown conditions (Fig. 5 E and Fig. S3 G). No significant effect was observed in *Ebf1*^WT^ MPP3 cells (Fig. 5 E). These data suggest that the increased myeloid bias observed in *Ebf1*^KO^ MPP3 cells may, at least in part, be driven by C/EBPα.

### Impaired B-lymphoid priming and expansion of myeloid-biased progenitors

**Figure 6.**
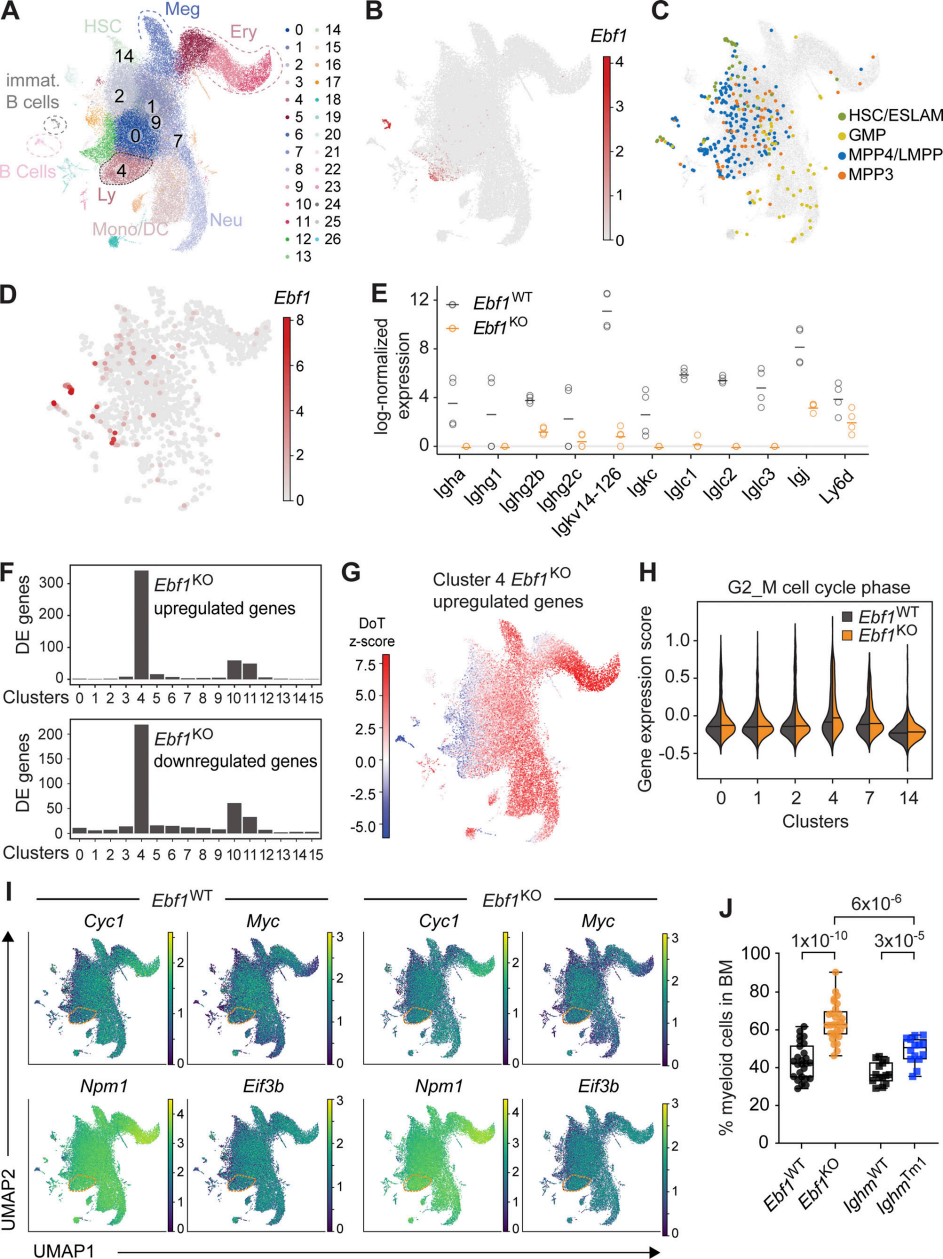
***Ebf1* controls lymphoid/myeloid balance in HSPCs, and its loss causes increased myeloid cells in the BM. (A and C)** Annotated UMAP projection of scRNA-seq landscape derived from *Ebf1*^WT^ and *Ebf1*^KO^ LK and LSK populations. Biological replicates *n* = 4. **(B and D)** Log-normalized *Ebf1* expression for *Ebf1*^WT^ cells shown in A and C, respectively. **(C)** Cells from Nestorowa data (Nestorowa et al., 2016; colored) were embedded into the *Ebf1*^WT^/*Ebf1*^KO^ landscape (gray) with annotated immunophenotypic populations. **(E)** Log-normalized expression of Ig and lymphoid-associated DE genes in *Ebf1*^WT^ and *Ebf1*^KO^ cells of selected clusters. Each dot represents the mean expression per mouse (four mice in total). **(F)** Number of DE genes between *Ebf1*^WT^ and *Ebf1*^KO^ cells per cluster. **(G)** DoT scores calculated using DE genes in cluster 4 between *Ebf1*^WT^ and *Ebf1*^KO^ cells in the context of the LK/LSK landscape. Mean expression in cluster 4 was used as the point of origin. Red indicates a shift toward that cell fate and blue indicates a shift away from that cell fate. **(H)** Violin plots showing the gene expression score of G2/M cell cycle–associated genes in *Ebf1*^WT^ and *Ebf1*^KO^ cells in selected clusters. **(I)** UMAP visualization of the expression of *Myc* and selected *Myc* target genes in *Ebf1*^WT^ and *Ebf1*^KO^ cells. **(J)** Boxplots showing the frequency of myeloid cells (CD11b^+^) in the BM. *Ebf1*^WT^ *n* = 18, *Ebf1*^KO^ *n* = 16, *Ighm*^WT^ and *Ighm*^Tm1^ *n* = 11. Statistical significance was determined by Mann–Whitney *U* test. Data are from >2 independent experiments. LK defined as Lin-cKit^+^ cells. ESLAM, CD45^+^EPCR^+^CD48^−^CD150^+^ HSCs; Meg, megakaryocyte; Ery, erythrocyte; Neu, neutrophil; Mono/DC, monocyte/dendritic cell; Ly, lymphocyte.

**Figure S4.**
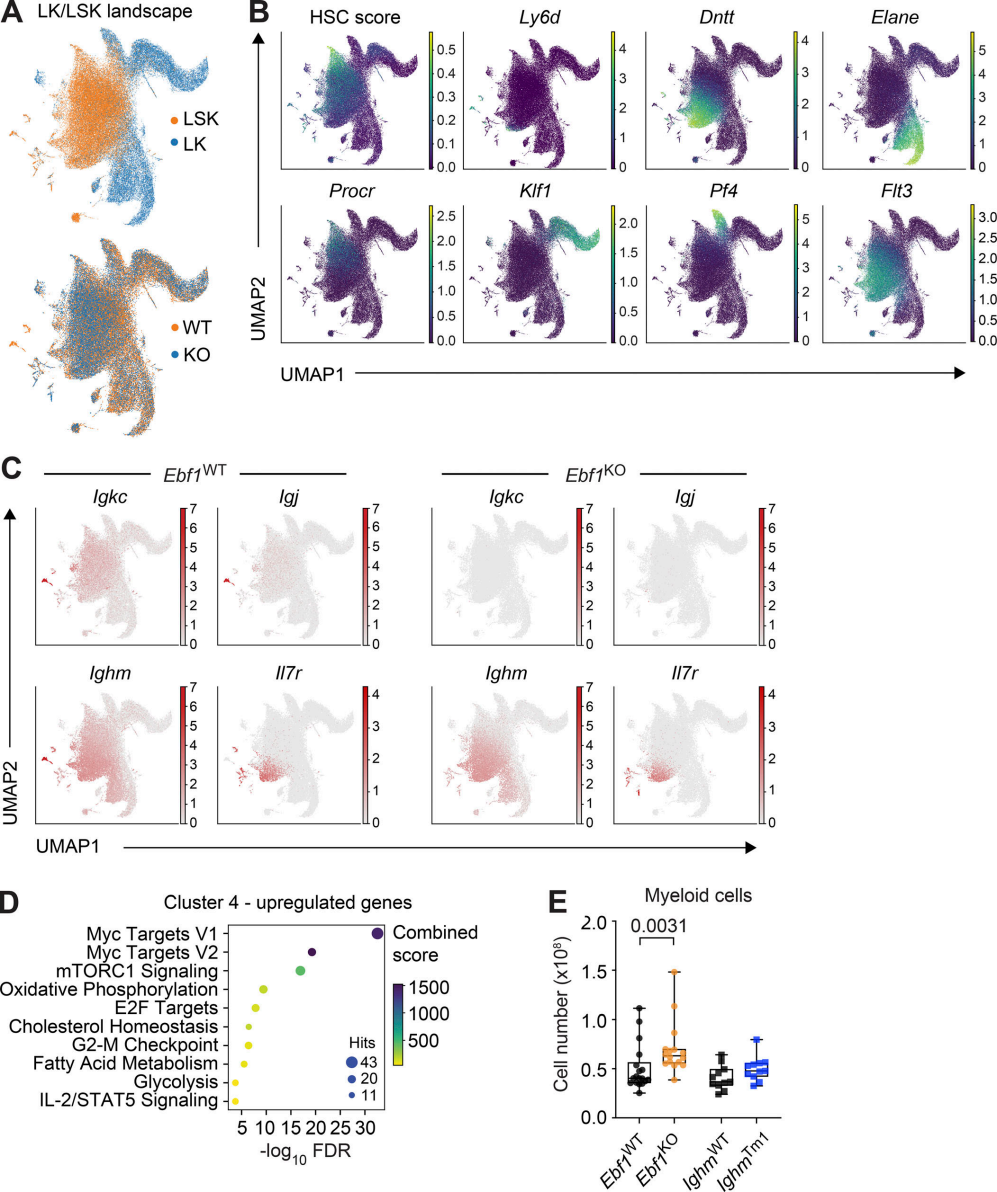
**Characterization of the scRNA-seq landscape in *Ebf1*^WT^ and *Ebf1*^KO^ LSK and LK cells.** Related to Fig. 6. **(A)** scRNA-seq landscape derived from *Ebf1*^WT^ and *Ebf1*^KO^ LK and LSK populations, annotated for LSK and LK cells (top) and *Ebf1*^WT^ and *Ebf1*^KO^ cells (bottom). **(B)** UMAP visualisation of the HSC score and the expression of lineage marker genes used for the identification of cell clusters (*Procr*, *Dntt*, *Elane*, *Klf1*, *Pf4*, *Flt3, Ly6d*). **(C)** UMAP visualisation of the expression of *Il7r* and Ig genes in *Ebf1*^WT^ and *Ebf1*^KO^ cells. **(D)** GO enrichment analysis of the upregulated genes in cluster 4, between *Ebf1*^WT^ and *Ebf1*^KO^ cells. **(E)** Boxplots showing the absolute number of myeloid cells (CD11b^+^) in the BM. *Ebf1*^WT^ *n* = 18, *Ebf1*^KO^ *n* = 16, *Ighm*^WT^ and *Ighm*^Tm1^ *n* = 11. Statistical significance was determined by Mann-Whitney *U* test. Data are from >2 independent experiments.

To obtain deeper insights into the HSPC cell states affected by the *Ebf1* deletion, we performed scRNA-seq of the LK and LSK compartments, comprising myeloid progenitors and HSPCs. We analyzed 59,505 high-quality cells in four replicates per genotype. We integrated all data and generated a common landscape with clustering and reduced dimensionality representation: Uniform Manifold Approximation and Projection (UMAP; analogously to Dahlin et al., 2018; Kucinski et al., 2020). The landscape, annotated with lineage markers and an HSC score (Hamey and Göttgens, 2019), showed gradual progression from the HSCs to committed myeloid progenitors (Fig. 6 A and Fig. S4, A and B). The *Ebf1*^WT^ and *Ebf1*^KO^ cells did not show significant changes in abundance across the major landscape populations (Fig. S4 A). *Ebf1* was predominantly expressed in cluster 4, corresponding to lymphoid progenitors within the LSK compartment (Fig. 6 B). By making use of an index-sorted scRNA-seq HSPC dataset (Nestorowa et al., 2016), we embedded the MPP3 cells and MPP4/lympho-myeloid primed progenitor (LMPP) cells into our landscape (Fig. 6 C). MPP4/LMPP cells were found to be enriched on the left side of the landscape territory, which shows elevated expression levels of lymphoid genes, including *Dntt* and *Flt3* (Fig. 6 C and Fig. S4 B). MPP3 cells were uniformly distributed, with a few cells belonging to the lymphoid cluster 4 (Fig. 6, A and C). The Nestorowa et al. (2016) dataset had been generated with the Smart-Seq2 technology, therefore, we took advantage of its higher detection rate to observe *Ebf1* expression at a higher resolution. Supporting the notion of a continuum of progenitors and the existence of *Ebf1*-expressing MPP3 and MPP4 cells, we observed *Ebf1* expression in the lymphoid-progenitor cluster 4, but also low-level *Ebf1* expression scattered in clusters upstream of lineage-restriction, such as clusters 2 and 1 (Fig. 6, A and D).

Next, we examined whether *Ebf1* deletion has consequences on gene expression in clusters representing progenitors of lower lineage restriction, in which a putative lymphoid/myeloid choice would occur. In particular, we focused on clusters 0, 1, 2, and 9, in which we observed lower levels of *Ebf1* expression than in cluster 4. Joint differential expression analysis identified a limited number of 17 genes, which surprisingly included 11 genes encoding Igκ light chain, Ig J chain, and other B-lymphoid markers (Fig. 6 E and Fig. S4 C). All of these genes were found to be downregulated to undetectable or barely detectable levels following *Ebf1* deletion (Fig. 6 E). The reduction of Ig gene expression upon *Ebf1* deletion was detected before the activation of the main lymphoid program, annotated by markers like *Dntt*, *IL7r*, and *Ly6d* (Fig. S4, B and C), and was also detected in bulk RNA-seq analysis of MPP3 and MPP4 cells (Table S3).

Finally, we investigated changes in gene expression for each cluster between *Ebf1*^WT^ and *Ebf1*^KO^ cells. Appropriately, we detected the majority of DE genes in cluster 4, in which *Ebf1* was expressed at the highest level (Fig. 6 F). We calculated the DoT score for the DE genes of cluster 4, anchoring the origin point in the same cluster. We observed strong positive score values in the myeloid and erythroid parts of the landscape, indicating a shift toward these states and away from the lymphoid state (Fig. 6 G). Moreover, the analysis of enriched GO terms among genes upregulated in cluster 4 in *Ebf1*^KO^ cells showed a strong enrichment for molecular signatures related to proliferation, such as *Myc*, increased metabolic pathways, E2F targets, and G2-M checkpoint (Fig. S4 D). Accordingly, we detected increased expression of G2-M cell-cycle phase–associated genes, specifically in *Ebf1*^KO^ cells of cluster 4 (Fig. 6 H and Table S4). We observed increased expression of *Myc* and *Myc* target genes, such as *Cyc1* and *Eif3b*, in cells of the myeloid and erythroid parts of the landscape, but not in the HSC cluster (Fig. 6 I). We also observed an increase in *Myc* and *Myc* target gene expression in *Ebf1*^KO^ but not *Ebf1*^WT^ cells of cluster 4, whereas a similar expression pattern was observed in the other clusters (Fig. 6 I). The proliferative advantage of myeloid-biased *Ebf1*^KO^ cells was mirrored by the significant increase in the frequencies and absolute numbers of myeloid cells in the BM of *Ebf1*^KO^ mice relative to *Ebf1*^WT^ mice (Fig. 6 J and Fig. S4 E). Although the frequencies of myeloid cells were also increased in the BM of *Ighm*^Tm1^ mice relative to *Ighm*^WT^ mice, the increase was more pronounced in *Ebf1*^KO^ mice relative to *Ebf1*^WT^ mice (Fig. 6 J).

### EBF1 binds the *Cebpa* +37 kb hematopoietic regulatory region

**Figure 7.**
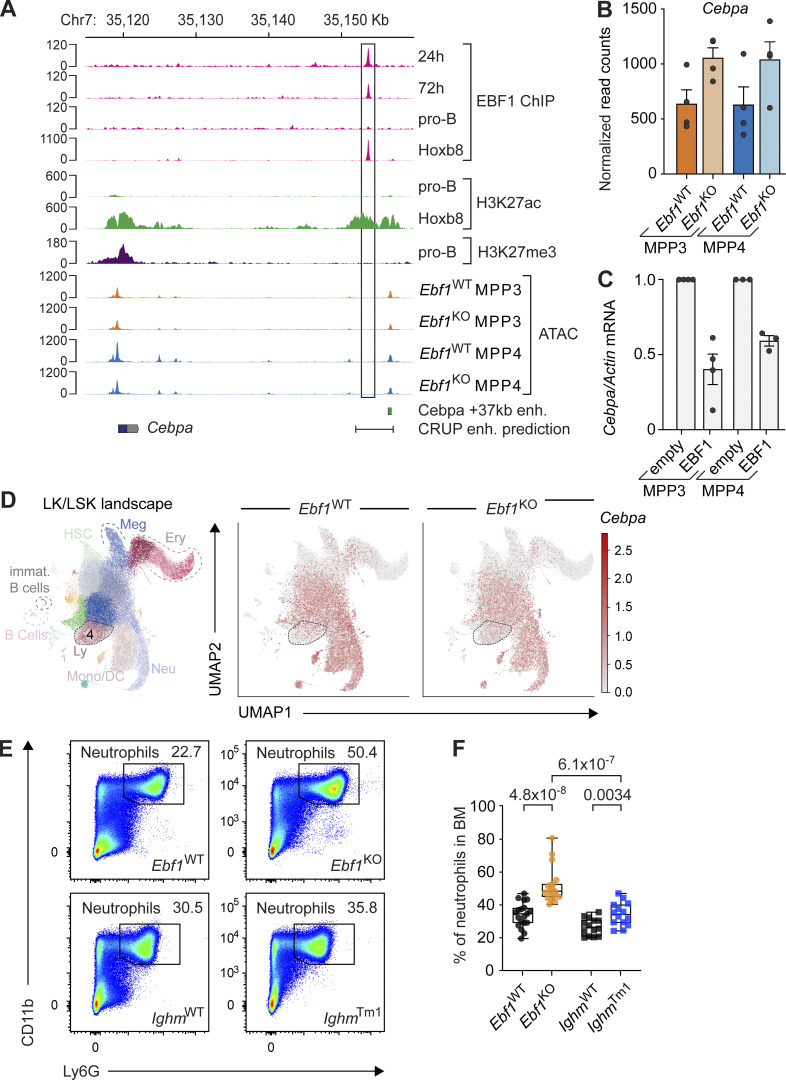
**EBF1 limits C/EBPα driven myeloid bias in MPP3 and MPP4 cells. (A)** *Cebpa* gene body and regulatory region is displayed, showing EBF1 occupancy 24 and 72 h after EBF1 induction, in pro-B cells and Hoxb8-FL cells; H3K27ac marks in pro-B cells and Hoxb8-FL cells; H3K27me3 marks in pro-B cells (original data from Li et al. [2018]; Kucinski et al. [2020]). Chromatin accessibility in *Ebf1*^WT^ MPP3, *Ebf1*^KO^ MPP3, *Ebf1*^WT^ MPP4, and *Ebf1*^KO^ MPP4 populations. The +37 kb *Cebpa* enhancer is annotated, as well as the enhancer region predicted by CRUP. The scale of the y axis represents RPKM in ChIP-seq and ATAC-seq tracks. **(B)** Normalized read counts of *Cebpa* in MPP3 and MPP4 populations, in *Ebf1*^WT^ and *Ebf1*^KO^ conditions. **(C)** qRT-PCR analysis of *Cebpa* expression in *Ebf1*^WT^ MPP3 and MPP4 cells transduced with an empty or an EBF1 vector. *Cebpa* mRNA expression relative to *Actb* was normalized to the empty vector transduced samples. Data are represented as mean ± SEM. *Ebf1*^WT^ and *Ebf1*^KO^ *n* = 3–4. **(D)** Left: Annotated UMAP projection of scRNA-seq landscape derived from *Ebf1*^WT^ and *Ebf1*^KO^ LK and LSK populations. Right: UMAP visualization of *Cebpa* expression in *Ebf1*^WT^ and *Ebf1*^KO^ cells. **(E)** Representative pseudocolor plots showing the frequency of Ly6G+ neutrophils in *Ebf1*^WT^, *Ebf1*^KO^, *Ighm*^WT^, and *Ighm*^Tm1^ mice. **(F)** Boxplots showing the percentage of neutrophils in the bone marrow. *Ebf1*^WT^ and *Ebf1*^KO^ *n* = 19, *Ighm*^WT^ and *Ighm*^Tm1^ *n* = 14. Statistical significance was determined by Mann-Whitney *U* test. **(C and F)** Data are from >2 independent experiments.

**Figure S5:**
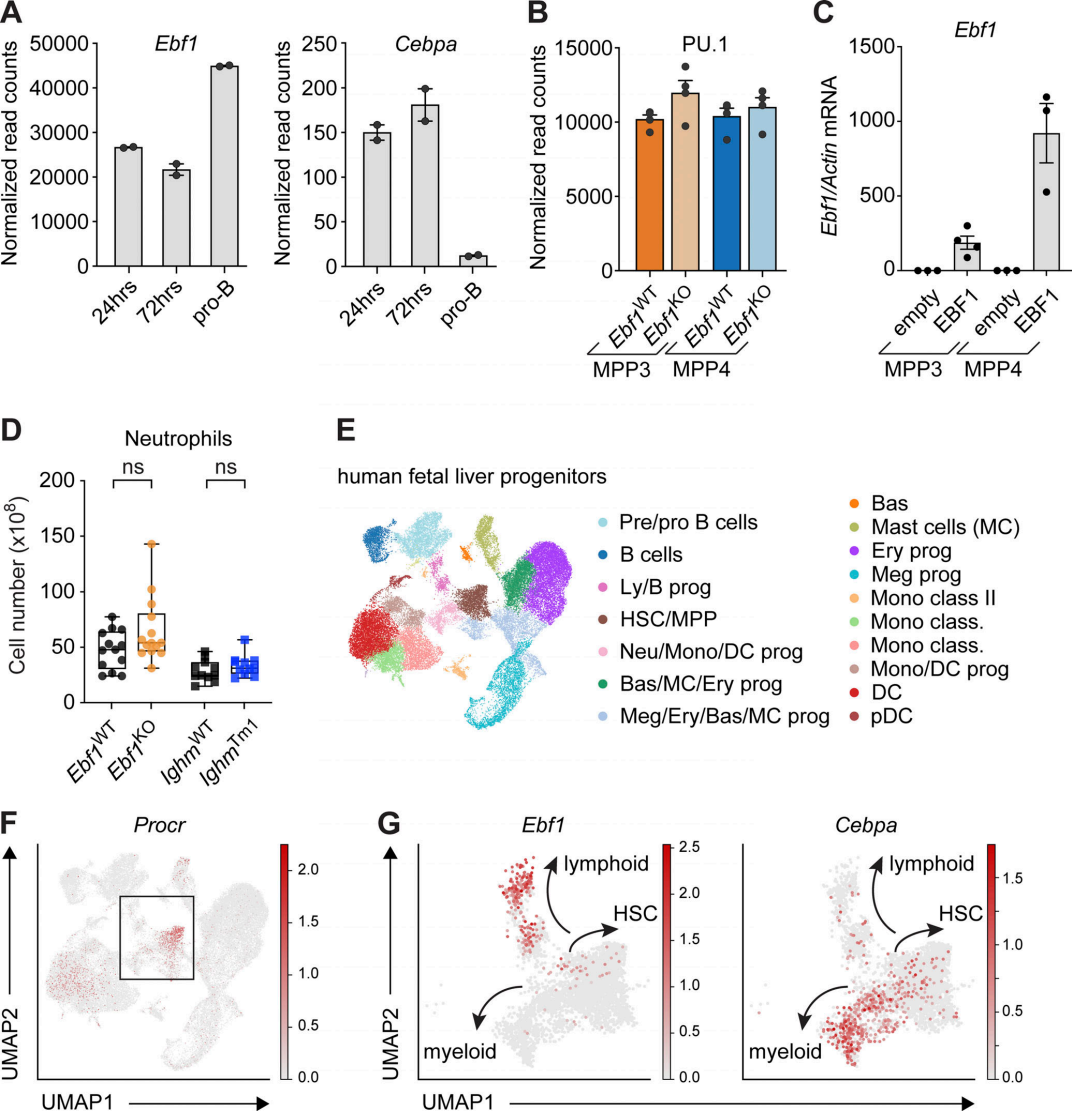
**Extended analysis of the *Ebf1*-*Cebpa* regulatory relationship.** Related to Fig. 7. **(A)** Normalized read counts of *Ebf1* (left) and *Cebpa* (right) in *Ebf1* ^−/−^ pre-pro-B cells 24 and 72 h after EBF1 induction, and pro-B cells (original data from Li et al. [2018]). **(B)** Normalized read counts of *Spi1* (PU.1) in MPP3 and MPP4 cells in *Ebf1*^WT^ and *Ebf1*^KO^ populations. **(C)** qRT-PCR analysis of *Ebf1* expression in *Ebf1*^WT^ MPP3 and MPP4 cells transduced with an empty or an EBF1 vector. *Ebf1* mRNA expression relative to *Actb* was normalized to the empty vector transduced samples. Data are represented as mean ± SEM. *Ebf1*^WT^ and *Ebf1*^KO^ *n* = 3–4. **(D)** Boxplots showing the absolute number of neutrophils (Ly6G^+^) in the BM. *Ebf1*^WT^ *n* = 13, *Ebf1*^KO^ *n* = 14, *Ighm*^WT^ and *Ighm*^Tm1^ *n* = 11. Statistical significance was determined by Mann-Whitney *U* test. **(C and D)** Data are from >2 independent experiments. **(E)** Annotated UMAP projection of scRNA-seq landscape derived from human fetal liver progenitors. **(F)** UMAP visualization of the expression of *Procr* in human fetal liver progenitors. Box indicates selected clusters (HSC/MPP, Ly/B progenitors, and Neu/Mono/DC progenitors) shown in G. **(G)** UMAP visualization of the expression of *Ebf1* and *Cebpa* in selected clusters of human fetal liver progenitors. **(E–G)** Original data from Popescu et al. (2019). Meg, megakaryocyte; Ery, erythrocyte; Bas, basophil; Neu, neutrophil; Mono, monocyte; DC, dendritic cell; Ly, lymphocyte; prog, progenitors; pDC, plasmacytoid DC.

The +37 kb *Cebpa* enhancer is responsible for hematopoietic expression of *Cebpa* (Cooper et al., 2015; Guo et al., 2016). Interestingly, C/EBPα has been implicated in inducing *Ebf1* expression (Guo et al., 2018; Barberi et al., 2020). In addition, EBF1 has been shown to bind within a H3K27ac-marked predicted regulatory region of the *Cebpa* gene in Hoxb8-FL cells (Kucinski et al., 2020). To further investigate the potential regulation of *Cebpa* by EBF1, we analyzed previous EBF1 ChIP-seq datasets for EBF1 binding at the *Cebpa* regulatory region in pro-B cells and *Ebf1*^−/−^ cKit^+^ progenitors, in which a doxycycline-responsive ectopic *Ebf1* gene was induced for 24 or 72 h (Li et al., 2018). In the progenitors with induced EBF1 expression, we detected EBF1 binding within the *Cebpa* regulatory region, adjacent to the +37 kb *Cebpa* enhancer, whereas neither EBF1 binding nor H3K27ac marks were found in pro-B cells (Fig. 7 A). Furthermore, *Cebpa* expression was decreased in pro-B cells relative to progenitors in which EBF1 expression was induced for 24 or 72 h (Fig. S5 A). Although the +37 kb *Cebpa* enhancer showed similar accessibility in *Ebf1*^WT^ and *Ebf1*^KO^ MPP3 and MPP4 cells, the EBF1-bound site was found to reside in inaccessible chromatin, consistent with other EBF1-repressed targets that are transiently bound before silencing (Fig. 7 A; Li et al., 2018). We observed no accumulation of H3K27me3 at the *Cebpa* regulatory region in pro-B cells, suggesting that the regulatory region does not become epigenetically silenced (Fig. 7 A). Moreover, the abundance of *Cebpa* transcripts, but not that of *Spi1*/PU.1 transcripts, increased in *Ebf1*^KO^ MPP3 and MPP4 cells relative to *Ebf1*^WT^ cells (Fig. 7 B and Fig. S5 B). Finally, overexpression of EBF1 in *Ebf1*^WT^ MPP3 and MPP4 cells resulted in decreased *Cebpa* expression compared to empty vector (Fig. 7 C and Fig. S5 C). Together, these data raise the possibility that EBF1 binding at the *Cebpa* regulatory region results in the downregulation of *Cebpa* expression.

Accordingly, we observed an increase in *Cebpa*-expressing *Ebf1*^KO^ cells in cluster 4 (Fig. 7 D). Corresponding to the C/EBPα-driven myeloid differentiation observed in *Ebf1*^KO^ MPP3 cells, we observed an increase in the frequencies and absolute numbers of neutrophils in the BM of *Ebf1*^KO^ mice relative to *Ebf1*^WT^ mice (Fig. 7, E and F; and Fig. S5 D). Although an increase in the frequencies of neutrophils was also observed in the BM of *Ighm*^Tm1^ mice relative to *Ighm*^WT^ mice, the frequencies of neutrophils in *Ebf1*^KO^ mice were significantly higher than in *Ighm*^Tm1^ mice (Fig. 7, E and F). This suggests an additive effect of B cell depletion and *Ebf1* deletion on neutrophil differentiation in *Ebf1*^KO^ mice.

To address whether a similar *Ebf1*–*Cebpa* axis could exist in human hematopoietic progenitors, we analyzed a public data set of human fetal liver progenitors (Popescu et al., 2019). Consistently, the HSC/MPP cluster marked by *Procr* expression also contains mRNA for *Ebf1* and *Cebpa* (Fig. S5, E–G). Altogether, our scRNA-seq and chromatin profiling analysis suggest that *Ebf1* takes part in B-lymphoid enhancer priming and in an attenuation of the myeloid fate potential driven by C/EBPα in MPP3 cells.

## Discussion

The functions of lineage-determining transcription factors, including EBF1, have been well studied at the onset of lineage specification. However, their potential roles in generating lineage bias of stem and progenitor cells are still largely obscure. Here, we show that the B cell determinant EBF1 is expressed at a low level in MPPs, MPP3 and MPP4, that have a myeloid and lymphoid bias, respectively. We find that the deletion of *Ebf1* in all hematopoietic cells results in an increased myeloid bias of both MPP3 and MPP4 populations, which may be attributed to a loss of EBF1 binding at the *Cebpa* enhancer region and the increased expression of the myeloid transcription factor gene *Cebpa*. In addition, we find that EBF1 is required for the de novo accessibility and priming of B-lymphoid enhancers specifically in MPP3 cells. This result is in line with extensive literature on the importance of EBF1 in establishing the B-lymphoid transcriptional program in pre-pro-B cells (Medina et al., 2004; Pongubala et al., 2008; Hagman et al., 2011; Welinder et al., 2011; Boller et al., 2018). However, it also introduces the additional dimension that EBF1 is responsible for the priming of the B-lymphoid fate in MPPs. Finally, we observe a diminished functionality of HSCs in the BM *Ebf1*-deficient mice, which may be an indirect consequence of the augmented myeloid potential of MPPs and/or the enhanced myelopoiesis.

Our single-cell analysis of LK/LSK cells from *Ebf1*^WT^ and *Ebf1*^KO^ cells revealed relatively strong *Ebf1* expression in lymphoid progenitors (cluster 4) and weak *Ebf1* expression scattered among early progenitors of the LSK clusters, in which lymphoid/myeloid lineage bias may occur. Moreover, scRNA-seq data suggested that EBF1 expression increases in the MPP continuum of MPP3 and MPP4 cells. We show through multiple lines of evidence that as EBF1 expression increases, EBF1 plays a role in limiting myeloid differentiation. While ATAC analysis of *Ebf1*^KO^ MPP3 cells points to a loss of B lymphoid priming, we also observed an enhanced myeloid signature of chromatin accessibility that is associated with a GMP pattern of gene expression. This augmented myeloid chromatin landscape is paralleled by the *Cebpa*-dependent enhanced capacity of *Ebf1*^KO^ MPP3 and MPP4 cells to generate CD11b^+^ myeloid cells in vitro. Moreover, our scRNA-seq data indicate that the *Ebf1* deficiency in progenitors of lymphoid cluster 4 results in an increase of *Cebpa* expression, a myeloid fate enrichment, and an increase in proliferation, consistent with the accumulation of mature CD11b^+^ myeloid cells and neutrophils in *Ebf1*^KO^ mice. However, we did not observe in increase in the total number of colonies in a CFU assay with MPP3 and MPP4 cells from *Ebf1*^WT^ and *Ebf1*^KO^ mice. The total number of colonies does not take into account the size or type of the colonies generated and therefore, it is possible that other parameters would reflect a myeloid bias in *Ebf1*^KO^ MPP3 and MPP4 cells. A role of *Ebf1* in cell proliferation has been previously shown in pro-B cells (Györy et al., 2012), and *Ebf1* has been implicated in participating with *Myc* and *Max* in distinct transcription modules to regulate DNA replication and cell cycle (Kucinski et al., 2020). Moreover, a hematopoietic enhancer cluster in the *Myc* locus is bound and regulated by EBF1 (Bahr et al., 2018; Ramamoorthy et al., 2020; Somasundaram et al., 2021).

Insight into the molecular basis for the enhanced myeloid basis was provided by the bulk RNA-seq analysis of MPP3 and MPP4 cells, which showed an increased expression of a large set of genes that have been previously identified as C/EBPα target genes in GMPs (Ye et al., 2015). C/EBPα is a lineage-determining transcription factor critical for the neutrophilic/monocytic cell fate (Zhang et al., 1997; Paul et al., 2015; Pundhir et al., 2018). Notably, the DoT-score analysis of the bulk RNA-seq data, evaluating similarities with cell states among the scRNA-seq landscape of WT LSK cells, indicated that *Ebf1*^KO^ MPP3 and MPP4 cells gain neutrophil–monocytic fate potential. Moreover, the knockdown of *Cebpa* in *Ebf1*^KO^ MPP3 cells suggests a decreased myeloid differentiation potential relative to the non-targeting control knockdown in *Ebf1*^KO^ MPP3 cells. However, we did not observe an increase in myeloid differentiation of control knockdown *Ebf1*^KO^ MPP3 cells relative to control knockdown *Ebf1*^WT^ MPP3 cells. Thus, further approaches will be needed to address the *Cebpa*–*Ebf1* relationship in MPP3 cells.

The increased expression of C/EBPα target genes, together with the binding of EBF1 to a *Cebpa* enhancer region upon transient EBF1 induction in *Ebf1*^*−/−*^ progenitors and in Hoxb8-FL cells, suggests that EBF1 may antagonize *Cebpa* expression in hematopoietic progenitors (this study; Kucinski et al., 2020; Li et al., 2018). In particular, transient EBF1 occupancy in *Ebf1*^*−/−*^ progenitors has been associated with alternative lineage genes that are silenced in committed pro-B cells (Li et al., 2018). In agreement with a transient binding of EBF1 in HSPCs, *Ebf1* has been predicted as a silencer of the +37 kb *Cebpa* enhancer (Bertolino et al., 2016; Repele et al., 2019). A repressive function of EBF1 in this context is also reflected by the decreased expression of *Cebpa* and the lack of H3K27ac marks along the *Cebpa* regulatory region in WT pro-B cells. In addition, the overexpression of EBF1 in MPP3 and MPP4 cells results in a reduction of *Cebpa* expression. In contrast to our findings, *Cebpa* expression was not increased upon *Ebf1* knock-down in Hoxb8-FL cells, which could be due to a partial depletion of EBF1 and/or due to differences in the chromatin accessibility at the EBF1 binding site (Kucinski et al., 2020). The EBF1 binding site in the *Cebpa* regulatory region is accessible in Hoxb8-FL cells, but not in *Ebf1*^−/−^ cKit^+^ progenitors and MPP3 and MPP4 progenitors (this study; Kucinski et al., 2020; Li et al., 2018). Of note, while C/EBPα is not expressed in B cells, it is expressed in lymphoid progenitors and is required to initiate *Ebf1* expression in CLPs (Guo et al., 2018; Barberi et al., 2020), indicating these transcription factors function in a transcriptional regulatory network. In this network, we hypothesize that EBF1 restrains the expression of C/EBPα to regulate the balance between B lymphoid and myeloid potential in MPPs. According to this scheme, changes in the concentrations of either of these counteracting transcription factors would shift the balance of myeloid versus lymphoid lineage bias. In support of an opposing function of EBF1 and C/EBPα, ectopic expression of C/EBPα in committed B cells leads to their rapid transdifferentiation to macrophages, which includes the downregulation of EBF1 and PAX5 (Xie et al., 2004; Laiosa et al., 2006).

Changes in the function of transcription factors by altered gene dosage have been extensively shown for the myeloid determinant PU.1 which regulates B-lymphoid versus myeloid cell fates in a graded manner (Laslo et al., 2006). High concentrations of PU.1 favor the macrophage developmental program, whereas low concentrations of PU.1 are important for B-lymphoid development (DeKoter and Singh, 2000; Pang et al., 2018). In this context, low levels of PU.1 are achieved by repression of PU.1 by Gfi1 (Laslo et al., 2006). EBF1 functions also in a dose-dependent manner as *Ebf1*^+/−^ heterozygosity results in a diminished B-lymphoid output (Lukin et al., 2011; Åhsberg et al., 2013). The low-level expression of *Ebf1* in MPPs may involve its regulation by Ikaros and PU.1, whereas the high-level expression of *Ebf1*, observed in CLPs, may be governed by additional IL7Rα signaling and regulation by E2A and FOXO1 (Seet et al., 2004; Dias et al., 2005; Kikuchi et al., 2005; Lin et al., 2010; Mansson et al., 2012). Resembling the dosage-dependent function of EBF1 on B cells, low-level expression of EBF1 in MPPs versus high-level expression in CLPs and pro-B cells may account for the disparate function of EBF1 in multipotent and B lineage–restricted hematopoietic progenitors.

The enrichment of the myeloid GMP signature was associated specifically with *Ebf1*-deficient MPP3 enhancers and not with *Ebf1*-deficient MPP4 enhancers. Moreover, the set of EBF1-dependent sites of chromatin accessibility at B-lymphoid genes was found to be specific to MPP3 cells. Since EBF1 is expressed at similarly low levels in both cell populations, the question arises as to why the EBF1 dependence of B-lymphoid enhancer priming is detected only in MPP3 progenitors. The differential chromatin accessibility sites of *Ebf1*^WT^ and *Ebf1*^KO^ MPP4 cells displayed strong PU.1 binding and were independent of EBF1 re-expression, suggesting that other pioneer factors, such as PU.1, could maintain accessibility at these chromatin sites. In addition, MPP3 and MPP4 (LMPP) cells also contain similar levels of *Cebpa* and *Spi1* (PU.1*)* transcripts; however, they differ in the abundance of *Ikaros* transcripts. Ikaros is an important regulator of all lymphoid lineages, and Ikaros deficiency results in impaired lymphoid cell differentiation at the LMPP stage (Winandy et al., 1995; Yoshida et al., 2006). Therefore, it is possible that Ikaros acts independently of EBF1 in regulating the lymphoid potential in MPP4/LMPP cells. However, ectopic expression of EBF1 can rescue the impaired B cell differentiation of Ikaros-deficient mice (Reynaud et al., 2008), suggesting overlapping regulatory functions of both transcription factors.

Finally, we find that the hematopoietic deletion of *Ebf1* leads to impaired HSC quiescence and activation of HSCs, along with a diminished HSC repopulation capacity. While we observed an increase in myeloid-biased MPP3 cells and CD11b^+^ cells in the BM of *Ebf1*^KO^ mice, this increase was not observed in recipients of *Ebf1*^KO^ HSCs. This is likely due to the dominant effect of an impaired self-renewal capacity compared to a shift in lineage bias of *Ebf1*^KO^ HSPCs under homeostatic conditions. Although the effects of the *Ebf1* deletion on HSC function are compelling and robust, they are likely secondary to the EBF1-driven changes in MPP3 and MPP4 cells because *Ebf1* expression was not detected in HSCs. Recently, acute immune stimulation by LPS has been shown to induce persistent changes in the myeloid potential of exposed HSCs (de Laval et al., 2020). Moreover, the effects on HSC functionality are not due to the absence of B cells as no obvious changes were observed in *Ighm*^Tm1^ mutant mice, in which a signaling-incompetent Igµ heavy chain results in an early block of B cell differentiation (Kitamura et al., 1991). Therefore, we favor the view that the enhanced myeloid bias and/or expansion of the myeloid compartment in the BM leads to the activation of HSCs. Consistent with this view, inflammatory signaling has been found to result in activation of HSCs (Mirantes et al., 2014; McCabe and MacNamara, 2016; Mitroulis et al., 2018; Hormaechea-Agulla et al., 2020; Bousounis et al., 2021). However, the addition of *Ebf1*^WT^ and *Ebf1*^KO^ BM fluid to WT HSCs or co-culture with myeloid cells (data not shown) did not influence HSC proliferation, suggesting that the effects of EBF1 deficiency on HSC homeostasis may be independent of changes in soluble cytokines.

In conclusion, our study emphasizes the importance of EBF1 in regulating myeloid/lymphoid fate bias in MPPs by constraining C/EBPα-driven myelopoiesis and priming the B-lymphoid fate. This expression pattern is reflected in human hematopoietic progenitors. Upon aging, EBF1 expression decreases (Lescale et al., 2010; Lescale et al., 2015; Riley, 2013) and hematopoiesis is shifted toward an enhanced myeloid output (Snoeck, 2013; Yamamoto et al., 2013; Lee et al., 2019; Dorshkind et al., 2020; Mejia-Ramirez and Florian, 2020). This altered EBF1 expression may be involved in aging-associated changes of lymphoid and myeloid trajectories.

## Materials and methods

### Mice

*Ebf1-flox* mice were generated as detailed in Györy et al. (2012). *Tie2-Cre* (Kisanuki et al., 2001) and Ighm^Tm1^, also known as *muMt*^*−/−*^ mice (Kitamura et al., 1991), were purchased from The Jackson Laboratory; stock no. 008863 and 002288, respectively. Only male heterozygous *Tie2*^*Cre*^*Ebf1*^*wt/fl*^ mice were used for breeding, as litters with female *Tie2-Cre* drivers demonstrated germline *Ebf1* deletion. *muMt*^*+/+*^ littermates were used as controls for the Ighm strain and flox/flox or +/Cre littermates were used as controls for the *Tie2*^*Cre*^*Ebf1*^*flox*^ strain. C57BL/6J (CD45.1, CD45.2, or CD45.1.2) mice were bred in-house. 8–14-wk-old animals of both sexes were used for experiments. All mice were maintained, bred, and analyzed on the C57BL/6J background in the animal facility of the Max Planck Institute of Immunobiology and Epigenetics under specific pathogen–free conditions. Animals were housed on a 14-h/10-h light-dark cycle and provided with standard rodent chow and water ad libitum. All animal procedures were performed in compliance and approved by responsible Animal Welfare Committees (Regierungspräsidium Freiburg, Nr. 35-9185.81/G-18/104, Nr. 35-9185.81/G-17/65).

### Cell suspensions and flow cytometry (cell cycle staining)

Single-cell suspensions were prepared from BM (femora, tibiae, and ilia bones) by crushing them in cold PBS-3%FCS using a pestle and mortar followed by erythrocyte lysis using RBC lysis buffer (BioLegend). For cell sorting, BM cells were depleted using a magnet, enriching for the lineage-negative fraction. Briefly, BM cells were incubated for 15 min with a biotin antibody cocktail (2 ml/mouse). Labeled cells were then incubated for 15 min with 50  µl of magnetic MojoSort Streptavidin Nanobeads (BioLegend). In the case of HSPC collection for Western blot, cells were prepared in cold PBS-1%BSA, and labeled cells were incubated with 400 µl/mouse of magnetic washed anti rat IgG-coated Dynabeads provided in the Dynabeads Untouched Mouse CD4 Cells Kit (Invitrogen). Lineage-negative cells were subsequently incubated for 30 min with the appropriate antibody cocktail to purify HSPCs. Following cell surface staining, intracellular staining of Ki67 and Hoechst was performed by fixing and permeabilizing with the eBioscience Foxp3/Transcription Factor Staining Buffer Set (eBioscience). For the homing assay and BM fluid culture assay, cells were labeled with 5 µM CTY (Invitrogen) for 20 min at room temperature. Surface antibodies were diluted in PBS-3%FCS, intracellular antibodies were diluted in permeabilization buffer (eBioscience). Between each step, cells were washed twice with PBS-3%FCS or permeabilization buffer and centrifuged at 400 *g* at 4°C. Flow cytometry analysis was performed on an LSRFortessa instrument and cell sorting on FACSAria III, FACSAria Fusion, or FACSymphony instruments (BD Biosciences). Data were analyzed with FlowJo Software V.10 (TreeStar). Antibodies were purchased from BD Biosciences, BioLegend, or Invitrogen; a list of antibodies used and population definitions can be found in Table S1, gating strategy for HSPCs in Fig. S1.

### BM AdT

Primary HSC AdT experiments were performed by injecting 150 donor HSCs (CD45.2) mixed with 500,000 supportive total BM cells (CD45.1) into the tail vein of recipients (CD45.1.2). Secondary HSC AdT experiments were performed by injecting 3 × 10^6^ total BM cells from primary recipients at 16 wk after AdT into the tail vein of secondary recipients (CD45.1.2).

Reverse AdT experiments were performed by injecting 5 × 10^6^ total BM cells from WT (CD45.1) donors into irradiated *Ebf1*^WT^ and *Ebf1*^KO^ (CD45.2) recipient mice.

Recipient mice (8–10-wk-old) were lethally irradiated with 9.5 Gy using a γ irradiator with a ^137^Cs source (Biobeam GM 8000) and injected within 24 h of irradiation. One donor was injected into two to three recipient mice, and for reverse AdT experiments, a single WT (CD45.1) donor was used per experiment. Donor hematopoietic reconstitution was monitored every 4 wk in peripheral blood by flow cytometry and sacrificed at 16–24 wk after AdT.

Homing assays were performed by injecting 50,000 sorted LSKs from *Ebf1*^WT^ and *Ebf1*^KO^ (CD45.2) mice, labeled with CTY (5 µM; Invitrogen) into recipients (CD45.1). Recipients were sacrificed 14–16 h after injection. Spleen, peripheral blood, and BM were analyzed for CTY donor contribution.

### 5-FU treatment

Mice were injected intraperitoneally with 5-FU (Sigma-Aldrich) at 150 mg/kg every 10 d over the course of 100 days. 5-FU is dissolved in DMSO, in 10% of the final volume, then immediately vortexed and shaken at 850 rpm at 40°C. After the 5-FU is dissolved, it is filtered through 0.2 μm and resuspended to the appropriate final volume of sterile PBS. Control mice were injected with 100 μl PBS per 10 g of mouse.

### Western blots

Cells were sorted directly into cold PBS-1%BSA and the cell pellet was frozen in liquid nitrogen before further processing. For whole cell extracts, cells were incubated for 15 min on ice in 150 mM NaCl, 1% NP-40, 50 mM Tris-HCl, pH 7.4, 1 mM EDTA, 0.25% deoxycholic acid, supplemented with complete protease inhibitor cocktail (Roche). After lysis, samples were sonicated in a water bath with 12 strokes and 50% output and incubated for another 15 min on ice. Cell debris was then removed by centrifugation at 16,000 *g* for 10 min at 4°C. Samples were denatured at 95°C for 5 min in NuPAGE LDS Sample Buffer (Invitrogen) before SDS-PAGE using NuPAGE 4–12% Tris-Glycine gels (Invitrogen) and then transferred to 0.45 μM polyvinylidene difluoride membranes (Bio-Rad). Membranes were blocked for 1 h at room temperature with 5% milk in PBS with 0.1% Tween (PBST). The following antibodies were used: anti-H3 (Abcam) and anti-EBF1 (in-house), which were raised against the first 14 amino acids of EBF1, as detailed in Roessler et al. (2007). Primary antibody incubation was performed overnight at 4°C, and secondary incubation with HRP conjugated anti-rabbit antibody (Invitrogen) was performed the next day at room temperature. Membranes were washed with PBST thrice between each antibody incubation. Antibodies were diluted in PBST-5% milk.

### Bulk CFU assays

150 LT-HSCs, HSCs, MPP3, or MPP4 cells were cultured in Methylcellulose Complete Media (R&D systems) in triplicate. CFU colonies were counted after 10–12 d in culture. Cultures were maintained at 37°C at 5% CO_2_.

### BM fluid cultures

300 HSCs labeled with CTY (5 μM; Invitrogen) were sorted into 96-well U-bottom plates. HSCs were cultured for 48 h in Stempro-34 SFM (Gibco) supplemented with cytokines stem cell factor (SCF; 50 ng/ml; Peprotech) and Thrombopoietin (TPO; 10 ng/ml; Peprotech) and the indicated amount of BM fluid. BM fluid was harvested by crushing clean bones in 400 μl cold PBS and cells were removed by centrifugation at 400 *g* for 5 min. Supernatant was collected with Microtainer SST tubes (BD Biosciences) at 12,000 g for 1 min and then stored at −80°C. HSC division was analyzed on day 2 and day 5; see Table S1 for antibody panel.

### Myeloid differentiation assay and siRNA knockdown of *Cebpa*

10 MPP3 and MPP4 cells were sorted into 96-well U-bottom plates. Cells were cultured for 10 d in Stempro-34 SFM (Gibco) supplemented with 10% StemPro Nutrient Supplement and cytokines SCF (50 ng/ml; Peprotech), IL-3 (5 ng/ml; Peprotech), Erythropoietin (EPO; 2 U/ml; BioLegend), IL-6 (10 ng/ml; Peprotech), and GM-CSF (10 ng/ml; Peprotech). Media with cytokines were refreshed on day 6. Differentiation was assessed by flow cytometry on days 10–12 (see Table S1 for antibody panel). Cultures were maintained at 37°C at 5% CO_2_. Lineage-negative cells from *Ebf1*^WT^ and *Ebf1*^KO^ mice were transfected with DharmaFECT1 (Dharmacon), with 25 nM control non-targeting or *Cebpa* siRNA (D-001810-10-05, L-040561-00-0005; Dharmacon) together with 25 nM siGLO Green (Dharmacon). After 48–72 h, 10 MPP3 and MPP4 siGLO Green-positive cells were sorted into 96-well U-bottom plates as described above.

### Retroviral expression of EBF1

Retroviral transduction was performed as previously described (Treiber et al., 2010). Briefly, retrovirus was produced by transient transfection of Plat-E cells with retroviral constructs pMYs-Ebf1-IRES-EGFP or pMYs-IRES-EGFP. Lineage-negative cells from *Ebf1*^WT^ and *Ebf1*^KO^ mice were resuspended in retroviral supernatant at 1–2 × 10^6^ cells/ml and centrifuged at 2600 rpm for 2–3 h in 6-well plates (2 ml/well). After spin infection, 70% of the viral supernatant was removed and cells were cultured with Stempro-34 SFM (Gibco) supplemented with cytokines SCF (50 ng/ml; Peprotech), TPO (10 ng/ml; Peprotech), and Flt3L (10 ng/ml; Peprotech). After 40 h, GFP-positive MPP3 and MPP4 cells were sorted for B cell differentiation assay or ATAC-seq.

### B cell differentiation assay

OP9-feeder cells were grown in MEM Alpha (Sigma-Aldrich) supplemented with 20% FCS, 1% sodium pyruvate, and 1% penicillin/streptomycin, L-glutamine. Feeder cells were grown until 80–90% confluent and treated with 10 μg/ml mitomycin C (Sigma-Aldrich) for 2 h. Cells were washed four times with PBS, detached with trypsin–EDTA (Gibco) at 37°C for 5 min, and seeded at ∼3,500 cells per well of a 48-well plate, 48 h before the experiment. 50 MPP3 and MPP4 cells were FACS sorted into the OP9-seeded 48-well plates. Cells were cultured in Opti-MEM medium (Gibco) supplemented with 4% FCS, 1% penicillin/streptomycin, L-glutamine, 50 μM β-mercaptoethanol, SCF (10 ng/ml; Peprotech), Flt3L (10 ng/ml; Peprotech), and IL-7 (5 ng/ml; Peprotech). Media was refreshed on day 6. Differentiation was assessed by FACS on days 6, 9, and 12.

### ATAC-seq

#### Generation

ATAC-seq was performed as described in Corces et al. (2017) with some variations. Briefly, 5,000 FACS-sorted cells were sorted into 100 µl PBS-1%FCS and spun down at 500 *g* for 10 min at 4°C and the supernatant was removed. The cell pellet was resuspended in a lysis/transposition buffer (1 µl Tagmentase, 10 µl 2× TD buffer [Diagenode], 0.01% digitonin [Promega], and 0.3× PBS) and incubated for 30 min at 37°C at 700 rpm. Reactions were cleaned up with Zymo DNA Clean and Concentrator −5 columns (Zymo Research). The remainder of the ATAC-seq library preparation was performed as described in Buenrostro et al. (2013); Buenrostro et al. (2015) using Nextera DNA UD Indexes (IDT). Libraries were cleaned up using AMPureXP beads (Beckman Coulter), and double-sided size selection was performed by removing large fragments that precipitate with 0.5× volume and purifying fragments that precipitate with 1.8× volume ratio. Two biological replicates were used for ATAC-seq from LT-HSCs, MPP3, and MPP4 cells from *Ebf1*^WT^ and *Ebf1*^KO^ mice. Four biological replicates were used for ATAC-seq from MPP3 cells from *Ebf1*^KO^ mice and transduced with empty or EBF1 overexpression vector. ATAC-seq paired-end 100 bp reads were generated using the Illumina NovaSeq 6000 system. Samples were first sequenced at shallow depth (∼5 million reads) to assess the quality before sequencing at a depth of 50 million reads. Reads from both flowcells were merged at the fastq level.

#### Analysis

The Nextera transposase adapters were trimmed from the raw FASTQ using Cutadapt (version 2.10 [Martin, 2011]) using flags -A CTGTCTCTTATA -a CTGTCTCTTATA. Alignments against the mm10 reference genome (ensembl release 91) were created using the DNA-mapping module implemented in snakePipes (version 2.5.1 [Bhardwaj et al., 2019]) using the --dedup --mapq 5 flags. Data quality was assessed using FastQC (version 0.11.9 [Andrews, 2010], MultiQC (version 1.8 [Ewels et al., 2016]), and deepTools (version 3.3.2 [Ramírez et al., 2016]). Analysis downstream was performed using a custom pipeline (https://github.com/maxplanck-ie/ATACofthesnake) implemented in snakemake (version 6.0.0 [Koster and Rahmann, 2012]). Briefly, fragments mapping to read attracting regions (Amemiya et al., 2019) or exceeding 150 bp (to capture nucleosome-free regions) were filtered out using the alignmentSieve module in deepTools. Subsequently, peaks were called using MACS2 (version 2.2.7.1 [Zhang et al., 2008]) with the settings --nomodel, --shift -75, --extsize 150 -g 1.87e9 -q 0.01 and --keep-dup all, and a union of peaks per cell type (MPP3 and MPP4) was created using bedtools merge (version 2.30.0 [Quinlan and Hall, 2010]). Genes were annotated to the closest gene using Uropa (version 4.0.0 [Kondili et al., 2017]), allowing a 20-kb distance upstream and 10-kb distance downstream. Count matrices were created using the multiBamSummary module in deepTools, and differential accessible regions were called using the glmQLFit and glmQLFT functions (with design: ∼genotype) from the edgeR package (version 3.32.0 [Robinson et al., 2010]) implemented in R (version 4.0.3 [R Core Team, 2020]). Heatmaps were visualized using the plotHeatmap function from deepTools after normalizing the alignments with the reads per kilobase per million mapped reads (RPKM) method implemented in the bamCoverage function from deepTools. Motif enrichment for DA peaks was performed with the ame method (MEME-suite, version 5.0.2 [McLeay and Bailey, 2010]), using the non-redundant vertebrate motif database from JASPAR (Fornes et al., 2019). As control sequences, the reciprocal differential peak sets were used. For the alternative enrichment analysis, a shuffled DA peak set was used by using the --control --shuffle-- flag. Motif clustering was done using the ClusterMotifs module implemented in TOBIAS (version 0.12.11 [Bentsen et al., 2020]), using flags -t 0.4, --dist_method seqcor --clust_method complete. Co-motif enrichment was assessed by counting overlapping consensus motif occurrences in either the differential peak set or 1,000 background sets of equal size containing a random subsample of all peaks. Consensus motif occurrences were considered overlapping if they were no further than 50 bp apart. Aggregation plots were created with the TOBIAS workflow, using the ATACorrect and PlotAggregate functions with default parameters. GSEA was performed using the prerank module implemented in GSEApy (version 0.10.5 [Subramanian et al., 2005]). A ranked list was created by subsetting ATAC peaks overlapping with predicted enhancer regions (see below) and by omitting peaks without any annotated gene. A rank value was calculated per ATAC peak by multiplying the log_2_ fold change with the -log_10_ FDR. The final list was deduplicated by retaining the gene symbol associated with the highest log_2_ fold change. The CLP and GMP gene sets were created by extracting 300 significant genes with the lowest FDR value (FDR < 0.05 and log_2_ fold change >2 or −2). GSEA was performed using 1,000 permutations. GO was performed using clusterProfiler (version 4.0.5 [Wu et al., 2021]) implemented in R on genes annotated to DA peak sets (KO Reduced and KO Gained, for MPP3 and MPP4 cells) with FDR < 0.1. Read coverages for track visualization were obtained via deepTools bamCoverage using RPKM normalization (binsize 5bp). For peak calling and visualization, replicates were merged per condition at the bam level.

### Public data integration

Publicly available data were collected for EBF1 ChIP experiments in 24 h, 72 h, and pro-B cells samples (GSE107242 [Li et al., 2018]), and Hoxb8-FL cells (GSE146128 [Kucinski et al., 2020]). Publicly available data were collected for H3K27Ac ChIP experiments in pro-B cells (GSE107242 [Li et al., 2018]) and in Hoxb8-FL cells (GSE146128 [Kucinski et al., 2020]), and for H3K4Me1, H3K4Me3, and H3K27Ac ChIP experiments in HSPC populations (GSE59636 [Lara-Astiaso et al., 2014]). Publicly available data were collected for C/EBPα ChIP experiments in LSK, preGMs, and GMP cells (GSE89767, GSE43007 [Hasemann et al., 2014; Pundhir et al., 2018]). Alignments against the mm10 reference genome (ensembl release 91) were performed using the DNA-mapping mode implemented in snakePipes (version 2.5.1), using the flags --trim, --dedup, --mapq 3, and --fastqc. Subsequently, the ChIP-seq mode from snakePipes was invoked using default parameters (pro-B cell data and Hoxb8 cell data), with the flag --singleEnd (HSPC data) and the flag --q value 0.01 (C/EBPα data). Metagene plots were created by combining either the input-subtracted bigwig files (pro-B cell and Hoxb8 experiments) or the RPKM-normalized bigwig files (HSPC experiments) per replicate using wiggletools (version 1.2.2 [Zerbino et al., 2014]) with the median function, followed by the plotProfile function within deeptools. The resulting EBF1 ChIP and histone modification ChIP profiles were subsequently scaled between 0 and 1 per ChIP over all the regions. Enhancer prediction was performed on replicate-merged bam (from histone modification ChIPs) files using the Condition-specific Regulatory Units Prediction (CRUP) algorithm (version 1.0 [Ramisch et al., 2019]), implemented in R (version 3.6.3) using default parameters. Only regions with a probability >0.8 were included for downstream analysis. Read coverages for track visualization were obtained via deepTools bamCoverage using RPKM normalization (binsize 5bp) and subsequently plotted using pyGenomeTracks (3.3 [Ramírez et al., 2018]). C/EBPα ChIP was analyzed further by unifying peaks over replicates. Genes were annotated to the closest gene using Uropa, allowing a 20-kb distance upstream and 10-kb distance downstream. Heatmaps were visualized using the plotHeatmap function from deepTools after normalizing the alignments with the RPKM method implemented in the bamCoverage function from deepTools (binsize 5 bp).

### RNA extraction and RT-qPCR

Total RNA was prepared using the RNA Clean & Concentrator −5 RNA isolation kit (Zymo Research) according to the manufacturer’s instructions and treated with DNase (Qiagen). cDNA was synthesized with Oligo(dT)_18_ primers using the SuperScript II Reverse Transcriptase Kit (Invitrogen). RT-PCR was performed using the GoTaq Green master mix (Promega). The external primers used were Ebf1F: 5′-CCA​GCC​CGT​GGA​GAT​TGA​GAG-3′, Ebf1R: 5′-TCT​TTC​ACA​TGG​GAG​GGA​CAA​TCA​T-3′ and Ash2lF: 5′-CAC​CTT​TGG​AAT​AGA​CAC​GTC​G-3′, Ash2lR: 5′-TCC​GGC​AGT​GAC​TTG​GCT​GTC-3′, yielding products ∼1,000–1,500 bp. The first round of PCR was carried out as follows: initial denaturation at 95°C for 2 min, followed by 10 cycles of denaturation at 95°C for 30 s, annealing at 62°C for 20 s, and extension at 72°C for 1.5 min, followed by a final extension at 72°C for 5 min. The second round of amplification was carried out using 2 μl of the first PCR product as a template and using the primers Ebf1RI: 5′-GAT​GAG​GCG​CAC​ATA​GAA​ATC​CTG​T-3′ and Ash2lFI: 5′-CTG​GTC​CCA​GCC​CTT​AGG​TAA​CC-3′, which amplify a 150–200 bp product. The same conditions and amplification program were followed as for the first round, except the extension time was 20 s, with an increasing number of cycles from 25 to 40 cycles. PCR products were resolved on 2% agarose gels.

qRT-PCR was performed with Taqman Gene Expression Assays (FAM; Thermo Fisher Scientific) with Taqman Fast Advanced Master Mix (Thermo Fisher Scientific) using target probes against *Ebf1* (Mm01288947_g1) or *Cebpa* (Mm01265914_s1) normalized to *Act2b* (Mm00607939_s1 Actb). Reactions were performed in duplicate and run on a StepOne Real-Time PCR system (Applied Biosystems).

### Bulk RNA-seq

MPP3 and MPP4 cells were sorted into 50 μl of extraction buffer. Total RNA was prepared using the Arcturus PicoPure RNA isolation kit (Applied Biosystems) according to the manufacturer’s instructions and treated with DNase (Qiagen). Four biological replicates were used for RNA-seq from MPP3 and MPP4 cells from *Ebf1*^WT^ and *Ebf1*^KO^ mice. RNA-seq libraries were prepared by the deep-sequencing facility (Max Planck Institute-Freiburg) using NEBNext SingleCell/Low Input RNA library prep kit (NEB). Bulk RNA-seq paired-end 100-bp reads were generated using the Illumina NovaSeq 6000 system at a depth of 50 million reads.

#### Analysis

Raw FASTQ files were mapped against the mm10 reference genome using the mRNA-seq module implemented in snakePipes (version 2.5.1 [Bhardwaj et al., 2019]), using the --trim flag. Briefly, reads were aligned using STAR (version 2.7.3a [Dobin et al., 2013]), followed by expression count quantification with featureCounts (version 2.0.0 [Liao et al., 2014]). Data quality was assessed using Deeptools QC (version 3.3.2 [Ramírez et al., 2016]). Genes with an average expression higher than 100 counts in any condition were selected for further analysis. Downstream differential expression analysis was performed using DESeq2 (version 1.28.1 [Love et al., 2014]) implemented in R (version 4.0.0), and ashr was used for LogFoldChange shrinkage of results (version 2.2.47 [Stephens, 2017]). Genes were considered DE at an FDR < 0.1. MPP3 and MPP4 cells from *Ebf1*^WT^ and *Ebf1*^KO^ mice were analyzed together. Publicly available data collected for HSPC *Ebf1* expression analysis were analyzed together (Sommerkamp et al., 2020 and GSE162662). Publicly available data collected for *Ebf1* and *Cebpa* expression analysis from *Ebf1*^−/−^ cKit^+^ progenitors in which a doxycycline-responsive ectopic *Ebf1* gene was induced for 24 or 72 h, and pro-B cells were analyzed together (Li et al., 2018). Normalized counts were extracted from the appropriate DEseq2 objects. PCA visualization was performed based on variance stabilized read counts using the DESeq2 package. Enrichment analysis was performed using Enrichr (Chen et al., 2013; Kuleshov et al., 2016) against selected databases (GO_Biological_Process_2021, PanglaoDB_Augmented_2021, and Gene_Perturbations_from_GEO_down). MA plots of genes associated with EBF1-dependent peaks detected in MPP3 cells were performed with an unfiltered count matrix to include lowly expressed genes. All figures were generated using the ggplot2 package (version 3.3.0 [Wickham, 2016]). Heatmaps showing the Z-scores of genes were calculated separately for MPP3 and MPP4 conditions and were generated using the pheatmap package (version 1.0.12 [Kolde, 2019]).

### scRNA-seq

An equal number of FACS-sorted LSK and LK cells were combined from a female mouse and from a male mouse of each genotype to generate the scRNA-seq library. The Chromium Single Cell 3′ Library & Gel Bead Kit v3.1, Chromium Single Cell 3′ Chip G kit v2, and Single Index Kit T Set A (10x Genomics), were used according to the manufacturer’s instructions. Briefly, 10,000 cells of each population/genotype were combined per sex (total cell number ∼ 20,000 per reaction) and mixed with RT reaction mix before being added to a chromium microfluidics chip. The chip was then placed within the chromium controller for Gel Bead-in-Emulsion generation. Reverse transcription was immediately performed within the oil droplets to produce barcoded first-strand cDNA. Silane magnetic beads are used to purify the barcoded first-strand cDNA before amplification by PCR. Sequencing libraries were generated from cleaned, amplified cDNA using the Chromium Next GEM Single Cell 3′ Library kit (10x Genomics), including reagents for fragmentation, adaptor ligation, and sample index PCR. Between each of these steps, libraries were cleaned and size-selected using AMPureXP beads (Beckman-Coulter). 10-wk-old *Ebf1*^WT^ and *Ebf1*^KO^ mice were pooled by opposite sex of the same genotype, resulting in four biological replicates from two independent experiments. scRNA-seq paired-end 100 bp reads were generated using the Illumina NovaSeq 6000 system at a depth of ∼30,000 paired-reads per cell.

#### Analysis

scRNA-seq data was analyzed using the Scanpy package (Wolf et al., 2018). The unique molecular identifier counts matrix (cell × genes) was obtained using the cellranger package (version 3.1.0) with default settings. Cells with <1,200 detected genes were excluded from further analysis. Count data were log-normalized and each cell was analyzed for the expression of *Xist* and Y-chromosome genes. Cells with Xist expression but no Y-chromosome gene expression were assigned female and vice versa. The small minority of cells expressing both Xist and Y-chromosome genes were excluded from the analysis as suspected doublets, and cells lacking expression of Xist and Y-chromosome genes were excluded. Data for the remaining 59,505 cells were used to generate a common scRNA-seq landscape as follows. 7,000 top variable genes (excluding *Ebf1*, *Xist*, Y-chromosome genes, and cell-cycle associated genes [as in Dahlin et al., 2018]) were used to compute 50 PCs. Batch effects were removed using the Harmony method (Korsunsky et al., 2018). The corrected components were used to identify eight nearest neighbors, which served as a basis for clustering (leiden algorithm) and UMAP (Traag et al., 2019; McInnes et al., 2020; https://github.com/TomKellyGenetics/leiden). Clusters were manually annotated using commonly used marker genes (see Dahlin et al., 2018) and the integration of Nestorowa HSPC scRNA-Seq data (Nestorowa et al., 2016; see below). Cell cycle scores were assigned using the score_genes_cell_cycle function in the Scanpy framework and gene sets from Macosko et al. (2015). Differential expression was performed using the pseudobulk method approach (Lun and Marioni, 2017) treating each cluster as a cell state (Crowell et al., 2020). Pseudobulk profiles were compared between KO and WT cells using edgeR (Robinson et al., 2010) quasi-likelihood F-test test, including covariates for sex and experimental batch. DE genes were called using the thresholds: FDR of 0.1 and |log_2_(Fold Change)| of 0.2. To find genes DE in the wider population of undifferentiated progenitors, we performed analogous analysis for combined clusters 0, 1, 2, and 9. Enrichment analysis was performed on DE genes separately for up- and down-regulated subsets using the Enrichr framework and GSEApy interface (Mootha et al., 2003; Subramanian et al., 2005; Chen et al., 2013; Kuleshov et al., 2016). DoT score was calculated as previously described (Kucinski et al., 2020) using point of origin indicated in the respective figures.

The human fetal liver data were obtained from ArrayExpress (E-MTAB-7407) and processed as previously described (Kucinski et al., 2020). Briefly, cells expressing <1,000 genes and with 10% counts aligned to the mitochondrial genes were filtered out. Based on the original annotation provided by the authors, only cells belonging to blood and endothelial populations were considered. 7,984 highly variable genes were used to compute 50 PCs, and batches with <400 cells were excluded. The remaining cells were integrated with batch-balanced k-nearest neighbor method (Polański et al., 2019).

### Smart-Seq2 data integration

We integrated immunophenotypically defined populations from the Nestorowa Smart-Seq2 dataset (Nestorowa et al., 2016; projected data) into our 10x Genomics scRNA-seq data (reference) using the Cellproject (https://github.com/Iwo-K/cellproject) framework. Briefly, Smart-Seq2 data were log-normalized analogously to the 10x data and corrected to match the expression space of the reference data using the Seurat batch correction method (Stuart et al., 2019). Subsequently, the reference PCA rotation matrix was used to compute PCs for the projected data, and these were used to identify 15 nearest neighbors between Smart-Seq2 and 10x data. Finally, we used the nearest-neighbor regression to predict PC coordinates in the Harmony-corrected PCA space (basis for our integrated landscape) and fitted Smart-Seq2 data into the reference UMAP coordinates.

### Statistical analysis

Data are presented as boxplots or medians, as indicated in the figure legends. Details of statistical tests and the exact replicate numbers are reported in the figure legends and/or figures. Except for sequencing analysis, all statistical analyses were performed using Prism 8 software (GraphPad).

### Code availability

All code used for data analysis in this manuscript are available at https://github.com/AurelieLen/EBF1_MPPs. ATAC analysis available at https://github.com/maxplanck-ie/ATACofthesnake. DoT score available at https://github.com/Iwo-K/dotscore. Cellproject available at https://github.com/Iwo-K/cellproject.

### Online supplemental material

Fig. S1 shows data related to Fig. 1 and Fig. 2. Fig. S2 shows data related to Fig. 3. Fig. S3 shows data related to Fig. 4 and Fig. 5. Fig. S4 shows data related to Fig. 6. Fig. S5 shows data related to Fig. 7. Table S1 contains a list of antibodies, reagents, and flow cytometry staining panels used in the study. Table S2 contains a list of the DA peak sets identified from bulk ATAC-seq of MPP3 and MPP4 cells from *Ebf1*^WT^ and *Ebf1*^KO^ mice. Table S3 contains a list of differentially regulated genes and normalized read counts identified from bulk RNA-seq of MPP3 and MPP4 cells from *Ebf1*^WT^ and *Ebf1*^KO^ mice, as well as the DEseq2 results table of the comparison of MPP3 and MPP4 cells from *Ebf1*^WT^ mice. Table S4 contains a list of genes used for the gene sets G2-M and S- cell cycle phase, related to Fig. 6 H.

## Supplementary Material

Table S1 contains a list of antibodies, reagents, and flow cytometry staining panels used in the study.

Table S2 contains a list of the DA peak sets identified from bulk ATAC-seq of MPP3 and MPP4 cells from *Ebf1*^WT^ and *Ebf1*^KO^ mice.

Table S3 contains a list of differentially regulated genes and normalized read counts identified from bulk RNA-seq of MPP3 and MPP4 cells from *Ebf1*^WT^ and *Ebf1*^KO^ mice, as well as the DEseq2 results table of the comparison of MPP3 and MPP4 cells from *Ebf1*^WT^ mice.

Table S4 Contains a list of genes used for the gene sets G2-M and S- cell cycle phase, related to Fig. 6 H.

SourceData F1 contains original blots for Fig. 1.

SourceData FS2 contains original blots for Fig. S2.

## Data Availability

Data sets generated in this study are available as a superseries in the GEO database under accession code GSE189078. Individual series can be obtained as follows: GSE188884 (RNA-seq), GSE189049 (ATAC-seq), and GSE189051 (scRNA-seq). All other data supporting the findings of this study are available at DOI: 10.5281/zenodo.6962483.

## References

[bib1] Åhsberg, J., J. Ungerbäck, T. Strid, E. Welinder, J. Stjernberg, M. Larsson, H. Qian, and M. Sigvardsson. 2013. Early B-cell factor 1 regulates the expansion of B-cell progenitors in a dose-dependent manner. J. Biol. Chem. 288:33449–33461. 10.1074/jbc.M113.506261 24078629 PMC3829190

[bib2] Amemiya, H.M., A. Kundaje, and A.P. Boyle. 2019. The ENCODE blacklist: Identification of problematic regions of the genome. Sci. Rep. 9:9354. 10.1038/s41598-019-45839-z 31249361 PMC6597582

[bib3] Andrews, S. 2010. FastQC: A quality control tool for high throughput sequence data. https://www.bioinformatics.babraham.ac.uk/projects/fastqc/.

[bib4] Bahr, C., L. von Paleske, V.V. Uslu, S. Remeseiro, N. Takayama, S.W. Ng, A. Murison, K. Langenfeld, M. Petretich, R. Scognamiglio, . 2018. A Myc enhancer cluster regulates normal and leukaemic haematopoietic stem cell hierarchies. Nature. 553:515–520. 10.1038/nature25193 29342133

[bib5] Barberi, T., C. Cui, and A.D. Friedman. 2020. C/EBPα induces Ebf1 gene expression in common lymphoid progenitors. PLoS One. 15. e0244161. 10.1371/journal.pone.0244161 33332417 PMC7746190

[bib6] Bentsen, M., P. Goymann, H. Schultheis, K. Klee, A. Petrova, R. Wiegandt, A. Fust, J. Preussner, C. Kuenne, T. Braun, . 2020. ATAC-seq footprinting unravels kinetics of transcription factor binding during zygotic genome activation. Nat. Commun. 11:4267. 10.1038/s41467-020-18035-1 32848148 PMC7449963

[bib7] Bertolino, E., J. Reinitz, and Manu. 2016. The analysis of novel distal Cebpa enhancers and silencers using a transcriptional model reveals the complex regulatory logic of hematopoietic lineage specification. Dev. Biol. 413:128–144. 10.1016/j.ydbio.2016.02.030 26945717 PMC4878123

[bib8] Bhardwaj, V., S. Heyne, K. Sikora, L. Rabbani, M. Rauer, F. Kilpert, A.S. Richter, D.P. Ryan, and T. Manke. 2019. snakePipes: Facilitating flexible, scalable and integrative epigenomic analysis. Bioinformatics. 35:4757–4759. 10.1093/bioinformatics/btz436 31134269 PMC6853707

[bib9] Boller, S., S. Ramamoorthy, D. Akbas, R. Nechanitzky, L. Burger, R. Murr, D. Schubeler, and R. Grosschedl. 2016. Pioneering activity of the C-terminal domain of EBF1 shapes the chromatin landscape for B cell programming. Immunity. 44:527–541. 10.1016/j.immuni.2016.02.021 26982363

[bib10] Boller, S., R. Li, and R. Grosschedl. 2018. Defining B cell chromatin: Lessons from EBF1. Trends Genet. 34:257–269. 10.1016/j.tig.2017.12.014 29336845

[bib11] Bousounis, P., V. Bergo, and E. Trompouki. 2021. Inflammation, aging and hematopoiesis: A complex relationship. Cells. 10:1386. 10.3390/cells10061386 34199874 PMC8227236

[bib12] Buenrostro, J.D., P.G. Giresi, L.C. Zaba, H.Y. Chang, and W.J. Greenleaf. 2013. Transposition of native chromatin for fast and sensitive epigenomic profiling of open chromatin, DNA-binding proteins and nucleosome position. Nat. Methods. 10:1213–1218. 10.1038/nmeth.2688 24097267 PMC3959825

[bib13] Buenrostro, J.D., B. Wu, H.Y. Chang, and W.J. Greenleaf. 2015. ATAC-seq: A method for assaying chromatin accessibility genome-wide. Curr. Protoc. Mol. Biol. 109:21.29.1–21.29.9. 10.1002/0471142727.mb2129s109 PMC4374986 25559105

[bib14] Bussmann, L.H., A. Schubert, T.P. Vu Manh, L. De Andres, S.C. Desbordes, M. Parra, T. Zimmermann, F. Rapino, J. Rodriguez-Ubreva, E. Ballestar, and T. Graf. 2009. A robust and highly efficient immune cell reprogramming system. Cell Stem Cell. 5:554–566. 10.1016/j.stem.2009.10.004 19896445

[bib15] Cabezas-Wallscheid, N., D. Klimmeck, J. Hansson, D.B. Lipka, A. Reyes, Q. Wang, D. Weichenhan, A. Lier, L. von Paleske, S. Renders, . 2014. Identification of regulatory networks in HSCs and their immediate progeny via integrated proteome, transcriptome, and DNA methylome analysis. Cell Stem Cell. 15:507–522. 10.1016/j.stem.2014.07.005 25158935

[bib16] Chavez, J.S., J.L. Rabe, D. Loeffler, K.C. Higa, G. Hernandez, T.S. Mills, N. Ahmed, R.L. Gessner, Z. Ke, B.M. Idler, . 2021. PU.1 enforces quiescence and limits hematopoietic stem cell expansion during inflammatory stress. J. Exp. Med. 218:e20201169. 10.1084/jem.20201169 33857288 PMC8056754

[bib17] Chen, E.Y., C.M. Tan, Y. Kou, Q. Duan, Z. Wang, G.V. Meirelles, N.R. Clark, and A. Ma’ayan. 2013. Enrichr: Interactive and collaborative HTML5 gene list enrichment analysis tool. BMC Bioinformatics. 14:128. 10.1186/1471-2105-14-128 23586463 PMC3637064

[bib18] Cobaleda, C., W. Jochum, and M. Busslinger. 2007. Conversion of mature B cells into T cells by dedifferentiation to uncommitted progenitors. Nature. 449:473–477. 10.1038/nature06159 17851532

[bib19] Cooper, S., H. Guo, and A.D. Friedman. 2015. The +37 kb Cebpa enhancer is critical for Cebpa myeloid gene expression and contains functional sites that bind SCL, GATA2, C/EBPα, PU.1, and additional ets factors. PLoS One. 10:e0126385. 10.1371/journal.pone.0126385 25938608 PMC4418761

[bib20] Corces, M.R., A.E. Trevino, E.G. Hamilton, P.G. Greenside, N.A. Sinnott-Armstrong, S. Vesuna, A.T. Satpathy, A.J. Rubin, K.S. Montine, B. Wu, . 2017. An improved ATAC-seq protocol reduces background and enables interrogation of frozen tissues. Nat. Methods. 14:959–962. 10.1038/nmeth.4396 28846090 PMC5623106

[bib21] Crowell, H.L., C. Soneson, P.L. Germain, D. Calini, L. Collin, C. Raposo, D. Malhotra, and M.D. Robinson. 2020. Muscat detects subpopulation-specific state transitions from multi-sample multi-condition single-cell transcriptomics data. Nat. Commun. 11:6077. 10.1038/s41467-020-19894-4 33257685 PMC7705760

[bib22] Dahl, R., S.R. Iyer, K.S. Owens, D.D. Cuylear, and M.C. Simon. 2007. The transcriptional repressor GFI-1 antagonizes PU.1 activity through protein-protein interaction. J. Biol. Chem. 282:6473–6483. 10.1074/jbc.M607613200 17197705 PMC3218793

[bib23] Dahlin, J.S., F.K. Hamey, B. Pijuan-Sala, M. Shepherd, W.W.Y. Lau, S. Nestorowa, C. Weinreb, S. Wolock, R. Hannah, E. Diamanti, . 2018. A single-cell hematopoietic landscape resolves 8 lineage trajectories and defects in Kit mutant mice. Blood. 131:e1–e11. 10.1182/blood-2017-12-821413 29588278 PMC5969381

[bib24] de Laval, B., J. Maurizio, P.K. Kandalla, G. Brisou, L. Simonnet, C. Huber, G. Gimenez, O. Matcovitch-Natan, S. Reinhardt, E. David, . 2020. C/EBPβ-Dependent epigenetic memory induces trained immunity in hematopoietic stem cells. Cell Stem Cell. 26:657–674.e8. 10.1016/j.stem.2020.01.017 32169166

[bib25] DeKoter, R.P., and H. Singh. 2000. Regulation of B lymphocyte and macrophage development by graded expression of PU.1. Science. 288:1439–1441. 10.1126/science.288.5470.1439 10827957

[bib26] Derecka, M., J.S. Herman, P. Cauchy, S. Ramamoorthy, E. Lupar, D. Grun, and R. Grosschedl. 2020. EBF1-deficient bone marrow stroma elicits persistent changes in HSC potential. Nat. Immunol. 21:261–273. 10.1038/s41590-020-0595-7 32066955

[bib27] Di Tullio, A., T.P. Vu Manh, A. Schubert, G. Castellano, R. Mansson, and T. Graf. 2011. CCAAT/enhancer binding protein alpha (C/EBP(alpha))-induced transdifferentiation of pre-B cells into macrophages involves no overt retrodifferentiation. Proc. Natl. Acad. Sci. USA. 108:17016–17021. 10.1073/pnas.1112169108 21969581 PMC3193237

[bib28] Dias, S., H. Silva Jr, A. Cumano, and P. Vieira. 2005. Interleukin-7 is necessary to maintain the B cell potential in common lymphoid progenitors. J. Exp. Med. 201:971–979. 10.1084/jem.20042393 15767371 PMC2213099

[bib29] Dobin, A., C.A. Davis, F. Schlesinger, J. Drenkow, C. Zaleski, S. Jha, P. Batut, M. Chaisson, and T.R. Gingeras. 2013. STAR: Ultrafast universal RNA-seq aligner. Bioinformatics. 29:15–21. 10.1093/bioinformatics/bts635 23104886 PMC3530905

[bib30] Domen, J., and I.L. Weissman. 1999. Self-renewal, differentiation or death: Regulation and manipulation of hematopoietic stem cell fate. Mol. Med. Today. 5:201–208. 10.1016/S1357-4310(99)01464-1 10322312

[bib31] Dorshkind, K., T. Hofer, E. Montecino-Rodriguez, P.D. Pioli, and H.R. Rodewald. 2020. Do haematopoietic stem cells age?. Nat. Rev. Immunol. 20:196–202. 10.1038/s41577-019-0236-2 31740804 PMC7879798

[bib32] Drissen, R., N. Buza-Vidas, P. Woll, S. Thongjuea, A. Gambardella, A. Giustacchini, E. Mancini, A. Zriwil, M. Lutteropp, A. Grover, . 2016. Distinct myeloid progenitor–differentiation pathways identified through single-cell RNA sequencing. Nat. Immunol. 17:666–676. 10.1038/ni.3412 27043410 PMC4972405

[bib33] Ewels, P., M. Magnusson, S. Lundin, and M. Kaller. 2016. MultiQC: Summarize analysis results for multiple tools and samples in a single report. Bioinformatics. 32:3047–3048. 10.1093/bioinformatics/btw354 27312411 PMC5039924

[bib34] Feng, R., S.C. Desbordes, H. Xie, E.S. Tillo, F. Pixley, E.R. Stanley, and T. Graf. 2008. PU.1 and C/EBPalpha/beta convert fibroblasts into macrophage-like cells. Proc. Natl. Acad. Sci. USA. 105:6057–6062. 10.1073/pnas.0711961105 18424555 PMC2327209

[bib35] Fornes, O., J.A. Castro-Mondragon, A. Khan, R. van der Lee, X. Zhang, P.A. Richmond, B.P. Modi, S. Correard, M. Gheorghe, D. Baranašić, W. Santana-Garcia, . 2020. JASPAR 2020: Update of the open-access database of transcription factor binding profiles. Nucleic Acids Res. 48:D87–D92. 10.1093/nar/gkz1001 31701148 PMC7145627

[bib36] Foudi, A., K. Hochedlinger, D. Van Buren, J.W. Schindler, R. Jaenisch, V. Carey, and H. Hock. 2009. Analysis of histone 2B-GFP retention reveals slowly cycling hematopoietic stem cells. Nat. Biotechnol. 27:84–90. 10.1038/nbt.1517 19060879 PMC2805441

[bib37] Fukuchi, Y., F. Shibata, M. Ito, Y. Goto-Koshino, Y. Sotomaru, M. Ito, T. Kitamura, and H. Nakajima. 2006. Comprehensive analysis of myeloid lineage conversion using mice expressing an inducible form of C/EBP alpha. EMBO J. 25:3398–3410. 10.1038/sj.emboj.7601199 16858416 PMC1523173

[bib38] Gekas, C., and T. Graf. 2013. CD41 expression marks myeloid-biased adult hematopoietic stem cells and increases with age. Blood. 121:4463–4472. 10.1182/blood-2012-09-457929 23564910

[bib39] Guo, H., S. Cooper, and A.D. Friedman. 2016. In vivo deletion of the Cebpa +37 kb enhancer markedly reduces Cebpa mRNA in myeloid progenitors but not in non-hematopoietic tissues to impair granulopoiesis. PLoS One. 11:e0150809. 10.1371/journal.pone.0150809 26937964 PMC4777376

[bib40] Guo, H., T. Barberi, R. Suresh, and A.D. Friedman. 2018. Progression from the common lymphoid progenitor to B/myeloid PreproB and ProB precursors during B lymphopoiesis requires C/EBPα. J. Immunol. 201:1692–1704. 10.4049/jimmunol.1800244 30061199 PMC6125169

[bib41] Györy, I., S. Boller, R. Nechanitzky, E. Mandel, S. Pott, E. Liu, and R. Grosschedl. 2012. Transcription factor Ebf1 regulates differentiation stage-specific signaling, proliferation, and survival of B cells. Genes Dev. 26:668–682. 10.1101/gad.187328.112 22431510 PMC3323878

[bib42] Hagman, J., J. Ramírez, and K. Lukin. 2011. B lymphocyte lineage specification, commitment and epigenetic control of transcription by early B cell factor 1. In Epigenetic Regulation of Lymphocyte Development. C. Murre, editor. Springer Berlin Heidelberg (Current Topics in Microbiology and Immunology), Berlin, Heidelberg. 17–38. 10.1007/82_2011_139 PMC3925327 21735360

[bib43] Hamey, F.K., and B. Göttgens. 2019. Machine learning predicts putative hematopoietic stem cells within large single-cell transcriptomics data sets. Exp. Hematol. 78:11–20. 10.1016/j.exphem.2019.08.009 31513832 PMC6900257

[bib44] Hasemann, M.S., F.K. Lauridsen, J. Waage, J.S. Jakobsen, A.K. Frank, M.B. Schuster, N. Rapin, F.O. Bagger, P.S. Hoppe, T. Schroeder, and B.T. Porse. 2014. C/EBPα is required for long-term self-renewal and lineage priming of hematopoietic stem cells and for the maintenance of epigenetic configurations in multipotent progenitors. PLoS Genet. 10. e1004079. 10.1371/journal.pgen.1004079 24415956 PMC3886906

[bib45] Heinz, S., C. Benner, N. Spann, E. Bertolino, Y.C. Lin, P. Laslo, J.X. Cheng, C. Murre, H. Singh, and C.K. Glass. 2010. Simple combinations of lineage-determining transcription factors prime cis-regulatory elements required for macrophage and B cell identities. Mol. Cell. 38:576–589. 10.1016/j.molcel.2010.05.004 20513432 PMC2898526

[bib46] Hormaechea-Agulla, D., D.T. Le, and K.Y. King. 2020. Common sources of inflammation and their impact on hematopoietic stem cell biology. Curr. Stem Cell Rep. 6:96–107. 10.1007/s40778-020-00177-z 32837857 PMC7429415

[bib47] Hu, M., D. Krause, M. Greaves, S. Sharkis, M. Dexter, C. Heyworth, and T. Enver. 1997. Multilineage gene expression precedes commitment in the hemopoietic system. Genes Dev. 11:774–785. 10.1101/gad.11.6.774 9087431

[bib48] Inlay, M.A., D. Bhattacharya, D. Sahoo, T. Serwold, J. Seita, H. Karsunky, S.K. Plevritis, D.L. Dill, and I.L. Weissman. 2009. Ly6d marks the earliest stage of B-cell specification and identifies the branchpoint between B-cell and T-cell development. Genes Dev. 23:2376–2381. 10.1101/gad.1836009 19833765 PMC2764492

[bib49] Iwasaki, H., C. Somoza, H. Shigematsu, E.A. Duprez, J. Iwasaki-Arai, S.I. Mizuno, Y. Arinobu, K. Geary, P. Zhang, T. Dayaram, . 2005. Distinctive and indispensable roles of PU.1 in maintenance of hematopoietic stem cells and their differentiation. Blood. 106:1590–1600. 10.1182/blood-2005-03-0860 15914556 PMC1895212

[bib50] Jaffredo, T., R. Gautier, A. Eichmann, and F. Dieterlen-Lievre. 1998. Intraaortic hemopoietic cells are derived from endothelial cells during ontogeny. Development. 125:4575–4583. 10.1242/dev.125.22.4575 9778515

[bib51] Jensen, C.T., J. Ahsberg, M.N.E. Sommarin, T. Strid, R. Somasundaram, K. Okuyama, J. Ungerback, J. Kupari, M.S. Airaksinen, S. Lang, . 2018. Dissection of progenitor compartments resolves developmental trajectories in B-lymphopoiesis. J. Exp. Med. 215:1947–1963. 10.1084/jem.20171384 29899037 PMC6028518

[bib52] Kang, Y.-A., H. Paik, S.Y. Zhang, J. Chen, M.R. Warr, R. Fan, and E. Passegué. 2021. Secretory MPP3 reinforce myeloid differentiation trajectory and amplify myeloid cell production. bioRxiv. 10.1101/2021.09.01.458573 PMC10140385 37115584

[bib53] Kikuchi, K., A.Y. Lai, C.L. Hsu, and M. Kondo. 2005. IL-7 receptor signaling is necessary for stage transition in adult B cell development through up-regulation of EBF. J. Exp. Med. 201:1197–1203. 10.1084/jem.20050158 15837809 PMC2213146

[bib54] Kisanuki, Y.Y., R.E. Hammer, J. Miyazaki, S.C. Williams, J.A. Richardson, and M. Yanagisawa. 2001. Tie2-Cre transgenic mice: A new model for endothelial cell-lineage analysis in vivo. Dev. Biol. 230:230–242. 10.1006/dbio.2000.0106 11161575

[bib55] Kitamura, D., J. Roes, R. Kuhn, and K. Rajewsky. 1991. A B cell-deficient mouse by targeted disruption of the membrane exon of the immunoglobulin μ chain gene. Nature. 350:423–426. 10.1038/350423a0 1901381

[bib56] Kolde, R. 2019. Pheatmap: Pretty heatmaps. https://rdrr.io/cran/pheatmap/

[bib57] Kondili, M., A. Fust, J. Preussner, C. Kuenne, T. Braun, and M. Looso. 2017. UROPA: A tool for universal RObust peak annotation. Sci. Rep. 7:2593. 10.1038/s41598-017-02464-y 28572580 PMC5453960

[bib58] Korsunsky, I., J. Fan, K. Slowikowski, F. Zhang, K. Wei, Y. Baglaenko, M. Brenner, P.-R. Loh, and S. Raychaudhuri. 2018. Fast, sensitive, and accurate integration of single cell data with Harmony. bioRxiv. (Preprint posted November 05, 2018). 10.1101/461954 PMC6884693 31740819

[bib59] Koster, J., and S. Rahmann. 2012. Snakemake: A scalable bioinformatics workflow engine. Bioinformatics. 28:2520–2522. 10.1093/bioinformatics/bts480 22908215

[bib60] Kucinski, I., N.K. Wilson, R. Hannah, S.J. Kinston, P. Cauchy, A. Lenaerts, R. Grosschedl, and B. Gottgens. 2020. Interactions between lineage-associated transcription factors govern haematopoietic progenitor states. EMBO J. 39:e104983. 10.15252/embj.2020104983 33103827 PMC7737608

[bib61] Kuleshov, M.V., M.R. Jones, A.D. Rouillard, N.F. Fernandez, Q. Duan, Z. Wang, S. Koplev, S.L. Jenkins, K.M. Jagodnik, A. Lachmann, . 2016. Enrichr: A comprehensive gene set enrichment analysis web server 2016 update. Nucleic Acids Res. 44:W90–W97. 10.1093/nar/gkw377 27141961 PMC4987924

[bib62] Laiosa, C.V., M. Stadtfeld, and T. Graf. 2006. Determinants of lymphoid-myeloid lineage diversification. Annu. Rev. Immunol. 24:705–738. 10.1146/annurev.immunol.24.021605.090742 16551264

[bib63] Lara-Astiaso, D., A. Weiner, E. Lorenzo-Vivas, I. Zaretsky, D.A. Jaitin, E. David, H. Keren-Shaul, A. Mildner, D. Winter, S. Jung, . 2014. Immunogenetics. Chromatin state dynamics during blood formation. Science. 345:943–949. 10.1126/science.1256271 25103404 PMC4412442

[bib64] Laslo, P., C.J. Spooner, A. Warmflash, D.W. Lancki, H.J. Lee, R. Sciammas, B.N. Gantner, A.R. Dinner, and H. Singh. 2006. Multilineage transcriptional priming and determination of alternate hematopoietic cell fates. Cell. 126:755–766. 10.1016/j.cell.2006.06.052 16923394

[bib65] Lauridsen, F.K.B., T.L. Jensen, N. Rapin, D. Aslan, A.S. Wilhelmson, S. Pundhir, M. Rehn, F. Paul, A. Giladi, M.S. Hasemann, . 2018. Differences in cell cycle status underlie transcriptional heterogeneity in the HSC compartment. Cell Rep. 24:766–780. 10.1016/j.celrep.2018.06.057 30021172

[bib66] Lee, J., S.R. Yoon, I. Choi, and H. Jung. 2019. Causes and mechanisms of hematopoietic stem cell aging. Int. J. Mol. Sci. 20:1272. 10.3390/ijms20061272 PMC6470724 30871268

[bib67] Lescale, C., S. Dias, J. Maes, A. Cumano, P. Szabo, D. Charron, M.E. Weksler, C. Dosquet, P. Vieira, and M. Goodhardt. 2010. Reduced EBF expression underlies loss of B-cell potential of hematopoietic progenitors with age: Reduced expression of EBF in common lymphoid progenitors (CLP) with age. Aging Cell. 9:410–419. 10.1111/j.1474-9726.2010.00566.x 20331442

[bib68] Lescale, C., V. Schenten, D. Djeghloul, M. Bennabi, F. Gaignier, K. Vandamme, C. Strazielle, I. Kuzniak, H. Petite, C. Dosquet, . 2015. Hind limb unloading, a model of spaceflight conditions, leads to decreased B lymphopoiesis similar to aging. FASEB J. 29:455–463. 10.1096/fj.14-259770 25376832

[bib69] Li, R., P. Cauchy, S. Ramamoorthy, S. Boller, L. Chavez, and R. Grosschedl. 2018. Dynamic EBF1 occupancy directs sequential epigenetic and transcriptional events in B-cell programming. Genes Dev. 32:96–111. 10.1101/gad.309583.117 29440261 PMC5830932

[bib70] Liao, Y., G.K. Smyth, and W. Shi. 2014. featureCounts: An efficient general purpose program for assigning sequence reads to genomic features. Bioinformatics. 30:923–930. 10.1093/bioinformatics/btt656 24227677

[bib71] Lin, H., and R. Grosschedl. 1995. Failure of B-cell differentiation in mice lacking the transcription factor EBF. Nature. 376:263–267. 10.1038/376263a0 7542362

[bib72] Lin, Y.C., S. Jhunjhunwala, C. Benner, S. Heinz, E. Welinder, R. Mansson, M. Sigvardsson, J. Hagman, C.A. Espinoza, J. Dutkowski, . 2010. A global network of transcription factors, involving E2A, EBF1 and Foxo1, that orchestrates B cell fate. Nat. Immunol. 11:635–643. 10.1038/ni.1891 20543837 PMC2896911

[bib73] Love, M.I., W. Huber, and S. Anders. 2014. Moderated estimation of fold change and dispersion for RNA-seq data with DESeq2. Genome Biol. 15:550. 10.1186/s13059-014-0550-8 25516281 PMC4302049

[bib74] Lukin, K., S. Fields, L Guerrettaz, D. Straign, V. Rodriguez, S. Zandi, R. Mansson, J.C. Cambier, M. Sigvardsson, and J. Hagman. 2011. A dose-dependent role for EBF1 in repressing non-B-cell-specific genes. Eur. J. Immunol. 41:1787–1793. 10.1002/eji.201041137 21469119 PMC3127254

[bib75] Lun, A.T.L., and J.C. Marioni. 2017. Overcoming confounding plate effects in differential expression analyses of single-cell RNA-seq data. Biostatistics. 18:451–464. 10.1093/biostatistics/kxw055 28334062 PMC5862359

[bib76] Macosko, E.Z., A. Basu, R. Satija, J. Nemesh, K. Shekhar, M. Goldman, I. Tirosh, A.R. Bialas, N. Kamitaki, E.M. Martersteck, . 2015. Highly parallel genome-wide expression profiling of individual cells using nanoliter droplets. Cell. 161:1202–1214. 10.1016/j.cell.2015.05.002 26000488 PMC4481139

[bib77] Mansson, R., E. Welinder, J. Ahsberg, Y.C. Lin, C. Benner, C.K. Glass, J.S. Lucas, M. Sigvardsson, and C. Murre. 2012. Positive intergenic feedback circuitry, involving EBF1 and FOXO1, orchestrates B-cell fate. Proc. Natl. Acad. Sci. USA. 109:21028–21033. 10.1073/pnas.1211427109 23213261 PMC3529039

[bib78] Martin, M. 2011. Cutadapt removes adapter sequences from high-throughput sequencing reads. EMBnet.Journal. 17:10. 10.14806/ej.17.1.200

[bib79] McCabe, A., and K.C. MacNamara. 2016. Macrophages: Key regulators of steady-state and demand-adapted hematopoiesis. Exp. Hematol. 44:213–222. 10.1016/j.exphem.2016.01.003 26806720 PMC4852701

[bib80] McInnes, L., J. Healy and J. Melville 2020. UMAP: Uniform manifold approximation and projection for dimension reduction. arXiv. (Accessed November 16, 2021). 10.48550/arXiv.1802.03426

[bib81] McLeay, R.C., and T.L. Bailey. 2010. Motif enrichment analysis: A unified framework and an evaluation on ChIP data. BMC Bioinf. 11:165. 10.1186/1471-2105-11-165 PMC2868005 20356413

[bib82] Medina, K.L., J.M.R. Pongubala, K.L. Reddy, D.W. Lancki, R. Dekoter, M. Kieslinger, R. Grosschedl, and H. Singh. 2004. Assembling a gene regulatory network for specification of the B cell fate. Dev. Cell. 7:607–617. 10.1016/j.devcel.2004.08.006 15469848

[bib83] Mejia-Ramirez, E., and M.C. Florian. 2020. Understanding intrinsic hematopoietic stem cell aging. Haematologica. 105:22–37. 10.3324/haematol.2018.211342 31806687 PMC6939535

[bib84] Mercer, E.M., Y.C. Lin, C. Benner, S. Jhunjhunwala, J. Dutkowski, M. Flores, M. Sigvardsson, T. Ideker, C.K. Glass, and C. Murre. 2011. Multilineage priming of enhancer repertoires precedes commitment to the B and myeloid cell lineages in hematopoietic progenitors. Immunity. 35:413–425. 10.1016/j.immuni.2011.06.013 21903424 PMC3183365

[bib85] Mikkola, I., B. Heavey, M. Horcher, and M. Busslinger. 2002. Reversion of B cell commitment upon loss of Pax5 expression. Science. 297:110–113. 10.1126/science.1067518 12098702

[bib86] Mirantes, C., E. Passegué, and E.M. Pietras. 2014. Pro-inflammatory cytokines: Emerging players regulating HSC function in normal and diseased hematopoiesis. Exp. Cell Res. 329:248–254. 10.1016/j.yexcr.2014.08.017 25149680 PMC4250307

[bib87] Mitroulis, I., L. Kalafati, G. Hajishengallis, and T. Chavakis. 2018. Myelopoiesis in the context of innate immunity. J. Innate Immun. 10:365–372. 10.1159/000489406 29874678 PMC6281852

[bib88] Miyamoto, T., H. Iwasaki, B. Reizis, M. Ye, T. Graf, I.L. Weissman, and K. Akashi. 2002. Myeloid or lymphoid promiscuity as a critical step in hematopoietic lineage commitment. Dev. Cell. 3:137–147. 10.1016/S1534-5807(02)00201-0 12110174

[bib89] Miyawaki, K., Y. Arinobu, H. Iwasaki, K. Kohno, H. Tsuzuki, T. Iino, T. Shima, Y. Kikushige, K. Takenaka, T. Miyamoto, and K. Akashi. 2015. CD41 marks the initial myelo-erythroid lineage specification in adult mouse hematopoiesis: Redefinition of murine common myeloid progenitor. Stem Cell. 33:976–987. 10.1002/stem.1906 25446279

[bib90] Mootha, V.K., C.M. Lindgren, K.F. Eriksson, A. Subramanian, S. Sihag, J. Lehar, P. Puigserver, E. Carlsson, M. Ridderstrale, E. Laurila, . 2003. PGC-1alpha-responsive genes involved in oxidative phosphorylation are coordinately downregulated in human diabetes. Nat. Genet. 34:267–273. 10.1038/ng1180 12808457

[bib91] Nechanitzky, R., D. Akbas, S. Scherer, I. Gyory, T. Hoyler, S. Ramamoorthy, A. Diefenbach, and R. Grosschedl. 2013. Transcription factor EBF1 is essential for the maintenance of B cell identity and prevention of alternative fates in committed cells. Nat. Immunol. 14:867–875. 10.1038/ni.2641 23812095

[bib92] Nestorowa, S., F.K. Hamey, B. Pijuan Sala, E. Diamanti, M. Shepherd, E. Laurenti, N.K. Wilson, D.G. Kent, and B. Gottgens. 2016. A single-cell resolution map of mouse hematopoietic stem and progenitor cell differentiation. Blood. 128:e20–e31. 10.1182/blood-2016-05-716480 27365425 PMC5305050

[bib93] Nimmo, R.A., G.E. May, and T. Enver. 2015. Primed and ready: Understanding lineage commitment through single cell analysis. Trends Cell Biol. 25:459–467. 10.1016/j.tcb.2015.04.004 26004869

[bib94] Orkin, S.H., and L.I. Zon. 2008. Hematopoiesis: An evolving paradigm for stem cell biology. Cell. 132:631–644. 10.1016/j.cell.2008.01.025 18295580 PMC2628169

[bib95] O’Riordan, M., and R. Grosschedl. 1999. Coordinate regulation of B cell differentiation by the transcription factors EBF and E2A. Immunity. 11:21–31. 10.1016/S1074-7613(00)80078-3 10435576

[bib96] Palii, C.G., Q. Cheng, M.A. Gillespie, P. Shannon, M. Mazurczyk, G. Napolitani, N.D. Price, J.A. Ranish, E. Morrissey, D.R. Higgs, and M. Brand. 2019. Single-cell proteomics reveal that quantitative changes in Co-expressed lineage-specific transcription factors determine cell fate. Cell Stem Cell. 24:812–820.e5. 10.1016/j.stem.2019.02.006 30880026 PMC6886472

[bib97] Pang, S.H.M., C.A. de Graaf, D.J. Hilton, N.D. Huntington, S. Carotta, L. Wu, and S.L. Nutt. 2018. PU.1 is required for the developmental progression of multipotent progenitors to common lymphoid progenitors. Front. Immunol. 9:1264. 10.3389/fimmu.2018.01264 29942304 PMC6005176

[bib98] Passegué, E., A.J. Wagers, S. Giuriato, W.C. Anderson, and I.L. Weissman. 2005. Global analysis of proliferation and cell cycle gene expression in the regulation of hematopoietic stem and progenitor cell fates. J. Exp. Med. 202:1599–1611. 10.1084/jem.20050967 16330818 PMC2213324

[bib99] Paul, F., Y. Arkin, A. Giladi, D.A. Jaitin, E. Kenigsberg, H. Keren-Shaul, D. Winter, D. Lara-Astiaso, M. Gury, A. Weiner, . 2015. Transcriptional heterogeneity and lineage commitment in myeloid progenitors. Cell. 163:1663–1677. 10.1016/j.cell.2015.11.013 26627738

[bib100] Pietras, E.M., D. Reynaud, Y.A. Kang, D. Carlin, F.J. Calero-Nieto, A.D. Leavitt, J.M. Stuart, B. Gottgens, and E. Passegue. 2015. Functionally distinct subsets of lineage-biased multipotent progenitors control blood production in normal and regenerative conditions. Cell Stem Cell. 17:35–46. 10.1016/j.stem.2015.05.003 26095048 PMC4542150

[bib101] Polański, K., M.D. Young, Z. Miao, K.B. Meyer, S.A. Teichmann, and J.E. Park. 2020. BBKNN: Fast batch alignment of single cell transcriptomes. Bioinformatics. 36:964–965. 10.1093/bioinformatics/btz625 31400197 PMC9883685

[bib102] Pongubala, J.M.R., D.L. Northrup, D.W. Lancki, K.L. Medina, T. Treiber, E. Bertolino, M. Thomas, R. Grosschedl, D. Allman, and H. Singh. 2008. Transcription factor EBF restricts alternative lineage options and promotes B cell fate commitment independently of Pax5. Nat. Immunol. 9:203–215. 10.1038/ni1555 18176567

[bib103] Popescu, D.-M., R.A. Botting, E. Stephenson, K. Green, S. Webb, L. Jardine, E.F. Calderbank, K. Polanski, I. Goh, M. Efremova, . 2019. Decoding human fetal liver haematopoiesis. Nature. 574:365–371. 10.1038/s41586-019-1652-y 31597962 PMC6861135

[bib104] Pronk, C.J.H., D.J. Rossi, R. Mansson, J.L. Attema, G.L. Norddahl, C.K.F. Chan, M. Sigvardsson, I.L. Weissman, and D. Bryder. 2007. Elucidation of the phenotypic, functional, and molecular topography of a myeloerythroid progenitor cell hierarchy. Cell Stem Cell. 1:428–442. 10.1016/j.stem.2007.07.005 18371379

[bib105] Pundhir, S., F.K. Bratt Lauridsen, M.B. Schuster, J.S. Jakobsen, Y. Ge, E.M. Schoof, N. Rapin, J. Waage, M.S. Hasemann, and B.T. Porse. 2018. Enhancer and transcription factor dynamics during myeloid differentiation reveal an early differentiation block in Cebpa null progenitors. Cell Rep. 23:2744–2757. 10.1016/j.celrep.2018.05.012 29847803

[bib106] Quinlan, A.R., and I.M. Hall. 2010. BEDTools: A flexible suite of utilities for comparing genomic features. Bioinformatics. 26:841–842. 10.1093/bioinformatics/btq033 20110278 PMC2832824

[bib107] R Core Team. 2020. R: A language and environment for statistical computing. R Foundation for Statistical Computing, Vienna, Austria. https://www.R-project.org/

[bib108] Ramamoorthy, S., K. Kometani, J.S. Herman, M. Bayer, S. Boller, J. Edwards-Hicks, H. Ramachandran, R. Li, R. Klein-Geltink, E.L. Pearce, . 2020. EBF1 and Pax5 safeguard leukemic transformation by limiting IL-7 signaling, Myc expression, and folate metabolism. Genes Dev. 34:1503–1519. 10.1101/gad.340216.120 33004416 PMC7608749

[bib109] Ramírez, F., D.P. Ryan, B. Gruning, V. Bhardwaj, F. Kilpert, A.S. Richter, S. Heyne, F. Dundar, and T. Manke. 2016. deepTools2: A next generation web server for deep-sequencing data analysis. Nucleic Acids Res. 44:W160–W165. 10.1093/nar/gkw257 27079975 PMC4987876

[bib110] Ramírez, F., V. Bhardwaj, L. Arrigoni, K.C. Lam, B.A. Gruning, J. Villaveces, B. Habermann, A. Akhtar, and T. Manke. 2018. High-resolution TADs reveal DNA sequences underlying genome organization in flies. Nat. Commun. 9:189. 10.1038/s41467-017-02525-w 29335486 PMC5768762

[bib111] Ramisch, A., V. Heinrich, L.V. Glaser, A. Fuchs, X. Yang, P. Benner, R. Schopflin, N. Li, S. Kinkley, A. Romer-Hillmann, . 2019. CRUP: A comprehensive framework to predict condition-specific regulatory units. Genome Biol. 20:227. 10.1186/s13059-019-1860-7 31699133 PMC6839171

[bib112] Ranzoni, A.M., A. Tangherloni, I. Berest, S.G. Riva, B. Myers, P.M. Strzelecka, J. Xu, E. Panada, I. Mohorianu, J.B. Zaugg, and A. Cvejic. 2021. Integrative single-cell RNA-seq and ATAC-seq analysis of human developmental hematopoiesis. Cell Stem Cell. 28:472–487.e7. 10.1016/j.stem.2020.11.015 33352111 PMC7939551

[bib113] Redecke, V., R. Wu, J. Zhou, D. Finkelstein, V. Chaturvedi, A.A. High, and H. Hacker. 2013. Hematopoietic progenitor cell lines with myeloid and lymphoid potential. Nat. Methods. 10:795–803. 10.1038/nmeth.2510 23749299 PMC4131762

[bib114] Repele, A., S. Krueger, T. Bhattacharyya, M.Y. Tuineau, and Manu. 2019. The regulatory control of Cebpa enhancers and silencers in the myeloid and red-blood cell lineages. PLoS One. 14:e0217580. 10.1371/journal.pone.0217580 31181110 PMC6557489

[bib115] Reynaud, D., I.A. Demarco, K.L. Reddy, H. Schjerven, E. Bertolino, Z. Chen, S.T. Smale, S. Winandy, and H. Singh. 2008. Regulation of B cell fate commitment and immunoglobulin heavy-chain gene rearrangements by Ikaros. Nat. Immunol. 9:927–936. 10.1038/ni.1626 18568028 PMC2699484

[bib116] Riley, R.L. 2013. Impaired B lymphopoiesis in old age: A role for inflammatory B cells?. Immunol. Res. 57:361–369. 10.1007/s12026-013-8444-5 24203438 PMC3972803

[bib117] Robinson, M.D., D.J. McCarthy, and G.K. Smyth. 2010. edgeR: A Bioconductor package for differential expression analysis of digital gene expression data. Bioinformatics. 26:139–140. 10.1093/bioinformatics/btp616 19910308 PMC2796818

[bib118] Rodriguez-Fraticelli, A.E., S.L. Wolock, C.S. Weinreb, R. Panero, S.H. Patel, M. Jankovic, J. Sun, R.A. Calogero, A.M. Klein, and F.D. Camargo. 2018. Clonal analysis of lineage fate in native haematopoiesis. Nature. 553:212–216. 10.1038/nature25168 29323290 PMC5884107

[bib150] Roessler, S., I. Györy, S. Imhof, M. Spivakov, R.R. Williams, M. Busslinger, A.G. Fisher, and R. Grosschedl. 2007. Distinct promoters mediate the regulation of Ebf1 gene expression by interleukin-7 and Pax5. Mol. Cell Biol. 27:579–594. 10.1128/MCB.01192-06 17101802 PMC1800812

[bib119] Seet, C.S., R.L. Brumbaugh, and B.L. Kee. 2004. Early B cell factor promotes B lymphopoiesis with reduced interleukin 7 responsiveness in the absence of E2A. J. Exp. Med. 199:1689–1700. 10.1084/jem.20032202 15210745 PMC2212815

[bib120] Snoeck, H.-W. 2013. Aging of the hematopoietic system. Curr. Opin. Hematol. 20:355–361. 10.1097/MOH.0b013e3283623c77 23739721

[bib121] Somasundaram, R., C.T. Jensen, J. Tingvall-Gustafsson, J. Ahsberg, K. Okuyama, M. Prasad, J.R. Hagman, X. Wang, S. Soneji, T. Strid, . 2021. EBF1 and PAX5 control pro-B cell expansion via opposing regulation of the *Myc* gene. Blood. 137:3037–3049. 10.1182/blood.2020009564 33619557 PMC8176764

[bib122] Sommerkamp, P., S. Altamura, S. Renders, A. Narr, L. Ladel, P. Zeisberger, P.L. Eiben, M. Fawaz, M.A. Rieger, N. Cabezas-Wallscheid, and A. Trumpp. 2020. Differential alternative polyadenylation landscapes mediate hematopoietic stem cell activation and regulate glutamine metabolism. Cell Stem Cell. 26:722–738.e7. 10.1016/j.stem.2020.03.003 32229311

[bib123] Sommerkamp, P., M.C. Romero-Mulero, A. Narr, L. Ladel, L. Hustin, K. Schonberger, S. Renders, S. Altamura, P. Zeisberger, K. Jacklein, . 2021. Mouse multipotent progenitor 5 cells are located at the interphase between hematopoietic stem and progenitor cells. Blood. 137:3218–3224. 10.1182/blood.2020007876 33754628 PMC8351880

[bib124] Spooner, C.J., J.X. Cheng, E. Pujadas, P. Laslo, and H. Singh. 2009. A recurrent network involving the transcription factors PU.1 and Gfi1 orchestrates innate and adaptive immune cell fates. Immunity. 31:576–586. 10.1016/j.immuni.2009.07.011 19818654 PMC4373467

[bib125] Stephens, M. 2017. False discovery rates: A new deal. Biostatistics. 18:275–294. 10.1093/biostatistics/kxw041 27756721 PMC5379932

[bib126] Stuart, T., A. Butler, P. Hoffman, C. Hafemeister, E. Papalexi, W.M. Mauck 3rd, Y. Hao, M. Stoeckius, P. Smibert, and R. Satija. 2019. Comprehensive integration of single-cell data. Cell. 177:1888–1902.e21. 10.1016/j.cell.2019.05.031 31178118 PMC6687398

[bib127] Subramanian, A., P. Tamayo, V.K. Mootha, S. Mukherjee, B.L. Ebert, M.A. Gillette, A. Paulovich, S.L. Pomeroy, T.R. Golub, E.S. Lander, and J.P. Mesirov. 2005. Gene set enrichment analysis: A knowledge-based approach for interpreting genome-wide expression profiles. Proc. Natl. Acad. Sci. USA. 102:15545–15550. 10.1073/pnas.0506580102 16199517 PMC1239896

[bib128] Traag, V.A., L. Waltman, and N.J. van Eck. 2019. From louvain to leiden: Guaranteeing well-connected communities. Sci. Rep. 9:5233. 10.1038/s41598-019-41695-z 30914743 PMC6435756

[bib129] Treiber, T., E.M. Mandel, S. Pott, I. Gyory, S. Firner, E.T. Liu, and R. Grosschedl. 2010. Early B cell factor 1 regulates B cell gene networks by activation, repression, and transcription- independent poising of chromatin. Immunity. 32:714–725. 10.1016/j.immuni.2010.04.013 20451411

[bib130] van Galen, P., A. Kreso, E. Wienholds, E. Laurenti, K. Eppert, E.R. Lechman, N. Mbong, K. Hermans, S. Dobson, C. April, . 2014. Reduced lymphoid lineage priming promotes human hematopoietic stem cell expansion. Cell Stem Cell. 14:94–106. 10.1016/j.stem.2013.11.021 24388174

[bib131] Wang, Y., N. Zolotarev, C.Y. Yang, A. Rambold, G. Mittler, and R. Grosschedl. 2020. A prion-like domain in transcription factor EBF1 promotes phase separation and enables B cell programming of progenitor chromatin. Immunity. 53:1151–1167.e6. 10.1016/j.immuni.2020.10.009 33159853

[bib132] Weinreb, C., A. Rodriguez-Fraticelli, F.D. Camargo, and A.M. Klein. 2020. Lineage tracing on transcriptional landscapes links state to fate during differentiation. Science. 367:eaaw3381. 10.1126/science.aaw3381 31974159 PMC7608074

[bib133] Welinder, E., R. Mansson, E.M. Mercer, D. Bryder, M. Sigvardsson, and C. Murre. 2011. The transcription factors E2A and HEB act in concert to induce the expression of FOXO1 in the common lymphoid progenitor. Proc. Natl. Acad. Sci. USA. 108:17402–17407. 10.1073/pnas.1111766108 21972416 PMC3198373

[bib134] Wickham, H. 2016. ggplot2: Elegant Graphics for Data Analysis. 2nd ed. Springer International Publishing (Use R!), Cham. 10.1007/978-3-319-24277-4

[bib135] Wilson, N.K., D.G. Kent, F. Buettner, M. Shehata, I.C. Macaulay, F.J. Calero-Nieto, M. Sanchez Castillo, C.A. Oedekoven, E. Diamanti, R. Schulte, . 2015. Combined single-cell functional and gene expression analysis resolves heterogeneity within stem cell populations. Cell Stem Cell. 16:712–724. 10.1016/j.stem.2015.04.004 26004780 PMC4460190

[bib136] Winandy, S., P. Wu, and K. Georgopoulos. 1995. A dominant mutation in the Ikaros gene leads to rapid development of leukemia and lymphoma. Cell. 83:289–299. 10.1016/0092-8674(95)90170-1 7585946

[bib137] Wolf, F.A., P. Angerer, and F.J. Theis. 2018. SCANPY: Large-scale single-cell gene expression data analysis. Genome Biol. 19:15. 10.1186/s13059-017-1382-0 29409532 PMC5802054

[bib138] Wu, T., E. Hu, S. Xu, M. Chen, P. Guo, Z. Dai, T. Feng, L. Zhou, W. Tang, L. Zhan, . 2021. clusterProfiler 4.0: A universal enrichment tool for interpreting omics data. Innovation. 2:100141. 10.1016/j.xinn.2021.100141 34557778 PMC8454663

[bib139] Xie, H., M. Ye, R. Feng, and T. Graf. 2004. Stepwise reprogramming of B cells into macrophages. Cell. 117:663–676. 10.1016/S0092-8674(04)00419-2 15163413

[bib140] Yamamoto, R., Y. Morita, J. Ooehara, S. Hamanaka, M. Onodera, K.L. Rudolph, H. Ema, and H. Nakauchi. 2013. Clonal analysis unveils self-renewing lineage-restricted progenitors generated directly from hematopoietic stem cells. Cell. 154:1112–1126. 10.1016/j.cell.2013.08.007 23993099

[bib141] Ye, M., H. Zhang, G. Amabile, H. Yang, P.B. Staber, P. Zhang, E. Levantini, M. Alberich-Jorda, J. Zhang, A. Kawasaki, and D.G. Tenen. 2013. C/EBPa controls acquisition and maintenance of adult haematopoietic stem cell quiescence. Nat. Cell Biol. 15:385–394. 10.1038/ncb2698 23502316 PMC3781213

[bib142] Ye, M., H. Zhang, H. Yang, R. Koche, P.B. Staber, M. Cusan, E. Levantini, R.S. Welner, C.S. Bach, J. Zhang, . 2015. Hematopoietic differentiation is required for initiation of acute myeloid leukemia. Cell Stem Cell. 17:611–623. 10.1016/j.stem.2015.08.011 26412561 PMC4636971

[bib143] Yoshida, T., S.Y. Ng, J.C. Zuniga-Pflucker, and K. Georgopoulos. 2006. Early hematopoietic lineage restrictions directed by Ikaros. Nat. Immunol. 7:382–391. 10.1038/ni1314 16518393 PMC3872276

[bib144] Zarnegar, M.A., and E.V. Rothenberg. 2012. Ikaros represses and activates PU.1 cell-type-specifically through the multifunctional Sfpi1 URE and a myeloid specific enhancer. Oncogene. 31:4647–4654. 10.1038/onc.2011.597 22231443 PMC3679182

[bib145] Zerbino, D.R., N. Johnson, T. Juettemann, S.P. Wilder, and P. Flicek. 2014. WiggleTools: Parallel processing of large collections of genome-wide datasets for visualization and statistical analysis. Bioinformatics. 30:1008–1009. 10.1093/bioinformatics/btt737 24363377 PMC3967112

[bib146] Zhang, D.-E., P. Zhang, N.D. Wang, C.J. Hetherington, G.J. Darlington, and D.G. Tenen. 1997. Absence of granulocyte colony-stimulating factor signaling and neutrophil development in CCAAT enhancer binding protein -deficient mice. Proc. Natl. Acad. Sci. USA. 94:569–574. 10.1073/pnas.94.2.569 9012825 PMC19554

[bib147] Zhang, Z., C.V. Cotta, R.P. Stephan, C.G. deGuzman, and C.A. Klug. 2003. Enforced expression of EBF in hematopoietic stem cells restricts lymphopoiesis to the B cell lineage. EMBO J. 22:4759–4769. 10.1093/emboj/cdg464 12970188 PMC212730

[bib148] Zhang, Y., T. Liu, C.A. Meyer, J. Eeckhoute, D.S. Johnson, B.E. Bernstein, C. Nusbaum, R.M. Myers, M. Brown, W. Li, and X.S. Liu. 2008. Model-based analysis of ChIP-seq (MACS). Genome Biol. 9:R137. 10.1186/gb-2008-9-9-r137 18798982 PMC2592715

[bib149] Zovein, A.C., J.J. Hofmann, M. Lynch, W.J. French, K.A. Turlo, Y. Yang, M.S. Becker, L. Zanetta, E. Dejana, J.C. Gasson, . 2008. Fate tracing reveals the endothelial origin of hematopoietic stem cells. Cell Stem Cell. 3:625–636. 10.1016/j.stem.2008.09.018 19041779 PMC2631552

