# Supplementary figures and images for: EBF1 primes B-lymphoid enhancers and limits the myeloid bias in murine multipotent progenitors
Source: J Exp Med. 2022 Sep 1;219(11):e20212437. doi: 10.1084/jem.20212437 (PMC9437269; doi:10.1084/jem.20212437)

### Figure 1C

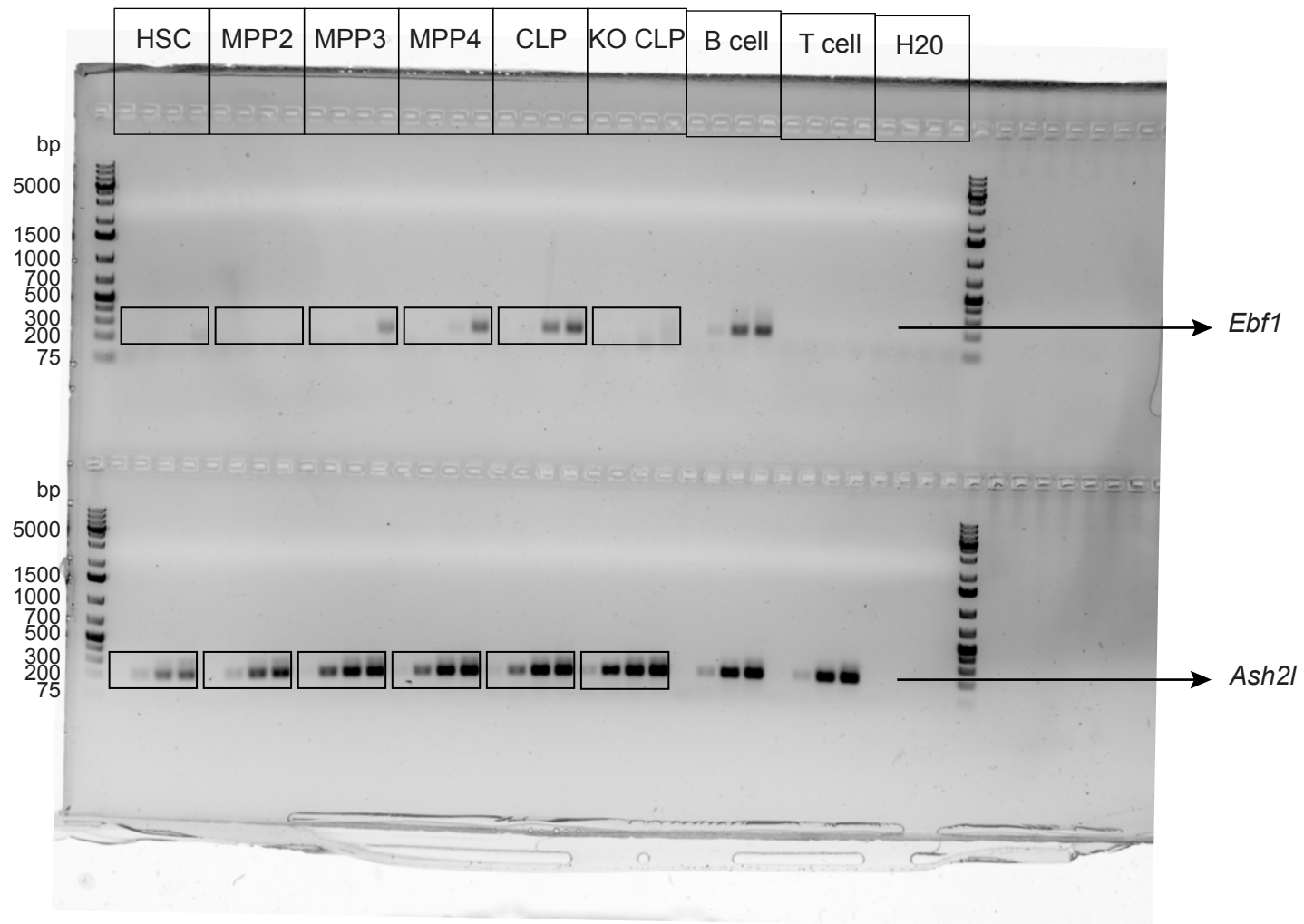

Figure 1D

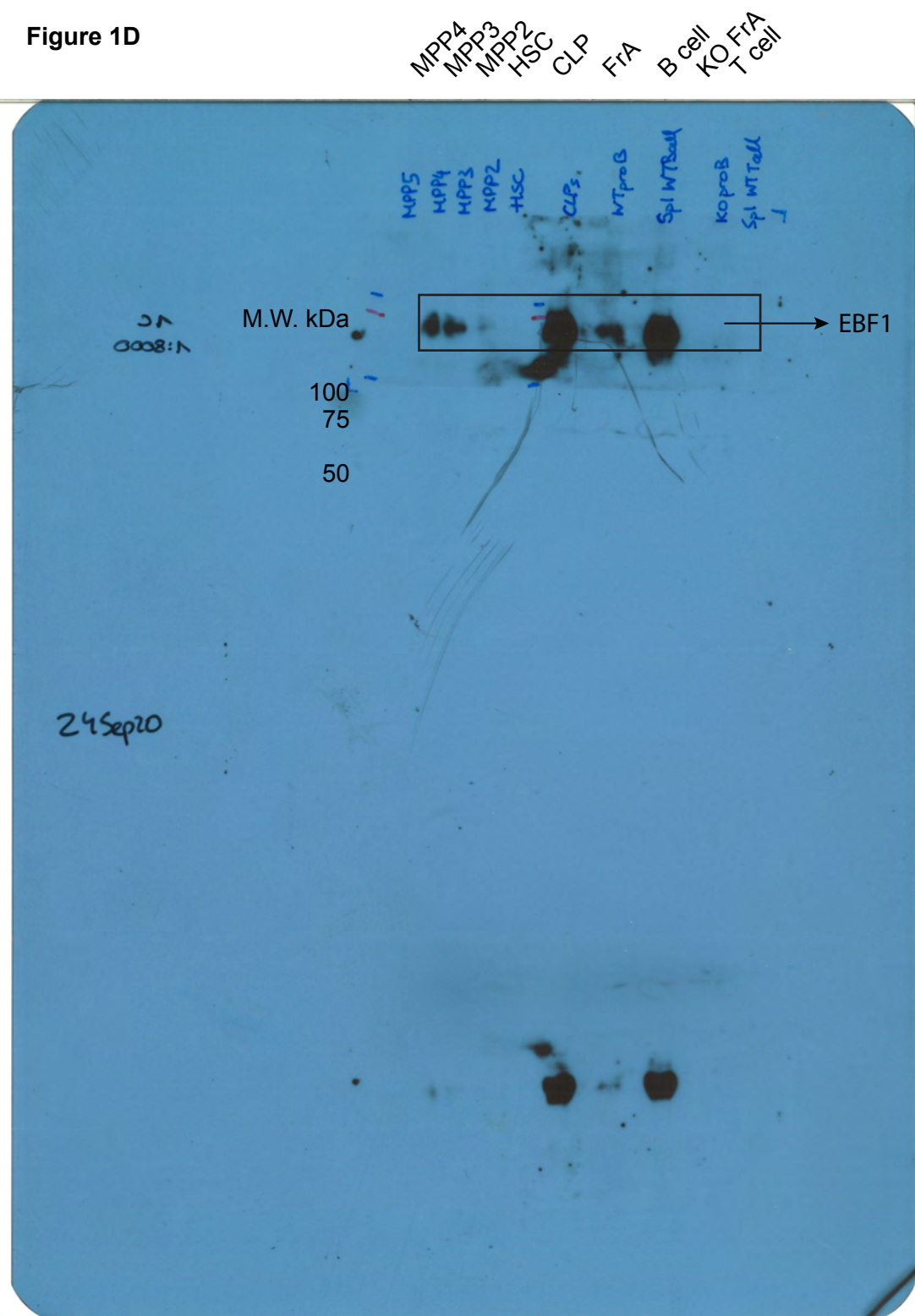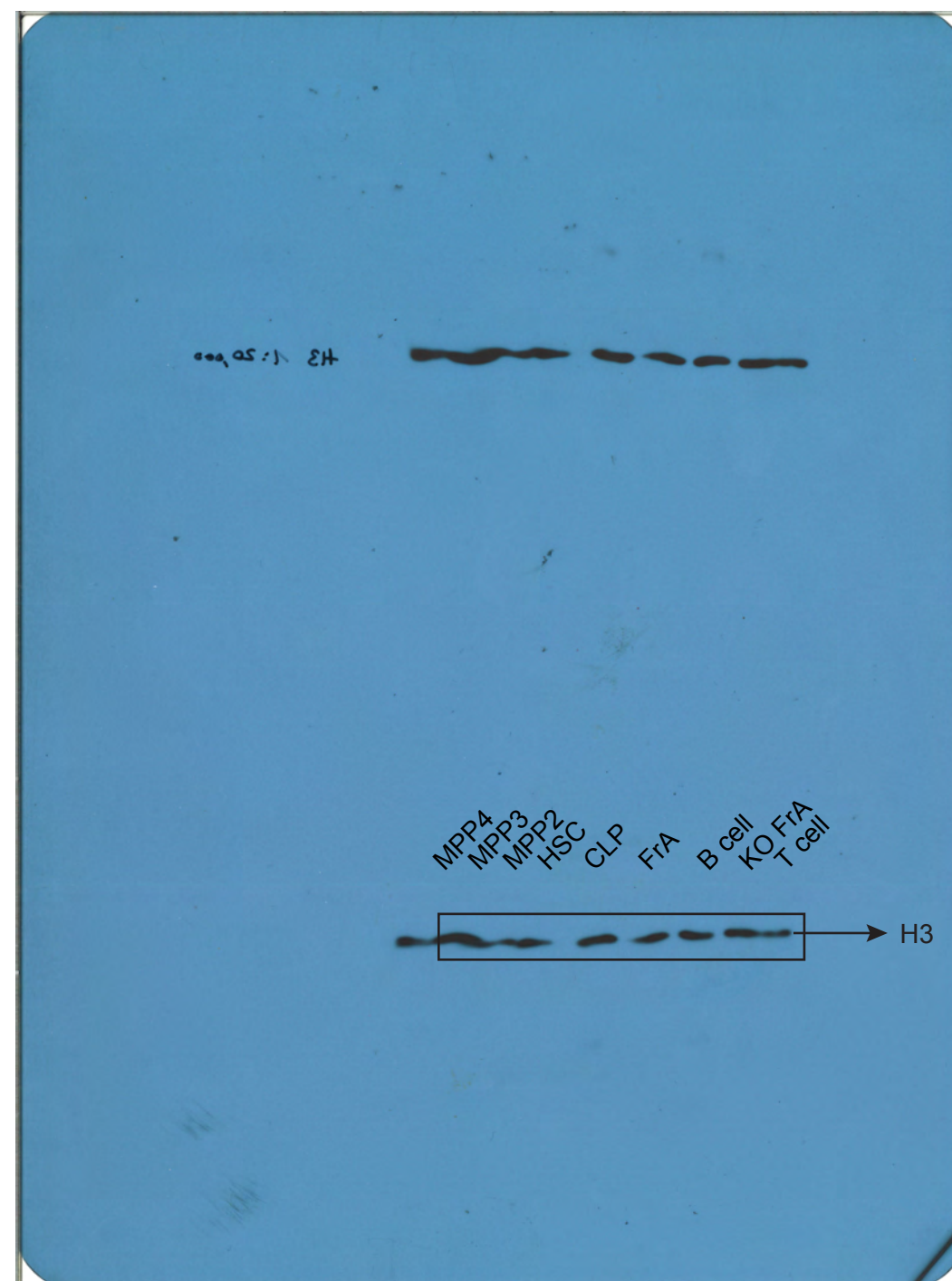

Supplement: SourceData F1 — contains original blots for Fig. 1. [file JEM_20212437_SourceDataF1.pdf]

Figure S2C

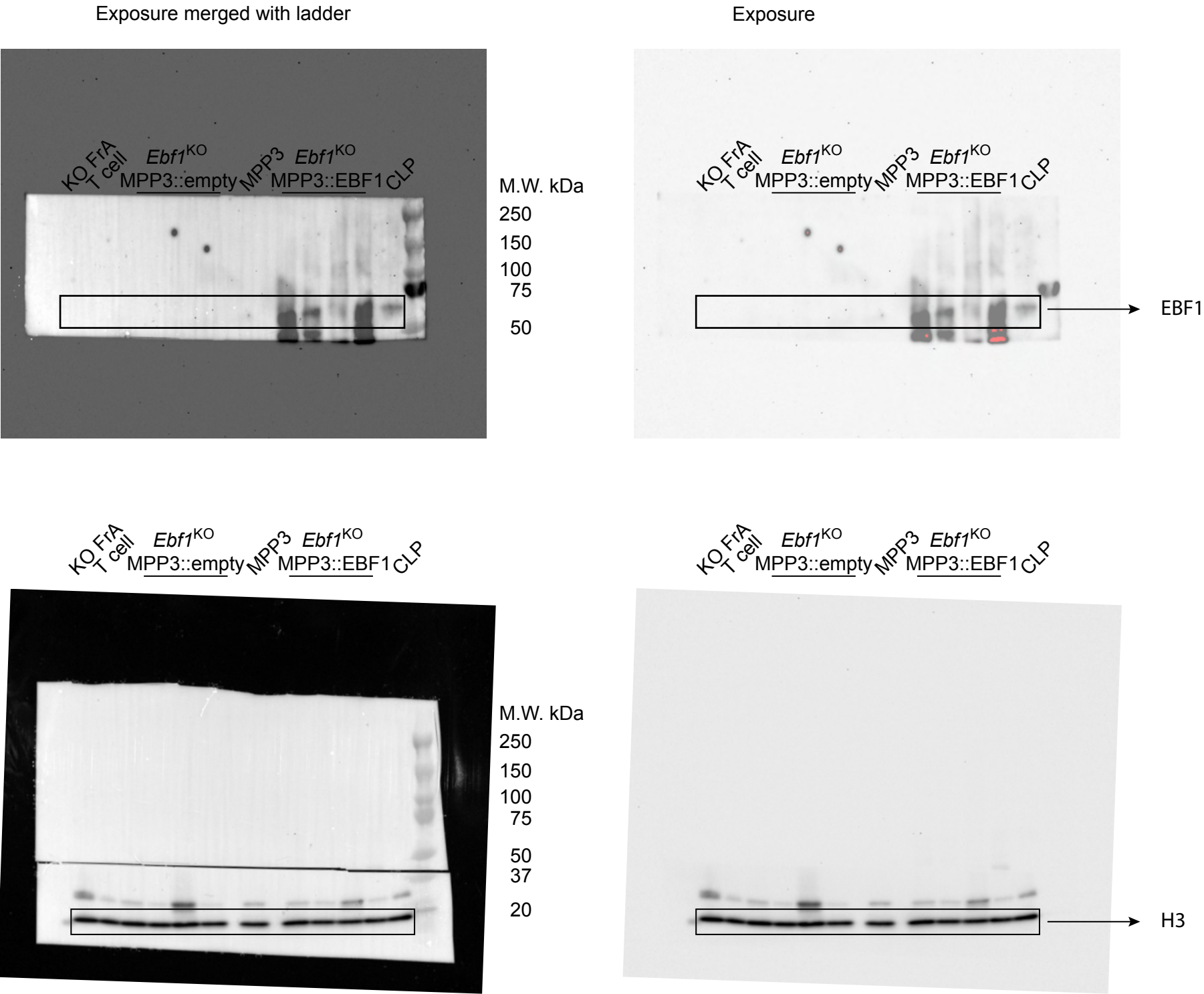

Supplement: SourceData FS2 — contains original blots for Fig. S2. [file JEM_20212437_SourceDataFS2.pdf]
